# Towards Selective Binding to the GLUT5 Transporter: Synthesis, Molecular Dynamics and In Vitro Evaluation of Novel C-3-Modified 2,5-Anhydro-D-mannitol Analogs

**DOI:** 10.3390/pharmaceutics14040828

**Published:** 2022-04-10

**Authors:** Natasha Rana, Marwa A. Aziz, Ahmed K. Oraby, Melinda Wuest, Jennifer Dufour, Khaled A. M. Abouzid, Frank Wuest, F. G. West

**Affiliations:** 1 Department of Chemistry, University of Alberta, Edmonton, AB T6G 2G2, Canada; nrana@ualberta.ca (N.R.); marwa.abdelaziz@pharma.asu.edu.eg (M.A.A.); aoraby@ualberta.ca (A.K.O.); 2 Department of Oncology, University of Alberta—Cross Cancer Institute, Edmonton, AB T6G IZ2, Canada; mwuest@ualberta.ca (M.W.); jdufour@ualberta.ca (J.D.); 3 Cancer Research Institute of Northern Alberta, University of Alberta, 2-132 Li Ka Shing, Edmonton, AB T6G 2E1, Canada; 4 Department of Pharmaceutical Chemistry, Faculty of Pharmacy, Ain Shams University, Abassia, Cairo P.O. Box 11566, Egypt; khaled.abouzid@pharma.asu.edu.eg; 5 Department of Pharmaceutical Organic Chemistry, Faculty of Pharmacy, Misr University of Science & Technology, Al-Motamayez District, 6th of October City P.O. Box 77, Egypt; 6 Department of Organic and Medicinal Chemistry, Faculty of Pharmacy, University of Sadat City, Sadat City P.O. Box 32897, Egypt

**Keywords:** GLUT, breast cancer, 2,5-anhydromannitol, 6-deoxy-6-fluoro-D-fructose (6-FDF), hydrogen bonding, simulations

## Abstract

Deregulation and changes in energy metabolism are emergent and important biomarkers of cancer cells. The uptake of hexoses in cancer cells is mediated by a family of facilitative hexose membrane-transporter proteins known as Glucose Transporters (GLUTs). In the clinic, numerous breast cancers do not show elevated glucose metabolism (which is mediated mainly through the GLUT1 transporter) and may use fructose as an alternative energy source. The principal fructose transporter in most cancer cells is GLUT5, and its mRNA was shown to be elevated in human breast cancer. This offers an alternative strategy for early detection using fructose analogs. In order to selectively scout GLUT5 binding-pocket requirements, we designed, synthesized and screened a new class of fructose mimics based upon the 2,5-anhydromannitol scaffold. Several of these compounds display low millimolar IC_50_ values against the known high-affinity ^18^F-labeled fructose-based probe 6-deoxy-6-fluoro-D-fructose (6-FDF) in murine EMT6 breast cancer cells. In addition, this work used molecular docking and molecular dynamics simulations (MD) with previously reported GLUT5 structures to gain better insight into hexose–GLUT interactions with selected ligands governing their preference for GLUT5 compared to other GLUTs. The improved inhibition of these compounds, and the refined model for their binding, set the stage for the development of high-affinity molecular imaging probes targeting cancers that express the GLUT5 biomarker.

## 1. Introduction

Breast cancer constitutes the second leading cause of cancer deaths and the most diagnosed malignancy in women [1]. A sizeable percentage of breast cancers express the protein GLUT5, the role of which is to transport the sugar fructose into the cells; on the other hand, normal breast cells do not have this protein [2]. GLUTs (facilitative hexose –transporters, from the gene family (*SLC2*)) perform the processes of the influx and efflux of monosaccharides in a gradient-dependent manner, furnishing fuels for cellular metabolic processes [3,4,5]. To date, 14 subtypes of GLUTs (GLUT1–14) have been identified and classified on the basis of sequence homology, tissue-specific expression, substrate affinities, and transport kinetic properties [6,7]. In recent years, deregulation in the expression of GLUTs has gained wide attention as this phenomenon is reported to be linked to various conditions, such as metabolic disorders, cancer, obesity, and diabetes [8,9,10,11,12]. The specific, tissue-dependent activity and overexpression of GLUTs makes them interesting diagnostic and therapeutic targets for biomarker imaging as well as the selective delivery of drugs [13,14,15,16].

D-glucose is a substrate for multiple GLUT transporters, including GLUT1, 2, 3, and 4, posing a challenge for the selective development of ligands targeting individual GLUTs [13,14,15,16,17]. In specific cases, cancer cells may switch their metabolic demand and increase their utilization of another common hexose sugar, fructose, when they have access to this alternative energy source [18]. Fructose is transported almost selectively through facilitative hexose transporter GLUT5 in millimolar (mM) concentration ranges.

GLUT5 was shown to be overexpressed in different types of cancers, including lung cancer, renal-cell carcinoma, pancreatic cancer and acute myeloid leukemia, among others [19,20,21]. Compared to estrogen receptor–positive breast cancer, triple-negative breast cancer cells and tissues express significantly higher levels of both GLUT5 protein and mRNA (as analyzed from breast cancer patient samples), making it an intriguing target for diagnosis and therapeutic application [22]. Fructose transport through GLUT5 occurs at affinities one order of magnitude lower than that of the glucose transport through its major transporter GLUT1 (*K*_4_ 1–2 mM), although still in the millimolar concentration range [23,24,25,26]. Affinities (*K_m_*) of 11–15 mM have been determined using D-[^14^C]fructose in GLUT5 (human isoform)-expressing oocytes or in brush-border membrane vesicles from rat and human intestine, the organ with the highest GLUT5 protein expression in the human organism [23,24,25,26].

Inhibition constants (*K_i_*) for bicyclic furanose analogs of GLUT5 transport were reported in the 9–32 mM concentration range as measured against [^14^C]-D-fructose uptake by GLUT5 expressed in CHO cells [27,28,29]. Early studies by Holman and co-workers demonstrated that the presence of hydroxyl groups and their stereochemical configuration on D-fructose derivatives strongly influenced GLUT5-mediated binding and transport [30,31,32,33,34,35]. These structure–activity relationship studies also revealed that hydroxyl groups in positions C-2 and C-6 only play a minor role in binding to GLUT5, rendering these carbons attractive sites for structural modifications in the design of fructose analogs [30,31,32,33,34,35]. As a result, the development of C-6-modified fluorescent and radiolabeled probes of fructose was evaluated in order to study their transport and uptake profiles through GLUT5-expressing breast cancer cells [36,37,38]. However, these probes, which lack a C-6 hydroxyl group, also underwent efflux. This was attributed to their structural unsuitability to undergo metabolic trapping inside the cells through phosphorylation by hexokinase. Removal of the C-2 hemiacetal hydroxyl group in the furanose form of fructose affords the known carbohydrate derivative 2,5-anhydromannitol (2,5-AM), which has an affinity for GLUT5 that was found to be similar to that of fructose [30,31,32,33,34,35,39,40,41]. Conjugates of 2,5-anhydro-mannitol also inhibit fructose uptake through GLUT5. Recently, we estimated an IC_50_ value against the uptake of the probe 6-[^18^F]FDF of ~20 mM for 1-deoxy-1-fluoro-2,5-anhydro-mannitol (1-FDAM), substantially better than that for D-fructose itself (~300 mM) [38]. Fluorescent derivative 1-[*N*-(7-nitrobenz-2-oxa-1,3-diazole-4-yl)amino]-2,5-anhydro-D-mannitol (NBDM) has been shown to be transported twice as efficiently as D-fructose through GLUT5 transporters, as measured in human MCF7 breast cancer cells with a *K_i_* range of 2.3–2.7 mM [42]. We previously examined a series of C-3-modified derivatives of 2,5-AM for their ability to inhibit the uptake of radiolabeled fructose in murine EMT6 mammary-carcinoma cell lines [43]. This study highlighted the importance of the strong hydrogen-bond donor properties of the C-3 substituent; in particular, two electron-deficient anilines and two amides displayed IC_50_ values comparable to or lower than that of the natural substrate, fructose. The lack of a hemiacetal at C-2 leaves 2,5-AM permanently locked in a furanose form, with a C2 symmetry that renders the C-1 and C-6 hydroxyl groups equivalent and potentially subject to phosphorylation at either site by hexokinase or ketohexokinase (fructokinase). If a reporter group could be attached via C-3 or C-4, the efflux issues noted with fructose derivatives modified at C-6 might be overcome.

With the goal of optimizing binding, transport, and metabolic trapping of 2,5-AM derivatives, here we describe efforts to prepare a series of derivatives that retain the hydrogen-bond donor capability at C-3 with a variety of functionalities and encompassing a range of steric demands. These compounds were then screened for their inhibition of the uptake of the potent and well-studied radiolabeled GLUT5 substrate, [^18^F]-6-deoxy-6-fluoro-D-fructose (6-[^18^F]FDF) in EMT6 cells. In addition, docking and molecular dynamics (MD) simulations were carried out to further evaluate the interactions between these novel compounds and GLUT5 protein at the molecular level. These studies were pursued to identify key interactions with the binding pocket that could be harnessed in the future refinement of probe structures to optimize affinity, with the eventual goal of developing next-generation molecular imaging probes to target GLUT5 through the incorporation of the appropriate reporter groups at the C-3 of the 2,5-AM scaffold.

## 2. Materials and Methods

Reactions were carried out in oven-dried glassware under a positive argon or nitrogen atmosphere unless otherwise stated. Transfer of anhydrous solvents and reagents was accomplished with oven-dried syringes or cannula. Solvents were distilled before use. Dimethylformamide (DMF) and acetonitrile (MeCN) were distilled from calcium hydride. Chemicals were purchased from Sigma-Aldrich Inc. and were used without further purification. Thin-layer chromatography was performed on glass plates preloaded with 0.25 mm silica gel matrix. Flash chromatography columns were packed with 230–400 mesh silica gel. Optical rotations were measured with Perkin Elmer 241 polarimeter, at 22 ± 2 °C. Proton nuclear magnetic resonance spectra (^1^H NMR) were recorded at 500 MHz or 700 MHz and coupling constants (*J*) are reported in Hertz (Hz). Standard notation was used to describe the multiplicity of signals observed in ^1^H NMR spectra: broad (br), multiplet (m), singlet (s), doublet (d), triplet (t), etc. Carbon nuclear magnetic resonance spectra (^13^C NMR) were recorded at 100 MHz or 125 MHz and are reported *δ* (ppm) relative to the center line of the septet from methanol-d_4_ (49.3 ppm), triplet of chloroform-d (77.2 ppm) or septet of DMSO-d_6_ (39.5 ppm). Infrared (IR) spectra were measured with a FT-IR 3000 spectrophotometer. Mass spectra were determined on a high-resolution electrospray positive ion mode spectrometer. Purity of probe compounds was measured via HPLC (C18, 4.6 × 250 mm, 0.6 mL/min; gradient elution solvent A, 0.1% TFA in water; gradient elution solvent B, 0.1% TFA in acetonitrile).

### 2.1. Synthesis

3-deoxy-3-azido-2,5-anhydro-D-mannitol (**1**) was prepared via diastereoselective ring-opening of 2,5:3,4-dianhydro-D-allitol according to the reported procedure [43,44].

3-deoxy-3-amino-2,5-anhydro-D-mannitol (**2**)

Azide **1** (0.25 g, 1.3 mmol) was dissolved in MeOH (25 mL) and 10% Pd/C (0.05 g, 20 % by weight) was added, followed by stirring the resulting heterogeneous mixture at room temperature under H_2_ atmosphere (1 atm) attained via a balloon. After stirring for 3 h, solids were removed by filtration over a celite pad and washed with DCM and MeOH (20 mL). The filtrate was completely evaporated under reduced pressure to yield amine **2** as a pale-yellow, thick syrup. The amine obtained (quant.) was used for the next step without further purification [43].

3-deoxy-3-[*N*-(5-fluoro-2,4-dinitro-phenyl)amino]-2,5-anhydro-D-mannitol (**3**)

To a stirred solution of amine **2** (0.12 g, 0.76 mmol) in dry DMF (10 mL) in an oven-dried flask maintained under N_2_ atmosphere, excess NaHCO_3_ was added. After stirring for 30 min, 1,5-difluoro-2,4-dinitrobenzene (0.19 g, 0.91 mmol) was added slowly, and the resultant heterogeneous mixture was allowed to stir at room temperature for 4 h. Upon completion of the reaction (monitored by TLC with 10% MeOH/DCM eluent system), excess NaHCO_3_ was filtered off. Solvent was then concentrated under reduced pressure yielding a brown, viscous syrup which was purified through silica gel column chromatography using a DCM/MeOH solvent mixture (gradient from 100:0 to 95:5). Fractions containing the desired product were combined and concentrated under vacuum to yield pure compound **3** as a yellow solid (0.085 g, 40%). R*_f_* 0.34 (DCM/MeOH, 90:10) [α]_D_^20^ + 29.82 (*c* 0.23, MeOH); IR (cast film) 3359, 3097, 2932, 2879, 1626, 1581, 1508, 1448, 1401, 1259, 1052 cm^−1^; ^1^H NMR (700 MHz, CD_3_OD): δ 9.05 (d, *J* = 8.0 Hz, 1H), 7.27 (d, *J* = 14.3 Hz, 1H), 4.30 (t, *J* = 5.2 Hz, 1H), 4.26 (t, *J* = 5.2 Hz, 1H), 4.08 (dt, *J* = 5.6, 4.7 Hz, 1H), 4.00 (ddd, *J* = 5.2, 3.9, 2.7 Hz, 1H), 3.81 (dd, *J* = 11.9, 4.9 Hz, 1H), 3.77 (dd, *J* = 11.9, 2.7 Hz, 1H), 3.69–3.67 (m, 1H), 3.67–3.64 (m, 1H); ^13^C NMR (176 MHz, CD_3_OD): δ 159.5 (d, *J* = 267.8 Hz), 149.0 (d, *J* = 13.9 Hz), 127.7, 126.9, 125.6, 102.2 (d, *J* = 28.1 Hz), 85.4, 83.2, 77.2, 62.0, 61.6, 61.2; HRMS (ESI) calculated for C_12_H_13_FN_3_O_8_ [M–H]^+^ 346.0692; found 346.0691, HPLC purity > 92%.

#### 2.1.1. General Procedure to Synthesize **4**, **5** and **6**

A 50 mL round-bottomed flask, maintained under N_2_ atmosphere, was charged with amine **2** (0.15 g, 0.95 mmol) and acetonitrile (15 mL). After the amine was completely dissolved, the sulfonyl chloride derivative (0.14 g, 0.74 mmol of 4-fluorobenzenesulfonyl chloride for **4**; 0.14 g, 0.74 mmol of 4-fluoro-2-(trifluoromethyl) benzenesulfonyl chloride for **5**; 0.301 g, 1.14 mmol of dansyl chloride for **6**) was added to the flask, followed by addition of excess Na_2_CO_3_. This heterogeneous mixture was allowed to stir at room temperature for 16 h. Solids were filtered off and washed with excess MeCN. The filtrate was evaporated under vacuum to yield a crude product, which was subjected to silica gel column chromatography eluted with a DCM/MeOH solvent mixture (100:0 to 92:8 for **4**; 100:0 to 95:5 for **5**; 100:0 to 92:8 for **6**). Fractions containing compounds were combined and concentrated under vacuum to yield a pure product.

3-deoxy-3-[*N*-(4-fluorobenzenesulfonamide)amino]-2,5-anhydro-D-mannitol (**4**)

Colorless oil (0.09 g, 45%). R*_f_* 0.24 (DCM/MeOH, 90:10); [α]_D_^20^ + 14.80 (*c* 0.20, MeOH); IR (cast film) 3349, 2924, 2881, 1709, 1684, 1592, 1495, 1329, 1293, 1237, 1155 cm^−1^; ^1^H NMR (500 MHz, D_2_O): δ 8.04 (dd, *J* = 8.8, 5.0 Hz, 2H), 7.42 (app t), 4.05 (t, *J* = 7.6 Hz, 1H), 3.91–3.87 (m, 1H), 3.87–3.84 (m, 1H), 3.80 (dd, *J* = 12.5, 2.8 Hz, 1H), 3.75 (t, *J* = 7.6 Hz, 1H), 3.68 (dd, *J* = 12.5, 4.9 Hz, 1H), 3.63 (dd, *J* = 12.5, 2.8 Hz, 1H), 3.46 (dd, *J* = 12.5, 4.9 Hz, 1H); ^13^C NMR (126 MHz, D_2_O): δ 166.3 (d, *J* = 253.0 Hz), 136.6, 130.7 (d, *J* = 9.8 Hz), 117.5 (d, *J* = 23.0 Hz), 83.1, 81.5, 75.7, 61.6, 61.4, 60.3; HRMS (ESI) calculated for C_12_H_16_FNO_6_SNa [M+Na]^+^ 344.0575; found 344.0575, HPLC purity > 95%.

3-deoxy-3-[*N*-(4-fluoro-2-(trifluoromethyl)benzenesulfonamide)amino]-2,5-anhydro-D-mannitol (**5**)

Colorless oil (0.08 g, 41%). R*_f_* 0.28 (DCM/MeOH, 90:10); [α]_D_^20^ + 14.00 (*c* 0.10, MeOH); IR (cast film) = 3341, 2929, 2885, 1593, 1482, 1416, 1311, 1264, 1166, 1096 cm^−1^; ^1^H NMR (700 MHz, D_2_O): δ 8.33 (dd, *J* = 9.2, 5.1 Hz, 1H), 7.81 (dd, *J* = 9.2, 2.6 Hz, 1H), 7.58 (ddd, *J* = 9.2, 7.6, 2.6 Hz, 1H), 4.02 (t, *J* = 8.3 Hz, 1H), 3.87 (ddd, *J* = 8.3, 4.6, 2.6 Hz, 1H), 3.78 (d, *J* = 4.1 Hz, 1H), 3.76 (d, *J* = 8.3 Hz, 1H), 3.73 (dd, *J* = 12.5, 2.8 Hz, 1H), 3.65 (dd, *J* = 12.5, 2.8 Hz, 1H), 3.61 (dd, *J* = 12.5, 5.0 Hz, 1H), 3.47 (dd, *J* = 12.5, 5.0 Hz, 1H); ^13^C NMR (176 MHz, D_2_O) δ 164.5 (d, *J* = 255.5 Hz), 135.5 (d, *J* = 9.5 Hz), 135.1, 130.2, 122.9 (d, *J* = 273.4 Hz), 120.2 (d, *J* = 21.4 Hz), 117.9 (d, *J* = 23.1 Hz), 82.8, 80.9, 75.1, 61.5, 61.2, 60.1. HRMS (ESI) calculated for C_13_H_14_F_4_NO_6_S [M–H]^−^ 388.0483; found 388.0487, HPLC purity > 99%.

3-deoxy-3-[*N*-(5-(dimethylamino)naphthalene-1-sulfonamide)amino]-2,5-anhydro-D-mannitol (**6**)

Yellow, sticky solid (0.11 g, 47%). R*_f_* 0.48 (DCM/MeOH, 90:10); [α]_D_^20^ + 20.90 (*c* 1.30, MeOH); IR (cast film) 3349, 2924, 2854, 1678, 1457, 1204, 1141, 1060, 790 cm^−1^; ^1^H NMR (700 MHz, CD_3_OD) δ 8.54 (dt, *J* = 8.5, 1.1 Hz, 1H), 8.34 (dt, *J* = 8.7, 0.9 Hz, 1H), 8.27 (dd, *J* = 7.3, 1.3 Hz, 1H), 7.56 (ddd, *J* = 9.8, 8.6, 7.4 Hz, 2H), 7.25 (dd, *J* = 7.6, 0.9 Hz, 1H), 3.88 (t, *J* = 6.6 Hz, 1H), 3.69–3.65 (m, 1H), 3.65–3.62 (m, 1H), 3.61 (d, *J* = 3.0 Hz, 1H), 3.54 (dd, *J* = 7.1, 6.4 Hz, 1H), 3.50 (dd, *J* = 11.9, 4.9 Hz, 1H), 3.25 (dd, *J* = 11.8, 2.8 Hz, 1H), 3.10 (dd, *J* = 11.8, 5.5 Hz, 1H), 2.87 (s, 6H); ^13^C NMR (176 MHz, CD_3_OD) δ 153.2, 137.8, 131.2, 131.1, 130.9, 130.3, 129.0, 124.3, 120.7, 116.3, 84.9, 83.7, 77.5, 63.0, 62.9, 62.3, 45.8.; HRMS (ESI) calculated for C_18_H_24_N_2_O_6_SNa [M+Na]^+^ 419.1247; found 419.1242, HPLC purity > 99%.

#### 2.1.2. General Procedure to Synthesize **7** and **8**

Amine **2** (0.13 g, 0.79 mmol) was dissolved in MeOH (15 mL) in a 50 mL round-bottomed flask, maintained under N_2_ atmosphere. To this clear solution, the isothiocyanate derivative (0.14 g, 0.95 mmol of 4-fluorophenyl isothiocyanate for **7**; 0.34 g, 0.87 mmol of fluorescein isothiocyanate isomer 1 (Sigma) for **8)** was added slowly and reaction mixture was stirred for 15 h at ambient temperature. MeOH was then removed under vacuum and the crude compound was subjected to silica gel column chromatography using a DCM/MeOH solvent mixture (gradient from 100:0 to 94:6 for **7**; 100:0 to 90:10 for **8**). Fractions containing the desired product were combined and concentrated under vacuum to yield the pure compound.

3-deoxy-3-[*N*-(1-(4-fluorophenyl)thiourea)amino]-2,5-anhydro-D-mannitol (**7**)

Brown oil (0.12 g, 62%). R*_f_* 0.25 (DCM/MeOH, 90:10); [α]_D_^20^ -5.33 (*c* 0.30, MeOH); IR (cast film) 3297, 3070, 2939, 1611, 1544, 1509, 1460, 1415, 1339, 1219, 1046 cm^−1^; ^1^H NMR (700 MHz, D_2_O): δ 7.24 (dd, *J* = 8.8, 5.0 Hz, 2H), 7.14 (app t), 4.12 (t, *J* = 6.9 Hz, 1H),), 3.95–3.92 (m, 1H), 3.92–3.88 (m, 1H), 3.76 (dd, *J* = 12.5, 3.1 Hz, 1H), 3.73–3.70 (m, 1H), 3.70–3.66(m, 1H), 3.69–3.65 (m, 1H), 3.59 (dd, *J* = 12.5, 4.4 Hz, 1H); ^13^C NMR (176 MHz, D_2_O): δ 181.5, 162.2 (d, *J* = 244.8 Hz), 129.46, 129.41, 117.2 (d, *J* = 22.1 Hz), 84.0, 82.7, 76.1, 63.0, 62.1, 61.8.; HRMS (ESI) calculated for C13H17FN2O4SNa [M+Na]^+^ 339.0785; found 339.0784, HPLC purity > 98%.

3-deoxy-3-[*N*-(3-(fluorescein)-5-yl)thiourea)amino]-2,5-anhydro-D-mannitol (**8**)

Orange, sticky solid (0.19 g, 56%). R*_f_* 0.44 (DCM/MeOH, 90:10); [α]_D_^20^ -10.64 (*c* 0.25, MeOH); IR (cast film) 3261, 2935, 2853, 1748, 1597, 1462, 1370, 1232, 1190, 1067cm^−1^; ^1^H NMR (500 MHz, CD_3_OD) δ 8.16–8.03 (m, 1H), 7.78–7.65 (m, 1H), 7.16 (d, *J* = 8.2 Hz, 1H), 6.82 (d, *J* = 8.8 Hz, 2H), 6.66 (d, *J* = 2.4 Hz, 2H), 6.57 (dd, *J* = 8.8, 2.4 Hz, 2H), 4.21 (t, *J* = 5.7 Hz, 1H), 3.98 (td, *J* = 5.8, 3.5 Hz, 1H), 3.94 (q, *J* = 3.5 Hz, 1H), 3.81 (d, *J* = 3.5 Hz, 1H), 3.78 (d, *J* = 5.6 Hz, 1H), 3.77–3.75 (m, 1H), 3.74 (d, *J* = 2.8 Hz, 1H), 3.64 (dd, *J* = 12.0, 4.2 Hz, 1H).; ^13^C NMR (126 MHz, CD_3_OD) δ 181.6, 170.2, 164.0, 154.0, 140.4, 129.6, 129.3, 129.1, 125.9, 120.2, 114.4, 111.3, 102.2, 102.0, 84.7, 83.8, 76.2, 71.7, 63.1, 62.4, 61.7; HRMS (ESI) calculated for C_27_H_23_N_2_O_9_S [M–H]^−^ 551.1133; found 551.1133, HPLC purity > 95%.

3-deoxy-3-[*N*-(4-fluorobenzamide)amino]-2,5-anhydro-D-mannitol (**9**)

To a homogenous solution of amine **2** (0.15 g, 0.92 mmol) in MeOH (10 mL), NHS ester of 4-fluoro benzoyl chloride (prepared using reported procedure [45], 0.17g, 1.1 mmol) was added. The clear solution was then stirred for 15 h at room temperature. Evaporation of MeOH under reduced pressure afforded the crude compound, which was purified using silica gel column chromatography eluted with a DCM/MeOH solvent mixture (gradient from 100:0 to 93:3). Fractions containing the desired product were combined and concentrated under vacuum to yield clear oil **9** (0.09 g, 45%). R*_f_* 0.46 (DCM/MeOH, 90:10); [α]_D_^20^ + 4.28 (*c* 0.70, MeOH); IR (cast film) 3381, 2925, 2485, 1635, 1605, 1446, 1235, 1053, 852cm^−1^; ^1^H NMR (700 MHz, D_2_O): δ 7.83 (dd, *J* = 8.7, 5.4 Hz, 2H), 7.26 (app t), 4.51 (t, *J* = 8.0 Hz, 1H), 4.30 (t, *J* = 7.9 Hz, 1H), 4.05 (ddd, *J* = 8.3, 5.4, 2.9 Hz, 1H), 4.02 (ddd, *J* = 8.0, 5.2, 2.9 Hz, 1H), 3.85 (dd, *J* = 12.5, 2.9 Hz, 1H), 3.81 (dd, *J* = 12.5, 3.0 Hz, 1H), 3.77–3.74 (m, 1H), 3.74–3.71 (m, 1H). ^13^C NMR (176 MHz, D_2_O): δ 171.2, 165.7 (d, *J* = 249.7 Hz), 130.5 (d, *J* = 9.3 Hz), 130.5, 116.5 (d, *J* = 22.2 Hz), 83.4, 81.6, 75.4, 62.3, 61.7, 58.0; HRMS (ESI) calculated for C_13_H_16_FNO_5_Na [M+Na]^+^ 308.0905; found 308.0905, HPLC purity > 95%.

3-deoxy-3-[*N*-(tert-butyl-2-amino-2-oxoethoxycarbamate)]-2,5-anhydro-D-mannitol (**I**)

In a 50 mL round-bottomed flask, maintained under N_2_ atmosphere, amine **2** (0.22 g, 1.3 mmol) was dissolved in MeOH (20 mL). NHS ester of aminoxy acetic acid (prepared using reported procedure [46], 0.75 g, 2.5 mmol) was then added in the flask and vigorous stirring was continued for 12 h at room temperature. The solvent was removed under vacuum providing the crude compound, which was subjected to silica gel column chromatography using a DCM/MeOH solvent mixture (gradient from 100:0 to 95:5). Fractions containing the desired product were combined and concentrated under vacuum to yield clear oil **I** (0.22 g, 55%). R*_f_* = 0.61 (DCM/MeOH, 80:20); [α]_D_^20^ + 22.76 (*c* 1.0, MeOH); IR (cast film) 3274, 2979, 2934, 1715, 1659, 1459, 1370, 1286, 1164, 1116, 1047, 849 cm^−1^; ^1^H NMR (700 MHz, CD_3_OD): δ 4.32 (d, *J* = 15.9 Hz, 1H), 4.27 (q, *J* = 8.3, 7.8 Hz, 1H), 4.14 (t, *J* = 7.0 Hz, 1H), 3.88 (dd, *J* = 5.2, 2.6 Hz, 1H), 3.87–3.85 (m, 1H), 3.75 (dd, *J* = 12.0, 3.0 Hz, 1H), 3.70 (dd, *J* = 12.0, 3.1 Hz, 1H), 3.63(m, 1H), 3.67–3.59 (m, 1H), 3.31 (m, 1H), 1.48 (s, 9H); ^13^C NMR (176 MHz, CD_3_OD): δ 172.0, 159.9, 85.1, 83.3, 83.2, 76.8, 76.4, 63.5, 62.9, 58.8, 28.5.; HRMS (ESI) calculated for C_13_H_24_N_2_O_8_Na [M+Na]^+^ 359.1425; found 359.1423.

3-deoxy-3-[*N*-(2-(((4-fluorobenzylidene)amino)oxy)acetamide)amino]-2,5-anhydro-D-mannitol (**10**)

The tert-butyl carbamate intermediate obtained from previous step **I**, dissolved in DMF (10 mL), was treated with 5 mL of DCM/TFA (1/1 *v*/*v*) under N_2_ atmosphere. After 8 h of stirring at room temperature, solvent was removed under vacuum, yielding the corresponding amine **II**, which was used for the next step without purification. 

2-fluorobenzaldehyde (102 µL, 0.95 mmol) and Et_3_N (159 µL, 1.1 mmol) were added to a solution of amine **II** (0.15 g, 0.60 mmol) in MeOH (5 mL). The reaction was allowed to stir at room temperature for 4 h under N_2_ atmosphere. The reaction mixture was then concentrated under reduced pressure, providing a crude residue which was purified through silica gel column chromatography using a DCM/MeOH solvent mixture (gradient from 100:0 to 93:7). Fractions containing the desired product were combined and concentrated under vacuum to yield compound **10** as a white solid (0.15 g, 70 %). R*_f_* 0.45 (DCM/MeOH, 90:10); [α]_D_^20^ +11.76 (*c* 0.50, MeOH); IR (cast film) 3326, 3108, 2925, 2873, 2486, 1653, 1511, 1467, 1230, 1157, 1078, 1018 cm^−1^; ^1^H NMR (700 MHz, CD_3_OD): δ 8.26 (s, 1H), 7.6720137.64 (m, 2H), 7.16–7.12 (m, 2H), δ 4.62 (d, *J* = 4.5 Hz, 2H), 4.30 (t, *J* = 6.5 Hz, 1H), 4.12 (t, *J* = 6.2 Hz, 1H), 3.88–3.87 (m, 1H), 3.87–3.85 (m, 1H), 3.73 (dd, *J* = 12.0, 2.8 Hz, 1H), 3.67 (dd, *J* = 11.9, 3.4 Hz, 1H), 3.64–3.61 (m, 1H), 3.61–3.59 (m, 1H); ^13^C NMR (176 MHz, CD_3_OD): δ 172.5,165.3 (d, *J* = 249.3 Hz), 151.1,130.4 (d, *J* = 8.6 Hz), 129.5 (d, *J* = 3.2 Hz), 116.8 (d, *J* = 22.3 Hz), 85.7, 84.2, 77.0, 73.8, 63.7, 63.0, 58.9; HRMS (ESI) calculated for C_15_H_19_FN_2_O_6_Na [M+Na]^+^ 365.1119; found 365.1118, HPLC purity > 98%.

3-deoxy-3-[*N*-(7-hydroxy-2-oxo-2H-chromene-3-carboxamide)amino]-2,5-anhydro-D-mannitol (**V**)

Amine **2** (0.15 g, 0.92 mmol) was dissolved in MeOH (5 mL) under N_2_ atmosphere. NHS ester of 7-hydroxy coumarin-3-carboxylic acid **IV** (prepared using reported procedure [47,48], 0.33 g, 1.1 mmol) was added to this homogenous solution, and vigorous stirring of the mixture was continued at room temperature for 15 h. MeOH was removed under reduced pressure and the crude compound was purified via silica gel column chromatography using a DCM/MeOH solvent mixture (gradient from 100:0 to 93:7). Fractions containing the desired product were combined and concentrated under vacuum to yield yellow oil **V** (0.29 g, 85%). R*_f_* 0.55 (DCM/MeOH, 90:10); [α]_D_^20^ − 2.96 (*c* 0.25, MeOH); IR (cast film) 3470, 2989, 2945, 2523, 1705, 1653, 1410, 1231, 1081, 998, 816 cm^−11^H NMR (700 MHz, CD_3_OD): δ 8.78 (s, 1H), 7.67 (d, *J* = 8.6 Hz, 1H), 6.89 (dd, *J* = 8.6, 2.3 Hz, 1H), 6.77 (d, *J* = 2.2 Hz, 1H), 4.40 (t, *J* = 6.7 Hz, 1H), 4.22 (t, *J* = 6.4 Hz, 1H), 3.98–3.94 (m, 1H), 3.94–3.90 (m, 1H), 3.79–3.76 (m, 1H), 3.76–3.73 (m, 1H), 3.70 (m, 1H), 3.67 (m, 1H)); ^13^C NMR (176 MHz, CD_3_OD): δ 165.9, 164.9, 163.1, 158.3, 149.9, 133.0, 115.7, 114.1, 112.7, 103.1, 85.6, 84.4, 77.4, 63.8, 63.0, 60.0; HRMS (ESI) calculated for C_16_H_16_NO_8_ [M–H]^−^ 350.0881; found 350.0881, HPLC purity > 98%.

3-deoxy-3-[*N*-(7-(2-fluoroethoxy)-2-oxo-2H-chromene-3-carboxamide)amino]-2,5-anhydro-D-mannitol (**11**)

Compound **V** (0.19 g, 0.57 mmol) and DMF (15 mL) were stirred until completely dissolved in a 50 mL round-bottomed flask, maintained under N_2_ atmosphere. K_2_CO_3_ (0.12g, 0.85 mmol) was then added to the flask, followed by the addition of 2-fluoro ethyl tosylate (prepared using reported procedure [49], 0.375 g, 1.7 mmol), and the resulting mixture was heated at 110 °C for 1 h. After this time, solids were filtered off and the filtrate was then concentrated under reduced pressure. The resultant crude compound was subjected to silica gel column chromatography eluted with a DCM/MeOH solvent mixture (gradient from 100:0 to 95:5). Fractions containing the desired product were combined and concentrated under vacuum to yield yellow oil **11** (0.11 g, 50%). R*_f_* 0.65 (DCM/MeOH, 90:10); [α]_D_^20^ + 10.20 (*c* 0.05, MeOH); IR (cast film) ύ = 3332, 2919, 1710, 1616, 1601, 1561, 1454, 1370, 1226, 1149, 1062, 912 cm^−1^; ^1^H NMR (700 MHz, D_2_O): δ 8.72 (s, 1H), 7.75 (d, *J* = 8.7 Hz, 1H), 7.08 (dd, *J* = 8.8, 2.3 Hz, 1H), 7.03 (s, 1H), 4.89 (dt, *J* = 47.5, 3.7 Hz, 2H), 4.52 (t, *J* = 7.2 Hz, 1H), 4.49–4.40 (m, 2H), 4.36 (t, *J* = 7.0 Hz, 1H), 4.13 (ddd, *J* = 8.2, 5.4, 3.0 Hz, 1H), 4.05 (ddd, *J* = 7.9, 5.4, 2.6 Hz, 1H), 3.86 (dd, *J* = 12.3, 2.8 Hz, 1H), 3.83 (dd, *J* = 12.4, 3.1 Hz, 1H), 3.77 (t, *J* = 5.0 Hz, 1H), 3.75 (d, *J* = 4.4 Hz, 1H); ^13^C NMR (176 MHz, D_2_O): δ 165.7, 164.1, 162.8, 158.1, 149.6, 132.7, 115.6, 115.3, 113.9, 102.0, 85.6, 84.4, 82.7 (d, *J* = 169.7 Hz), 77.4, 69.5 (d, *J* = 19.8 Hz), 63.8, 63.0, 60.1; HRMS (ESI) calculated for C_18_H_20_FNO_8_Na [M+Na]^+^ 420.1065; found 420.1069, HPLC purity > 99%.

### 2.2. In Vitro Cell Experiments

#### 2.2.1. Instruments

WIZARD2 automatic *γ*-counter (Perkin Elmer, Waltham, MA, USA)

#### 2.2.2. Buffer Solutions

Glucose-free Krebs–Ringer buffer solution (120 mM NaCl, 25 mM NaHCO_3_, 4 mM KCl, 1.2 mM KH_2_PO_4_, 2.5 mM MgSO_4_, 70 μM CaCl_2_, pH 7.4) was used for the studies with EMT6 cells. Cold phosphate-buffered saline (PBS) was used to wash the extracellular probes (137 mM NaCl, 2.7 mM KCl, 10 mM Na_2_HPO_4_, 2 mM KH_2_PO_4_). RIPA buffer (Sigma-Aldrich, St. Louis, MO, USA) was used for cell lysis.

#### 2.2.3. Radiotracer Synthesis 

Radiotracer 6-[^18^F] FDF was synthesized at the Division of Oncologic Imaging at the Department of Oncology using a GE TracerLab MX automated synthesis unit (GE Healthcare Canada Inc., Mississauga, ON, Canada). The synthesis was accomplished according to the reported, well-established radiosynthesis procedure [36,50].

#### 2.2.4. Cell Culture

Murine EMT6 mammary gland tumor cells were grown in a humidified, 5% CO_2_ incubator at 37 °C in Gibco DMEM/F-12 media supplemented with 15 mM HEPES, l-glutamine, 10% fetal bovine serum (GIBCO 12483; Gibco, Gaithersburg, MD, USA) and 1% penicillin/streptomycin with media renewal every 2 to 3 days.

#### 2.2.5. General Procedure for In Vitro Inhibition of 6-[^18^F]FDF Cell Uptake

Competition binding experiments of 2,5-anhydro-D-mannitol derivatives and d-fructose were carried out in a dose-dependent manner to determine half-maximum inhibition concentrations (IC_50_).

Solubility:

1. Fructose—freely soluble in Krebs–Ringer buffer;
2. 2,5-AM derivatives—all the samples were first dissolved in ≤0.1% DMSO and were further diluted using Krebs–Ringer buffer according to the desired concentration maintaining ≤0.1% DMSO;
3. Blank—0.1% DMSO.

EMT6 cells were grown to confluence in 12-well cell culture plates with media renewal every 2 days. One hour prior to the experiment, cell culture media was removed and the plates were washed twice with glucose-free Krebs–Ringer buffer solution. To each well, 1 mL of glucose-free Krebs–Ringer buffer solution was added and incubation at 37 °C was continued for 1 h under the glucose-free condition. After one hour, Krebs–Ringer buffer was removed. To each well 400 µL of glucose-free Krebs–Ringer buffer was added containing 0.1–0.5 MBq of 6-[^18^F]FDF and different concentrations of the 2,5-AM derivatives (solution prepared in Krebs–Ringer buffer of desired concentration) **3**–**11** (10^−8^–10^−3^ and 3 × 10^−2^ M) or fructose (10^−5^−1 M) and no compound at all for comparison (=100% uptake).

After 60 min incubation time, radiotracer uptake was stopped with 1 mL of ice-cold PBS, and the cells were washed twice with PBS and lysed in 0.4 mL radioimmunoprecipitation assay buffer (RIPA buffer). Radioactivity in the cell lysates was then determined as counts per minute (CPM) using a WIZARD2 automatic *γ*-counter (Perkin Elmer, Waltham, MA, USA) and converted to the radioactivity dose SI unit Becquerel (Bq). Data was analyzed as percentage of maximum uptake of 6-[^18^F]FDF. Graphs were constructed using GraphPad Prism 5.0 (GraphPad Software, San Diego, CA, USA) and half-maximum inhibition concentrations (IC_50_) were determined by graphical analysis of the the concentration-inhibition curves.

### 2.3. Molecular Docking

The molecular structures of the ligands were built using ChemBioDraw Ultra version 14.0 and their energy was minimized using the MMFF94x force field with ChemBio3D Ultra to produce the lowest energy conformer, followed by another preparation using the LigPrep module using the Schrödinger Small Molecule Discovery Suite. The crystal structure of the GLUT5 receptor in the inward-open conformation (PDB ID: 4YB9) was used for our computational studies. The Protein Preparation Wizard module was used to add hydrogen atoms, minimize energy and create appropriate protonation states of amino acid side chains. The Sitemap module in the Schrödinger suite was used to aid the prediction of the possible binding sites. Parameters were set to produce 5 sites which were carefully compared to the reported binding site of GLUT5. A receptor grid file was generated based on the prepared protein’s active site accounting for the most probable binding pocket. The docking algorithm Glide in extra precision (XP) was used to perform all molecular docking studies [51]. The docking generated 10 poses for each complex in which the top scoring poses were selected for further evaluation by MD simulations.

#### Molecular Dynamics Simulations

All systems were embedded in a lipid membrane of POPC lipids using the CHARMM-GUI server [52]. The systems were solvated with TIP3P water molecules and Na^+^ and Cl^−^ ions were added to create a neutral system with an ion concentration of 0.15 M and box dimensions of 100Å × 100Å × 110Å. The systems for MD were then set up using leap for Amber18 with the AmberFF14SB force field with the additional lipid14 force field for the POPC membrane. 

The ligands were parametrized using the Antechamber package in AMBER18 using the AM1-BCC charge model with the GAFF force field. The solvated systems were subject to 5000 steps of steepest descent minimization followed by 5000 steps of conjugate gradient minimization using pmemd. Initially, the systems were heated as an NVT ensemble to 100 K using a Langevin thermostat for 2500 steps while the membrane was restrained with a force constant of 10 kcal/mol, and the system’s pressure was equilibrated with as an NPT ensemble to 1 atm with gradual heating to 300 K, which was performed for 50,000 steps while restraining the lipid membrane. This was followed by a short MD run of 5 ns without lipid restraints as an NVT ensemble. The simulations were then continued for 50 ns. During the MD simulations, the equations of motion were integrated using a 2 fs time step and the atomic coordinates were saved to the trajectory producing 5000 frames. The analysis of the resultant trajectories was performed using CPPTRAJ and VMD [53,54]. Figures were rendered from snapshots using Pymol. For MD snapshots extracted from the production simulations, we calculated the enthalpic portion of the binding energy using the molecular mechanics/generalized Born surface area (MM/GBSA) method implemented in the MMPBSA.py script [55]. In MM/GBSA, the free energy change due to ligand binding is calculated as:

∆*G*_bind,solv_ = ∆*G*_MM,vac_ + ∆*G*_solv,complex_ − (∆*G*_solv,ligand_ + ∆*G*_solv,protein_) − *T*∆*S* (1)

where Δ*G*_MM,vac_ includes averaged non-bonded molecular mechanics terms (electrostatic and van der Waals) occurring between protein and ligand. Solvation terms are modeled as:

∆*G*_solv_ = ∆*G*_solv,polar_ + ∆*G*_solv,non-polar_ (2)

## 3. Results and Discussion 

### 3.1. Synthesis of C-3-Modified 2,5-AM Compounds

**Figure 1:**
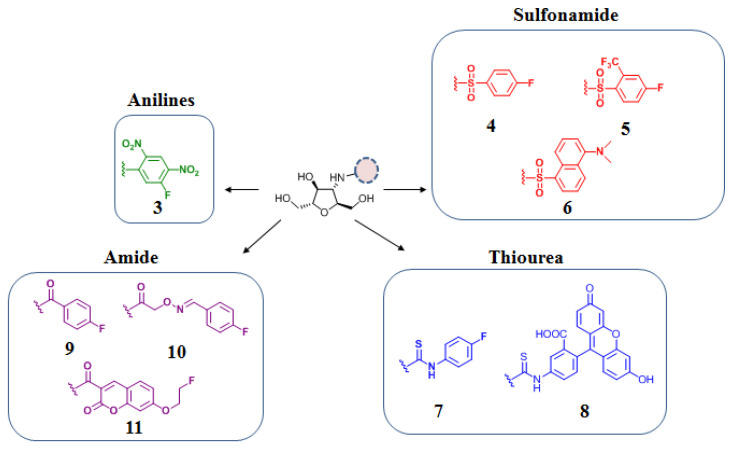
A selection of C3-modified 2,5-AM compounds.

Previously, it was observed that GLUT5 tolerates substitution of OH at C-3 of the 2,5-AM scaffold with NHR so long as its capacity for effective hydrogen bond donation is retained [56]. With an eventual goal of developing noninvasive imaging probes, we specifically targeted a new set of compounds (Figure 1) containing fluorination (with the potential for eventual radiofluorination) or fluorophores (with the potential for optical detection) and linked to the C-3 nitrogen atom by a wider range of functionalities, such as electron deficient anilines (**3**), sulfonamides (**4**, **5**, **6**), thioureas (**7**, **8**) and amide (**9**, **10**, **11**)[57]. We also selected groups ranging from simple fluorinated phenyl to larger polycyclic moieties to permit refinement of our understanding of the size limits for the molecular payloads that can be transported by the GLUT5 machinery.

**Scheme 1:**
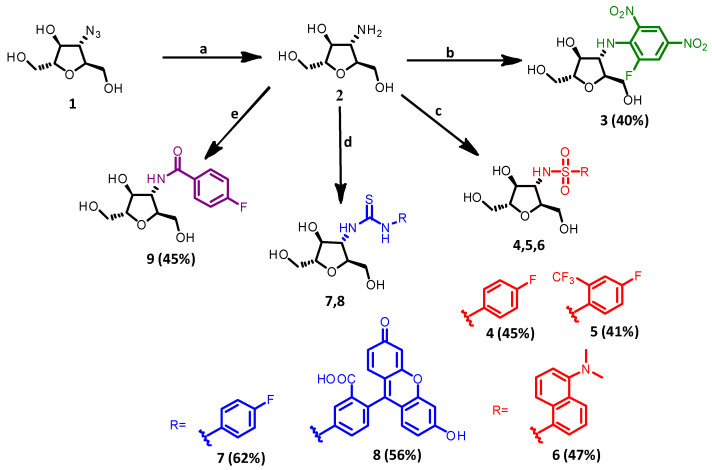
Synthesis of C3-modified 2,5-AM compounds **3**–**9**. Reagents and conditions. (**a**) Pd/C, MeOH, H_2_ (1atm), RT, 3 h, quant. (**b**) 1,5-Difluoro-2,4-dinitrobenzene, NaHCO_3_, DMF, RT, 4 h, 40%; (**c**) Sulfonyl chloride derivatives, MeCN, Na_2_CO_3_, RT, 16 h; (**d**) Isothiocyanate reagents, MeOH, RT, 15 h; (**e**) NHS ester of 4-fluorobenzoic acid, MeOH, RT, 15 h, 45%.

The synthetic route to the desired compounds began from 3-azido-3-deoxy-2,5-dianhydro-D-mannitol (**1**), synthesized according to the reported procedure [43]. Afterwards, it was reduced through Pd/C-catalyzed hydrogenation to give 3-amino-3-deoxy-2,5-anhydro-D-mannitol (**2**) which served as the key intermediate to be functionalized to several C-3-modified 2,5-AM derivatives. (Scheme 1).

Fluorinated aniline derivative **3** was prepared directly through ipso substitution reaction between the amine **2** and 1,5-difluoro-2,4-dinitrobenzene. Sulfonamide derivatives **4**, **5**, and **6** were synthesized by following the same reaction condition involving the treatment of amine **2** with different sulfonyl chloride compounds in the presence of sodium bicarbonate. Similarly, for thiourea derivatives **7** and **8**, amine **2** was treated with 4-fluorophenyl isothiocyanate or fluorescein isothiocyanate, giving the desired products in good yields. To afford the amide derivatives, different routes were employed for each target. To obtain **9**, amine **2** was directly benzoylated with NHS ester of 4-fluorobenzoic acid giving 3-deoxy-3-[*N*-(4-fluorobenzamide)amino]-2,5-anhydro-D-mannitol (**9**) in moderate yield. (Scheme 1).

**Scheme 2:**
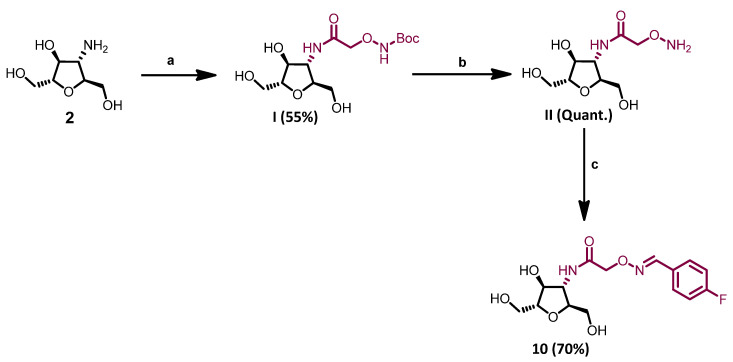
Synthetic route for compound **10**. Reagents and conditions. (**a**) (*N*-Boc aminooxy) acetic acid, *N*-*hydroxysuccinimide* (*NHS*), MeOH, RT, 12 h, 55%; (**b**) DCM/TFA (1:1), DMF, RT, 8 h, quant. (**c**) 4-F-C_6_H_4_CHO, NEt_3_, MeOH, RT, 4 h, 70%.

**Scheme 3:**
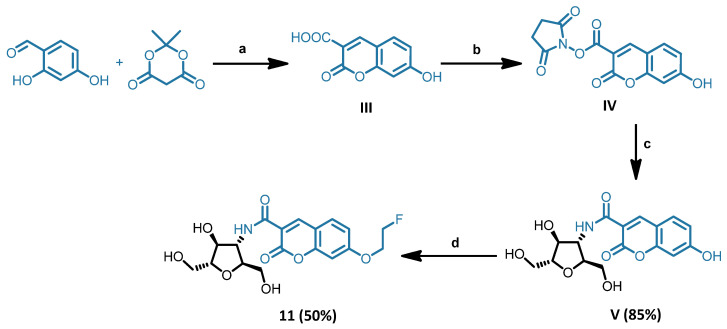
Synthetic route for compound **11**. Reagents and conditions. (**a**) H_2_O, 120 °C, 12 h, 90% (**b**) NHS, EDCI, DMF, RT, 3h, 75% (**c**) Compound 2, MeOH, RT, 15 h, 85% (**d**) 2-fluoroethyl *p*-toluenesulfonate, K_2_CO_3_, DMF, 110 °C, 1 h, 50%.

On the other hand, the synthesis of **10** and **11** were accomplished by means of the multistep sequences according to the routes depicted in Scheme 2 and Scheme 3. Synthesis of compound **10** was initiated via coupling of amine **2** with (*N*-Boc-aminooxy)acetic acid followed by deprotection of the Boc-protecting group in an acidic medium, affording **II** [46]. The resulting primary amine was then further coupled with 4-fluorobenzaldehyde to form oxime ether **10**. We also synthesized a fluorescent amide derivative, **11,** containing a coumarin moiety. The synthesis began from 2,4-dihydroxybenzaldehyde which, upon heading at reflux with Meldrum’s acid in water, provided 7-hydroxy-coumarin-3-carboxylic acid (**III)**, followed by its conversion to its activated NHS ester (**V**). The corresponding ester obtained was then treated with amino-2,5-AM **2** giving the coumarin analog of 2,5-AM (**V**). The phenolic hydroxyl group of compound **V** could be selectively alkylated with 2-fluoroethyl *p*-toluenesulfonate by taking advantage of its greater acidity relative to the alcohol moieties in the 2,5-AM scaffold, affording 3-deoxy-3-[*N*-(7-(2-fluoroethoxy)-2-oxo-2H-chromene-3-carboxamide)amino]-2,5-anhydro-D-mannitol (**11**) in moderate yield.

### 3.2. In Vitro Cell Experiments

To analyze the interaction of these novel C-3-modified 2,5-anhydro-D-mannitol analogs with GLUT5, in vitro experiments were carried out in GLUT5-expressing murine mammary carcinoma cancer cells (EMT6) to determine how the varied substitution patterns would impact GLUT5 binding. Furthermore, fructose derivative 6-FDF was used as the reference compound as it was analyzed for its uptake profile through GLUT5 in the past [38,50,58]. Inhibition experiments revealed that for D-fructose, a half-maximum inhibition concentration (IC_50_) of 322 mM was determined, while 6-FDF resulted in an IC_50_ value of 19 mM [50]. These data confirmed the millimolar concentration range for fructose transport in the utilized murine EMT6 breast cancer cells and also that 6-FDF was more than an order of magnitude more potent than D-fructose itself. In addition, D-fructose also inhibits uptake of radiolabeled D-glucose derivative 2-deoxy-2-[^18^F]fluoro-D-glucose ([^18^F]FDG) with an IC_50_ of 80 mM in murine EMT6 cells and 220 mM in human MDA-MB231 cells, confirming the presence and function of facilitative hexose transporter GLUT2 in breast cancer cells [38]. The latter data confirmed that both D-fructose and also 6-FDF are being transported through both GLUT2 and GLUT5 in breast cancer cells in a millimolar concentration range. Therefore, when designing novel specific inhibitors for the GLUT5 transporter, it would be reasonable to expect their GLUT5 transport also in a millimolar concentration range, especially when using a radiometric assay that was established with radiolabeled fructose derivative 6-[^18^F]FDF in murine EMT6 breast cancer cells and with inhibition by D-fructose and reference compound 6-FDF as internal controls [38,50].

**Figure 2:**
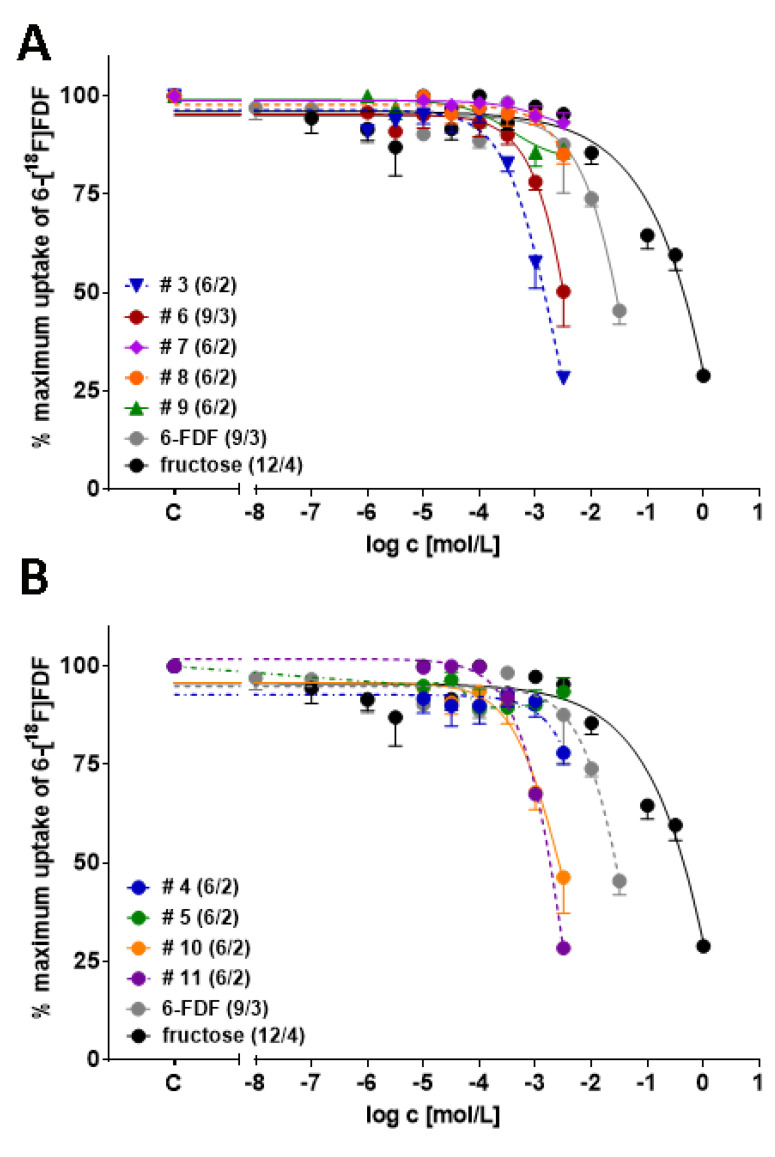
(**A**) Concentration-dependent inhibition of 6[^18^F]FDF uptake into EMT6 cells of C-3-modified 2,5-AM compounds (**3**, **6**, **7**, **8**, **9**, **11**, **6-FDF** and **fructose**) (**B**) Concentration-dependent inhibition of 6[^18^F]FDF uptake into EMT6 cells of C-3-modified 2,5-AM compounds (**4**, **5**, **10**, **11**, **6-FDF** and **fructose**). Data are shown as mean ± SEM of n data points from 2 to 4 experiments. “#” represents compound number.

Competition binding experiments against the uptake of radiolabeled 6-[^18^F]FDF in the presence of the C-3-modified derivatives were performed in a dose-dependent manner, followed by determining their half-maximum inhibition concentration (IC_50_) values (Figure 2 and Table 1). Our analysis began with the evaluation of the aniline derivative **3**, which is the fluorinated analog of the previously reported GLUT5 substrate, 3-(*N*-2,4-dinitrophenyl)amino-2,5-anhydro-D-mannitol [56] (Table 1). In comparison with reference 6-FDF, compound **3** showed a 10- to 12-fold better inhibition of 6-[^18^F] FDF uptake into EMT6 cells. This may be attributed to the electron withdrawing effect exerted by the ortho and para nitro groups on the aniline, which is expected to reduce the electron density at the amine nitrogen. This would be expected to enhance its ability to donate a hydrogen bond to complementary acceptor moieties in the binding pocket. The aromatic nitro and fluorophenyl groups may contribute further favorable interactions, as was suggested in docking studies.

Regarding the sulfonamide derivatives, compound **4** did not show a better IC_50_ value than 6-FDF, while **5** failed to inhibit 6-[^18^F] FDF uptake transport at all. On the contrary, compound **6**, connected to the bulky fluorescent dansyl group, displayed a fivefold stronger inhibitory effect on 6-[^18^F] FDF uptake compared to 6-FDF, indicating the tolerance and affinity of the GLUT5 binding pocket for greater steric bulk at C-3. On the other hand, functionalization with a thiourea handle (compound **7**) resulted in no effect on 6-[^18^F]FDF uptake into EMT6 cancer cells, while compound **8**, bearing the bulky fluorescent fluorescein group, showed a similar inhibition to 6-FDF itself.

Amide derivative **9** displayed no inhibitory activity in the selected concentration range, aligning with the results obtained for the sulfonamide and thiourea derivatives (**4**, **5** and **7**) having a small spacer between the anhydromannitol NH and aryl group. Finally, both compound **11**, with a bicyclic coumarin, and compound **10**, with a longer linker, resulted in a significant increase in the potency to inhibit 6-[^18^F]FDF uptake by ~10-fold relative to 6-FDF. Taken together, the improved inhibitory potency determined for compounds **10** and **11** suggests that an aromatic group attached with a tether and localized aromatic rings at position C-3 could be well tolerated by the GLUT5 binding pocket. Out of the novel library of 2,5-anhydro-mannitol derivatives, four compounds were found to possess IC_50_ values of 1.1 to 2.3 mM, which is one order of magnitude more potent than 1-FDAM or 6-FDF and about 2 orders of magnitude more potent than D-fructose itself [33,45]. This is in line with the findings of Tanasova et al. [42] that derivatives of 2,5-anhydro-D-mannitol can be transported with around a one-order-of-magnitude higher affinity through GLUT5 versus natural substrate D-fructose. However, the expression of the low-affinity fructose transporter protein GLUT2 by EMT6 cells also represents a potential complicating factor. As previously mentioned, 6-[^18^F]FDF is transported by both GLUT5 and GLUT2, though with higher affinity via the GLUT5 pathway; however, experiments with the 2,5-AM derivative 1-FDAM suggest that it may not show a similar uptake profile, indicating that they may be more specific for GLUT5 transport only [21,36,39]. Complete assessment of the extent of the inhibition of 6-[^18^F]FDF uptake in EMT6 cells by the 2,5-AM analogs discussed here requires further evaluation of their specific interactions with GLUT2, which is beyond the scope of the present study.

The results described above compare well with previous efforts to identify GLUT5 inhibitors. Natural plant products such as astragalin-6-glucoside and rubusoside were shown to possess IC_50_ values of 1.8 mM and 10.3 mM, as measured against D-[^14^C]fructose [59]. Also, MSNBA (N-(4-methanesulfonyl-2-nitrophenyl)-2H-1,3-benzodioxol-5-amine) generated as a specific GLUT5 inhibitor from a virtual screening library, exhibits an IC_50_ value against D-[^14^C] fructose of 5.8 mM in MCF7 human breast cancer cells and 0.10 mM in a proteoliposome GLUT5 expression system [60].

### 3.3. In Silico Studies

**Figure 3:**
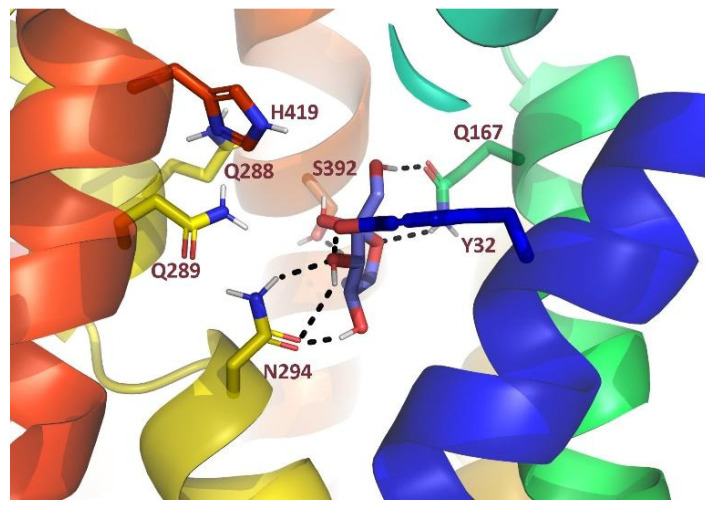
Docking pose and putative interactions of fructose with GLUT5. (Hydrogen bonds are shown as black dotted lines).

We sought to examine the origins of the substantially different 6-[^18^F]FDF inhibitory properties detected for the structurally related 2,5-AM derivatives 3–11. We started by docking each of the compounds, as well as fructose, into the central cavity at the transmembrane domain (TMD) of the open, inward-facing conformation of GLUT5 (PDB accession: 4YB9) [27]. The docking of fructose showed hydrogen bonding interactions with the C-domain residues of helix 7, namely Q167, Y32, S392 and N294, in agreement with poses and interactions previously reported by Nomura and others as essential residues for fructose binding and uptake (Figure 3) [27,61,62].

**Figure 4:**
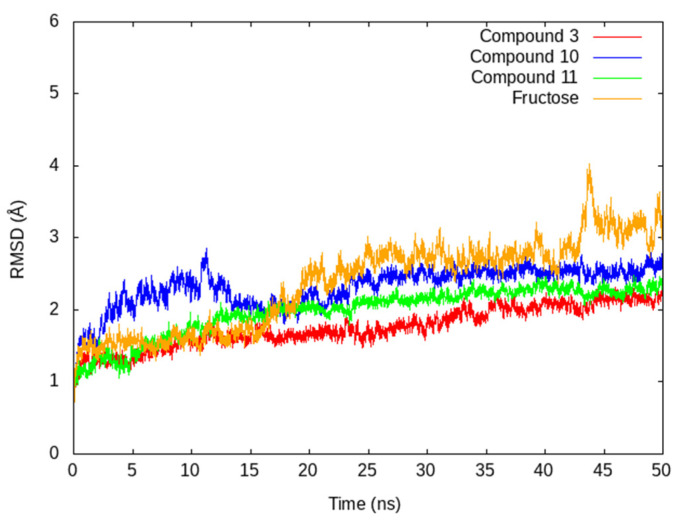
Root mean square deviations (RMSD) of ligand–GLUT5 complex systems as a function of simulation time.

To obtain a more accurate representation and validation of the binding poses suggested by molecular docking, we then chose three compounds (**3**, **10** and **11)** for further computational studies by molecular dynamics (MD) to explore whether the compounds remain in the proposed binding pocket. We expected these molecular dynamics simulations to provide insights into the impact of structural changes at the C-3 position on the ability of these molecules to occupy the GLUT5 fructose-binding pocket. The selected compounds were submitted for a 50 ns long MD simulation to simulate atomic motions and to validate the stability and the poses of docked ligands. The MD simulations were carried in a membrane environment of POPC lipids to mimic the environment of the protein (Figure S1) by using AMBER18 and the ff14SB force field combined with the GAFF force field [63,64]. Analysis of the MD trajectories revealed that all complexes equilibrated at around 30 ns with average RMSD values of 2.7 Å, 2.1 Å, 2.7 Å and 2.3 Å for fructose, compounds **3**, **10** and **11**, respectively (Figure 4). However, some fluctuations in the RMSD of fructose were observed at around 45 ns in the simulation, which could be the result of a low hydrogen bond occupancy with binding residues at this time in the simulation and the probability that the compound is moving down the cavity for uptake, as can be seen during the process of the simulation trajectory using VMD software.

Hydrogen bond occupancies are usually used to study the interactions between the ligands and target proteins [65]. Residue Q167 has been previously suggested to be crucial for the recognition, interaction and specificity of fructose to the GLUT5 protein compared with other GLUTs [27]. Analysis of the hydrogen bond occupancies of the complexes through the MD trajectories revealed that fructose formed hydrogen bonds with residues Q167, N325, Q289 and S392 in agreement with previous reports (Figures S2 and S3) [27,60]. Similarly, compounds **3**, **10** and **11** formed hydrogen bonds with Q167, Q289 and Q288 (Figure S3).

Further investigation of hydrogen bonds formed throughout the simulation trajectories showed that compound **3**, which exhibited the most potent IC_50_ value and strongest interactions with GLUT5, formed extra hydrogen bonds between the oxygen of the nitro group and the backbone NH of G163, in addition to hydrogen bonds between the H419, Y32 and N294 side chains and the sugar part of the molecule (Figure S4, Video S1). We used clustering in AMBER tools via the average-linkage algorithm to obtain a representative structure of the last 20 ns of the simulation for all ligands. Examination of this representative structure for compound **3** revealed a pi–cation interaction frequently observed between the positively charged nitro group nitrogen atom and the His imidazole sidechain which might contribute to the stability of the complex and the greater inhibition of the uptake of this compound. Similarly, hydrogen bonds occurred between the amide oxygen and C4 hydroxyl of compound **10** with N294, which might also contribute to the better inhibitory activity of this class of C-3-substituted 2,5-AM derivatives (Figure S5). Three more hydrogen bonds were observed between the amide NH at C-3, the oxygen of the (-O-N=CH-) and the C-6 hydroxyl of compound **10** with Q167. Similarly, the amide NH at C-3 of compound **11** formed the essential hydrogen bond contact with Q167 (Figure S6). It is worth noting that hydrogen bonding interactions between fructose hydroxyl groups and N294 were previously reported to be essential to the interaction with the GLUT5 protein structure [66]. These observations might contribute to the ability of the C-3-modified 2,5-AM derivatives to be involved in hydrogen bond contacts with important GLUT5 binding residues and the tolerability of the binding pocket to accommodate a variety of sterically demanding groups (e.g., coumarins, nitrophenyl, and others; Figure S7). The stability of the simulations was confirmed by the radius of gyration for all the simulated complexes (Figure S8). Root mean square fluctuations (RMSF) showed similar fluctuation patterns to the protein backbone of GLUT5 in complex with all compounds analyzed (Figure S9).

In order to get better insight into the contributions of the selected ligands to the stability of their complexes with GLUT5, we employed the MMGBSA method to measure the free binding energy of the complexes [44,55]. The calculated binding energies were similar for the GLUT5 complexes with compounds **3**, **10** and **11,** with −48.64 ± 2.3 kcal mol^−1^, −43.30 ± 3.2 kcal mol^−1^ and −45.26 ± 2.9 kcal mol^−1^, respectively, and were significantly lower than that calculated for fructose (−16.95 ± 2.3 kcal mol^−1^). It is worth mentioning that the free binding energy calculated for fructose is similar to that obtained by Ainsley et al. [61,62]. These findings could be correlated to the observed activity of each ligand in stabilizing the GLUT5 complexes with good insight into the possible future modification of the 2,5 AM to design more potent inhibitors. Overall, the computational results agree with the observed inhibitory activities of these novel probe molecules. Moreover, the MD studies support previous reports of the essential residues involved in the binding of fructose and its analogs to GLUT5 [61,62].

## 4. Conclusions

This study sought to develop a more thorough understanding of the structural factors influencing the binding of 2,5-anhydromannitol derivatives to the fructose transporter GLUT5. Competition of novel C-3-modified 2,5-anhydro-D-mannitol analogs against 6-[^18^F]FDF was compared in breast cancer cells to fructose and the non-radioactive 6-FDF, which is known to be substantially more potent than fructose itself. Several of the compounds—modified with payloads of varying steric demand—displayed concentration-dependent inhibitory effects on the cellular uptake of 6[^18^F]FDF at levels 100-fold or more than the natural GLUT5 substrate, fructose. Involvement of the low-affinity glucose/fructose transporter GLUT2 in these results cannot be ruled out, but this transporter is expected to be a minor contributor to overall uptake. Computational studies indicate that the most potent inhibitors capture several important noncovalent interactions in the GLUT5 fructose binding site, and MD simulations suggest that these interactions are more robust than those of the natural substrate fructose. These results help refine the understanding of the structural requirements of the GLUT5 transport machinery with respect to the molecular cargoes attached to fructose mimics that are tolerated by the protein. Starting with one of the most active inhibitors from this initial screening, it may be possible to further optimize and radiolabel an advanced candidate for molecular imaging of breast cancer via PET, fluorescence or dual-imaging modalities. Since elevated GLUT5 expression and abnormal fructose metabolism are associated with several cancers and other diseases, broader applications beyond breast cancer may ultimately be possible.

## Figures and Tables

**Table 1:** Half-maximum inhibition concentrations (IC_50_) for C-3-modified 2,5-AM compounds against 6-[^18^F]FDF uptake into EMT6 cells. Data shown as mean ± SEM from n data points out of 2–4 experiments; n.d.—not determined.

Compound	n/x	IC_50_ (mM)
3	6/2	1.10 ± 0.17
4	6/2	~6 (estimated)
5	6/2	n.d.
6	9/3	2.29 ± 0.18
7	6/2	n.d.
8	6/2	~20 (estimated)
9	6/2	n.d.
10	6/2	2.31 ± 0.35
11	6/2	1.71 ± 0.08
6-FDF	9/3	17.02 ± 0.75
Fructose	12/4	342 ± 74

## Data Availability

Not applicable.

## Supplementary Materials

The following supporting information can be downloaded at: https://www.mdpi.com/article/10.3390/pharmaceutics14040828/s1, Figure S1: Embedded GLUT5 complex used for molecular dynamics simulations; Figure S2: 3D snapshot of fructose during MD simulation; Figure S3: Hydrogen bond occupancies of compounds used in MD simulations; Figure S4: Snapshot of compound **3** during MD simulation; Figure S5: 3D snapshot of compound **10** during MD simulation; Figure S6: 3D snapshot of compound **11** during MD simulation; Figure S7: Compound **11** accommodated in the GLUT5 binding pocket; Figure S8: Radius of gyration fluction versus time of GLUT5 complexed with compounds **3, 10**, **11**, and fructose; Figure S9: RMSF as a function of B-factor and resitues of GLUT5 in complex with compounds **3**, **10**, **11**, and fructose; NMR spectra; FTIR spectra; HRMS data; Video S1: Compound **3** MD Simulation 50 ns.
