# Supplementary material for: Towards Selective Binding to the GLUT5 Transporter: Synthesis, Molecular Dynamics and In Vitro Evaluation of Novel C-3-Modified 2,5-Anhydro-D-mannitol Analogs
Source: Pharmaceutics. 2022 Apr 10;14(4):828. doi: 10.3390/pharmaceutics14040828 (PMC9032776; doi:10.3390/pharmaceutics14040828)

Electronic Supplementary Information

Towards selective binding to GLUT5 transporter- synthesis, molecular dynamics and *in vitro* evaluation of novel C-3 derived 2,5-anhydro-D-mannitol analogs.

Natasha Rana 1,2,3, Marwa A. Aziz 1,4, Ahmed K. Oraby 1,5, Melinda Wuest 2,3, Jennifer Dufour 2,
Khaled A. M. Abouzid 4,6, Frank Wuest 2,3,* and F. G. West 1,3,*

1 Department of Chemistry, University of Alberta, Edmonton, AB T6G 2G2, Canada; nrana@ualberta.ca (N.R.); marwa.abdelaziz@pharma.asu.edu.eg (M.A.A.); aoraby@ualberta.ca (A.K.O.)
2 Department of Oncology, University of Alberta—Cross Cancer Institute, Edmonton, AB T6G IZ2, Canada; mwuest@ualberta.ca (M.W.); jdufour@ualberta.ca (J.D.)
3 Cancer Research Institute of Northern Alberta, University of Alberta, 2-132 Li Ka Shing,
Edmonton, AB T6G 2E1, Canada
4 Department of Pharmaceutical Chemistry, Faculty of Pharmacy, Ain Shans University, Abassia,
Cairo P.O. Box 11566, Egypt; khaled.abouzid@pharma.asu.edu.eg
5 Department of Pharmaceutical Organic Chemistry, Faculty of Pharmacy,
Misr University of Science & Technology, Al-Motamayez District, 6th of October City P.O. Box 77, Egypt
6 Department of Organic and Medicinal Chemistry, Faculty of Pharmacy, University of Sadat City,
Sadat City P.O. Box 32897, Egypt
** Correspondence: wuest@ualberta.ca (F.W.); fwest@ualberta.ca (F.G.W.)*

Supplementary figures

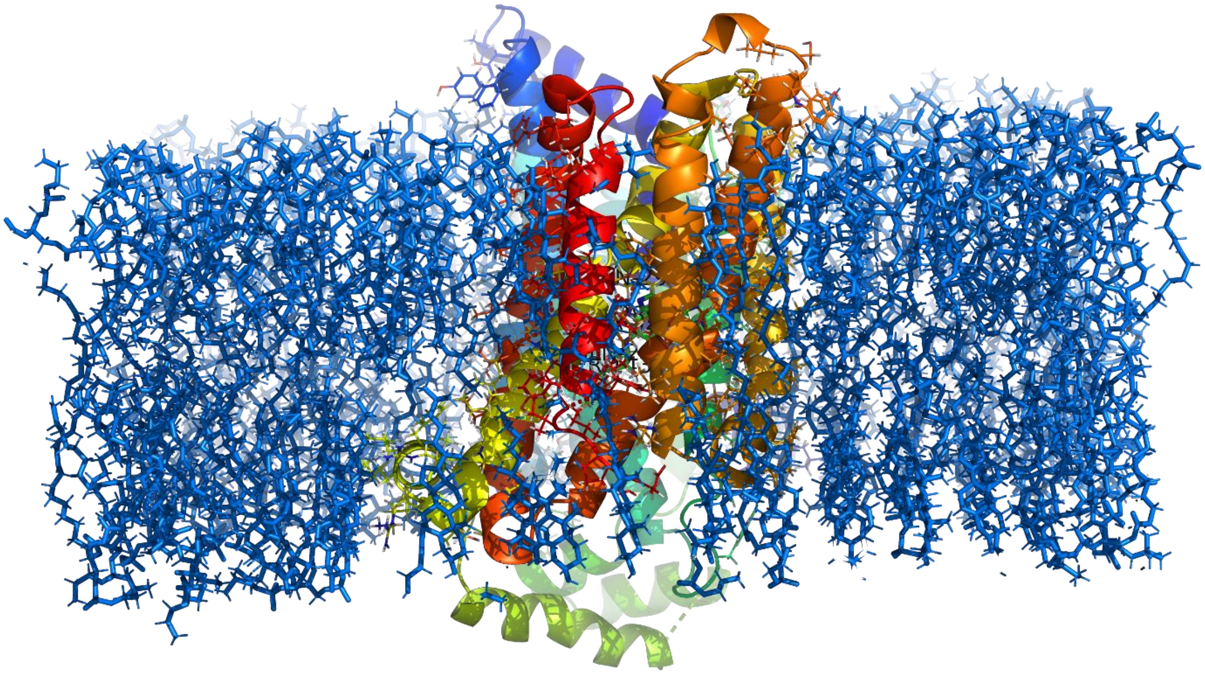

Figure S1: Embedded GLUT5 complex (colored ribbons) in a lipid membrane (blue sticks) used for molecular dynamics simulations. (Figure generated in Pymol)

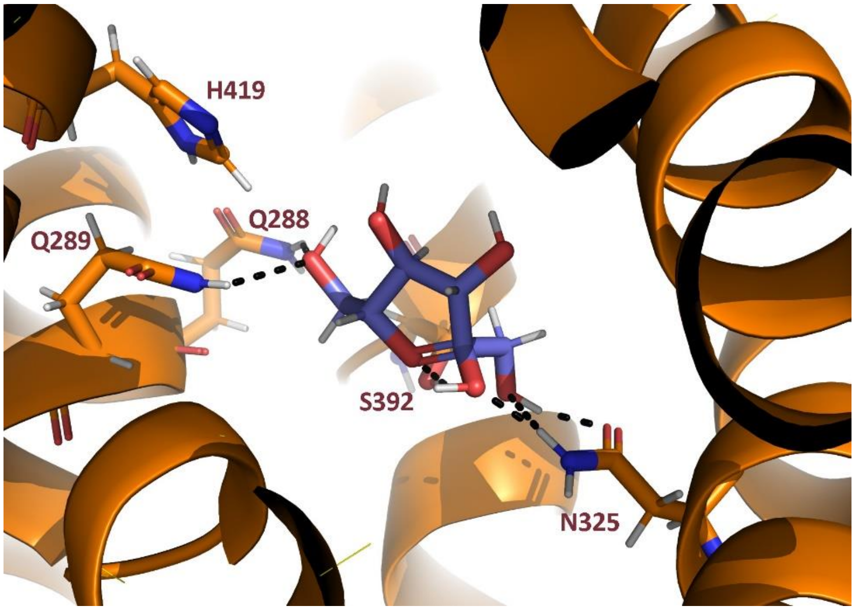

Figure S2: 3D snapshot of fructose during MD simulation. Hydrogen bonds are shown as black-dashed lines.

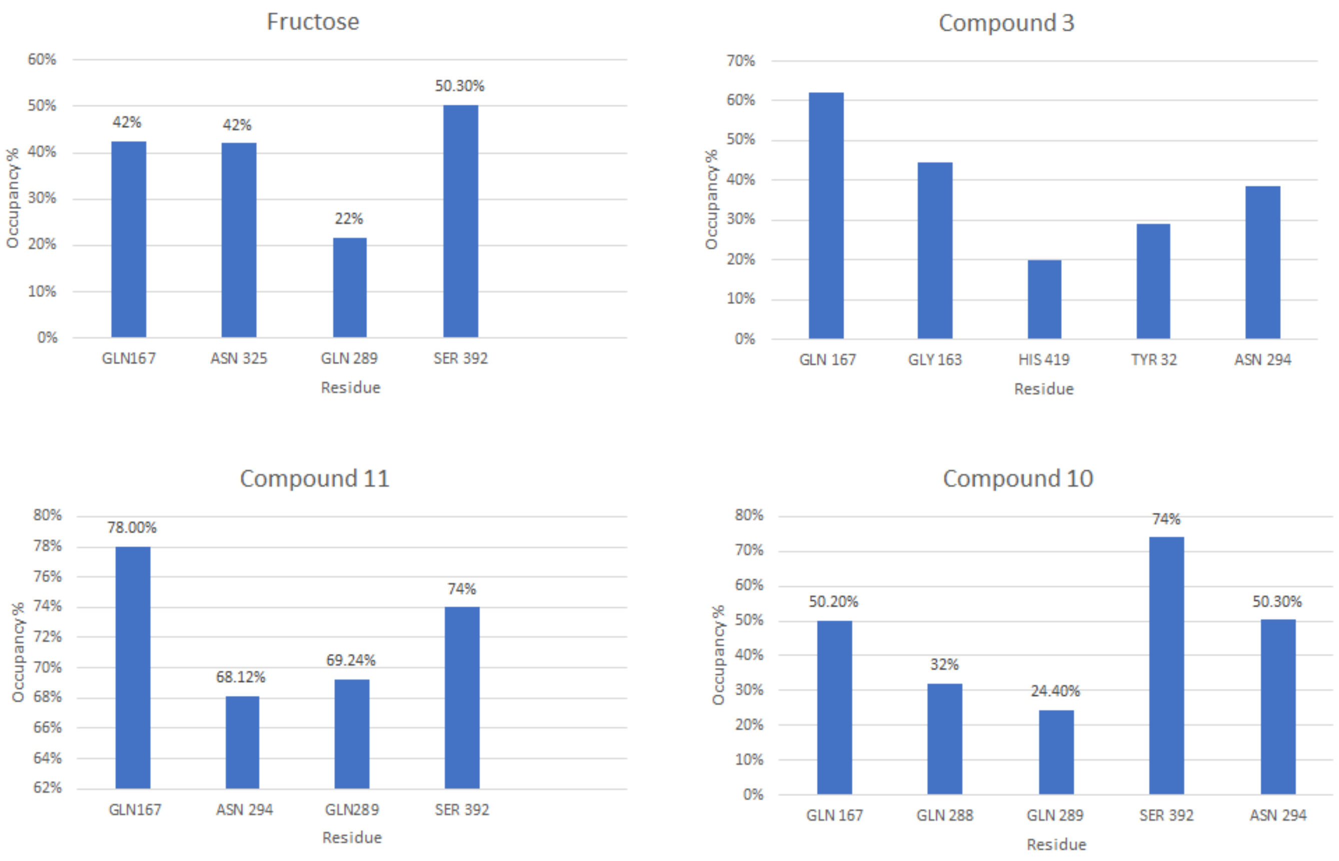

Figure S3: Hydrogen bond occupancies of compounds used in MD simulations.

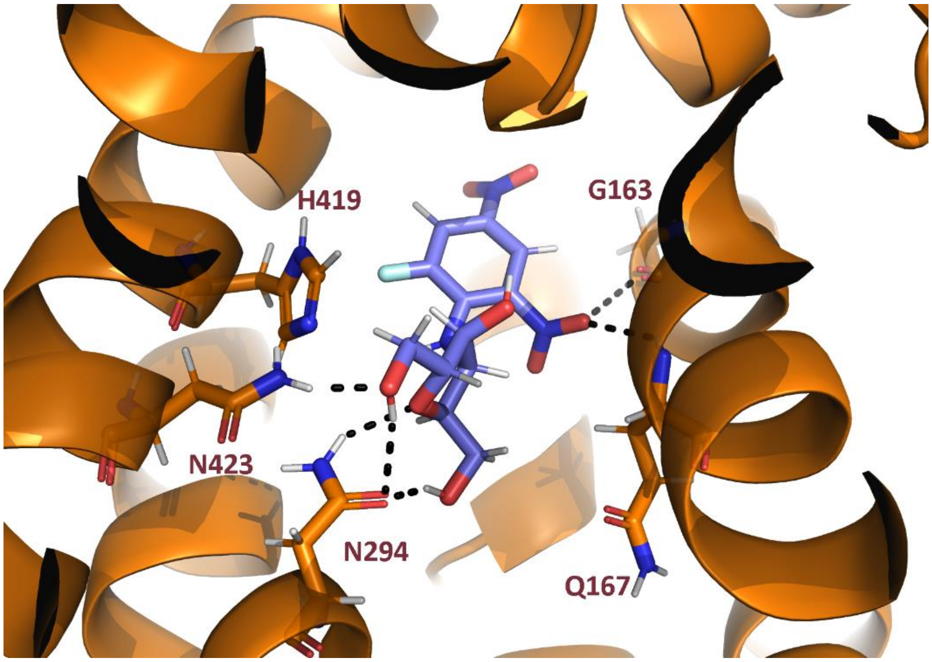

Figure S4: Snapshot of compound **3** during MD simulation. Hydrogen bonds are shown as black-dashed lines.

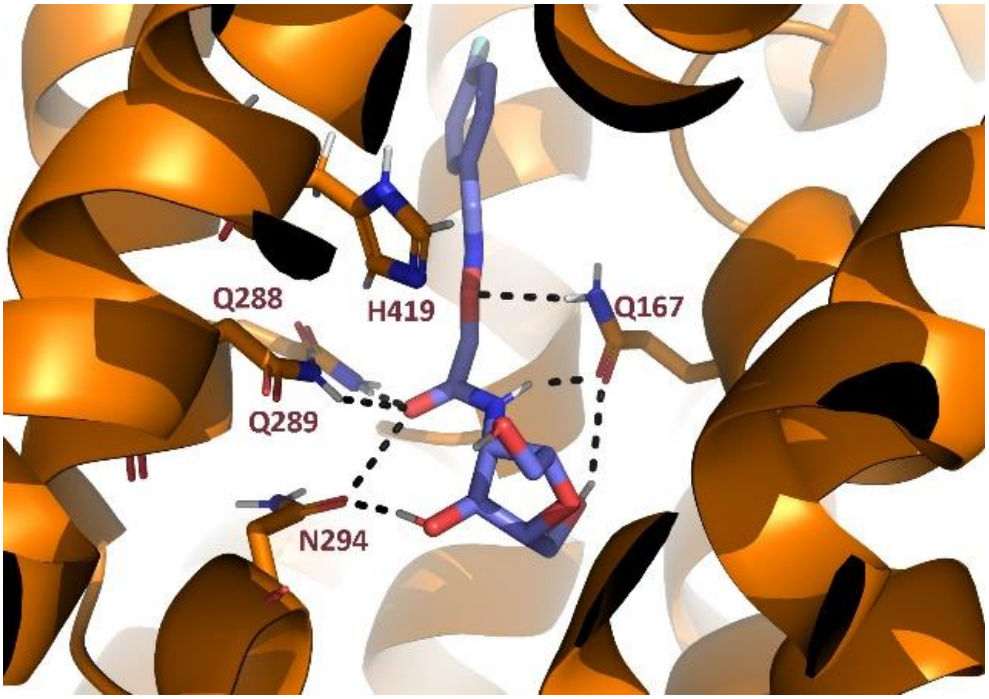

Figure S5: 3D snapshot of compound **10** during MD simulation. Hydrogen bonds are shown as black-dashed lines.

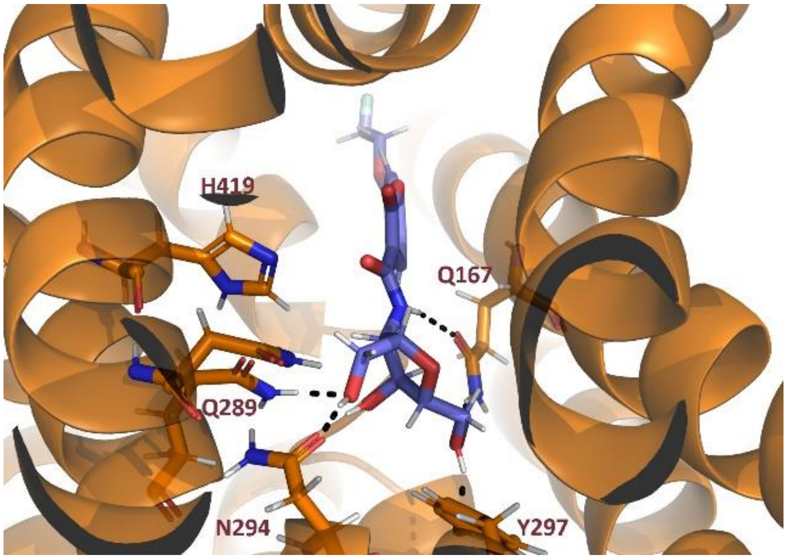

Figure S6: 3D snapshot of compound **11** during MD simulation. Hydrogen bonds are shown as black-dashed lines.

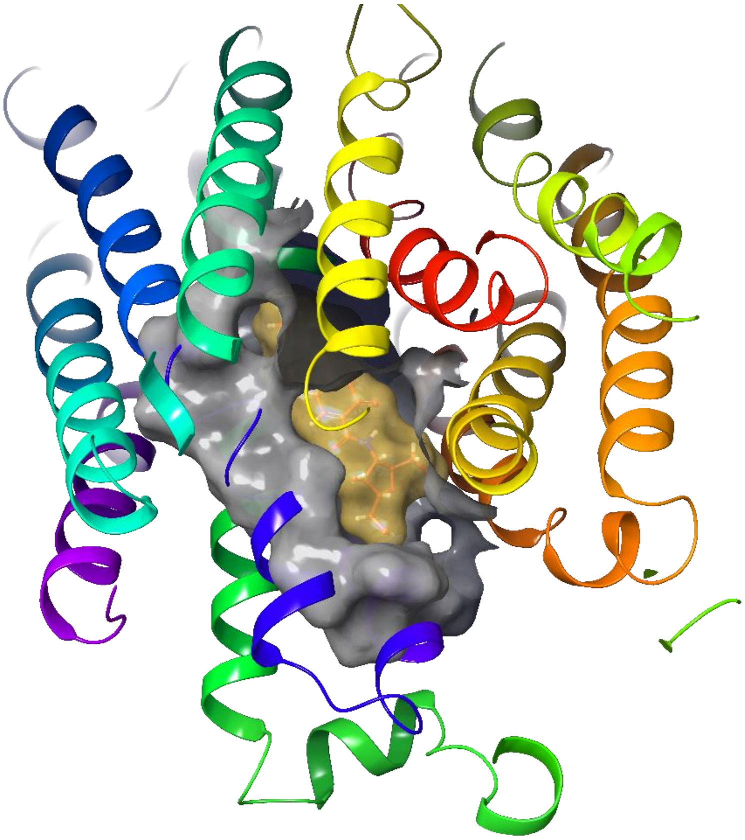

Figure S7: Compound 11 (orange surface) acommodated in the GLUT5 binding pocket (gray surface).

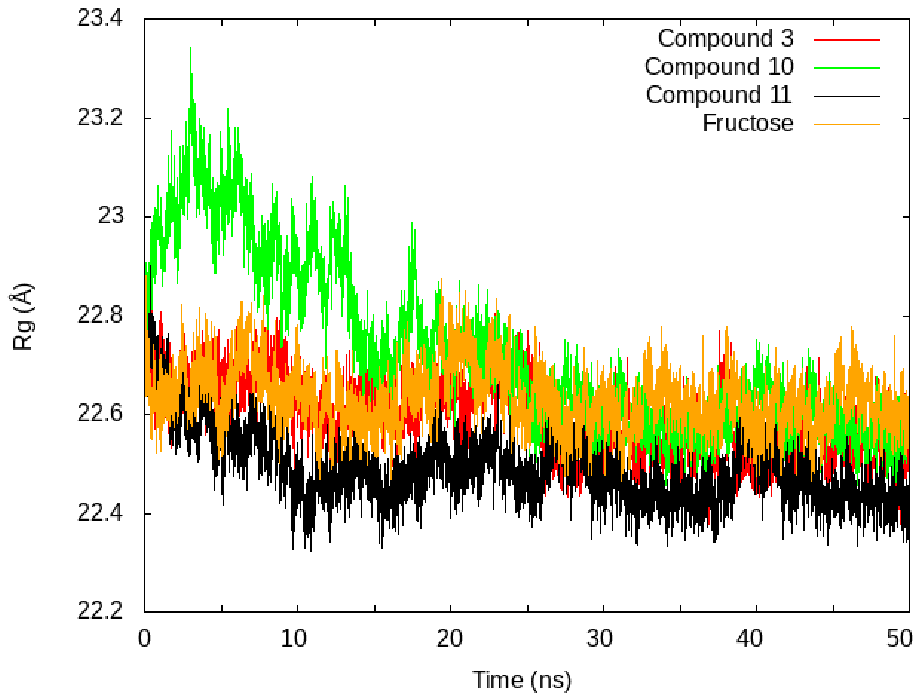

Figure S8: Radius of gyration (Rg) fluctuation versus time of GLUT5 complexed with compounds 3,10, 11, and fructose.

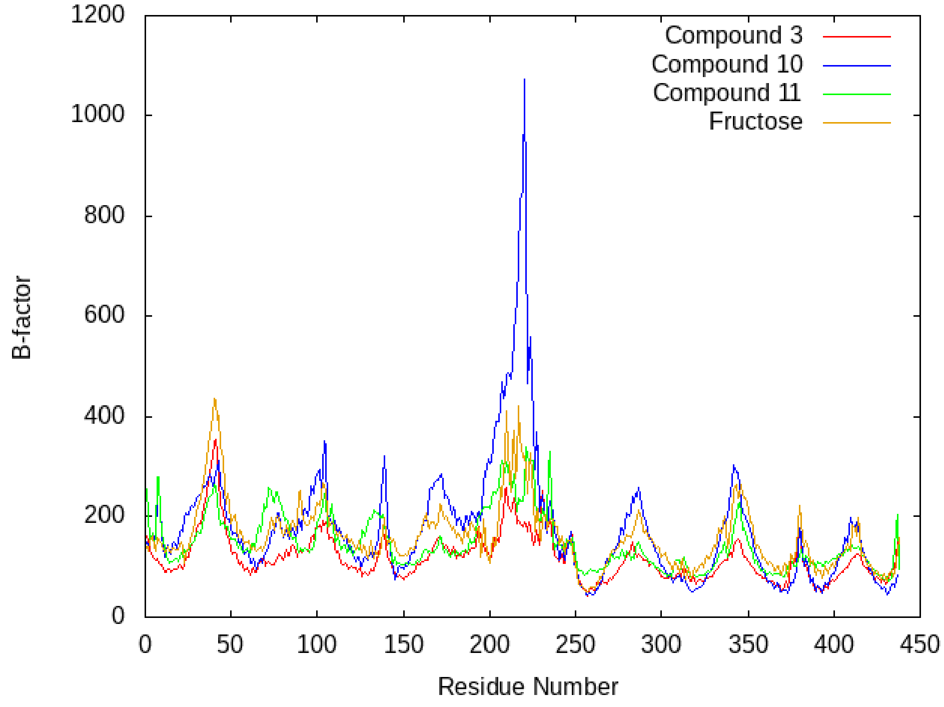

Figure S9: RMSF as a function of B-factor and residues of GLUT5 in complex with compounds 3,10,11, and fructose.

NMR Spectra

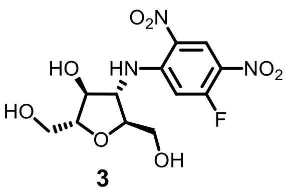
O2N
HO
HN
NO2
HO
O
F
OH

3

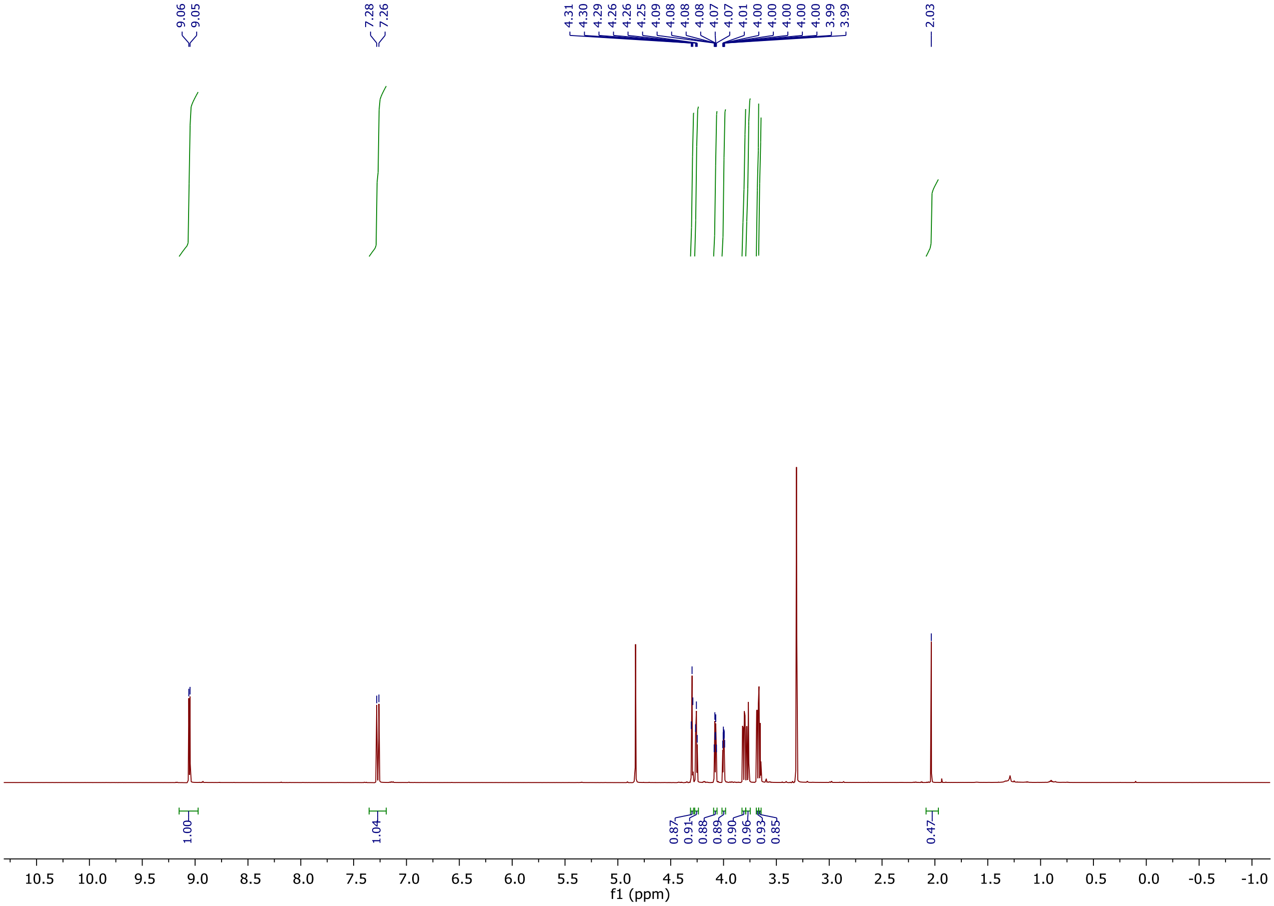
9.06
9.05
7.28
7.26
4.31
4.30
4.29
4.26
4.26
4.25
4.09
4.08
4.08
4.07
4.07
4.01
4.00
4.00
4.00
4.00
4.00
3.99
3.99
2.03
1.00
1.04
0.87
0.91
0.88
0.89
0.90
0.96
0.93
0.85
0.47
10.5
10.0
9.5
9.0
8.5
8.0
7.5
7.0
6.5
6.0
5.5
5.0
4.5
4.0
3.5
3.0
2.5
2.0
1.5
1.0
0.5
0.0
-0.5
-1.0
f1 (ppm)

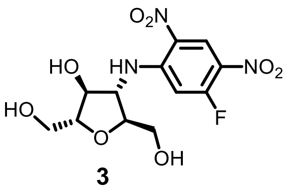
O2N
HO
HN
NO2
HO
F
O
OH

3

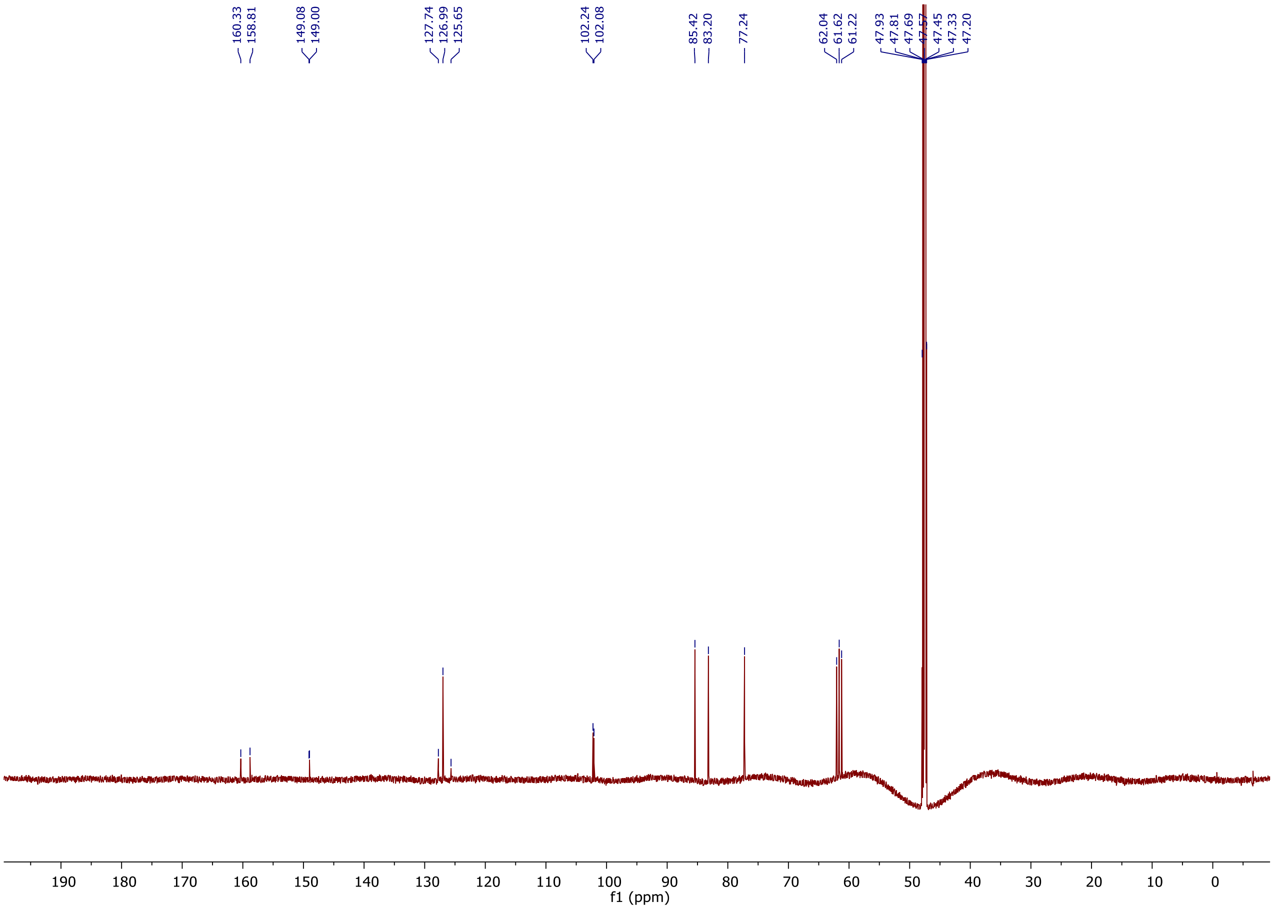
160.33
158.81
149.08
149.00
127.74
126.99
125.65
102.24
102.08
85.42
83.20
77.24
62.04
61.62
61.22
47.93
47.81
47.69
47.57
47.45
47.33
47.20
190 180 170 160 150 140 130 120 110 100 90 80 70 60 50 40 30 20 10 0
f1 (ppm)

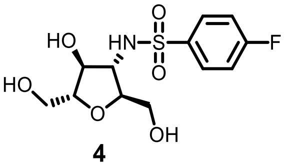
HO
HN
S
O
O
F
HO
O
OH

4

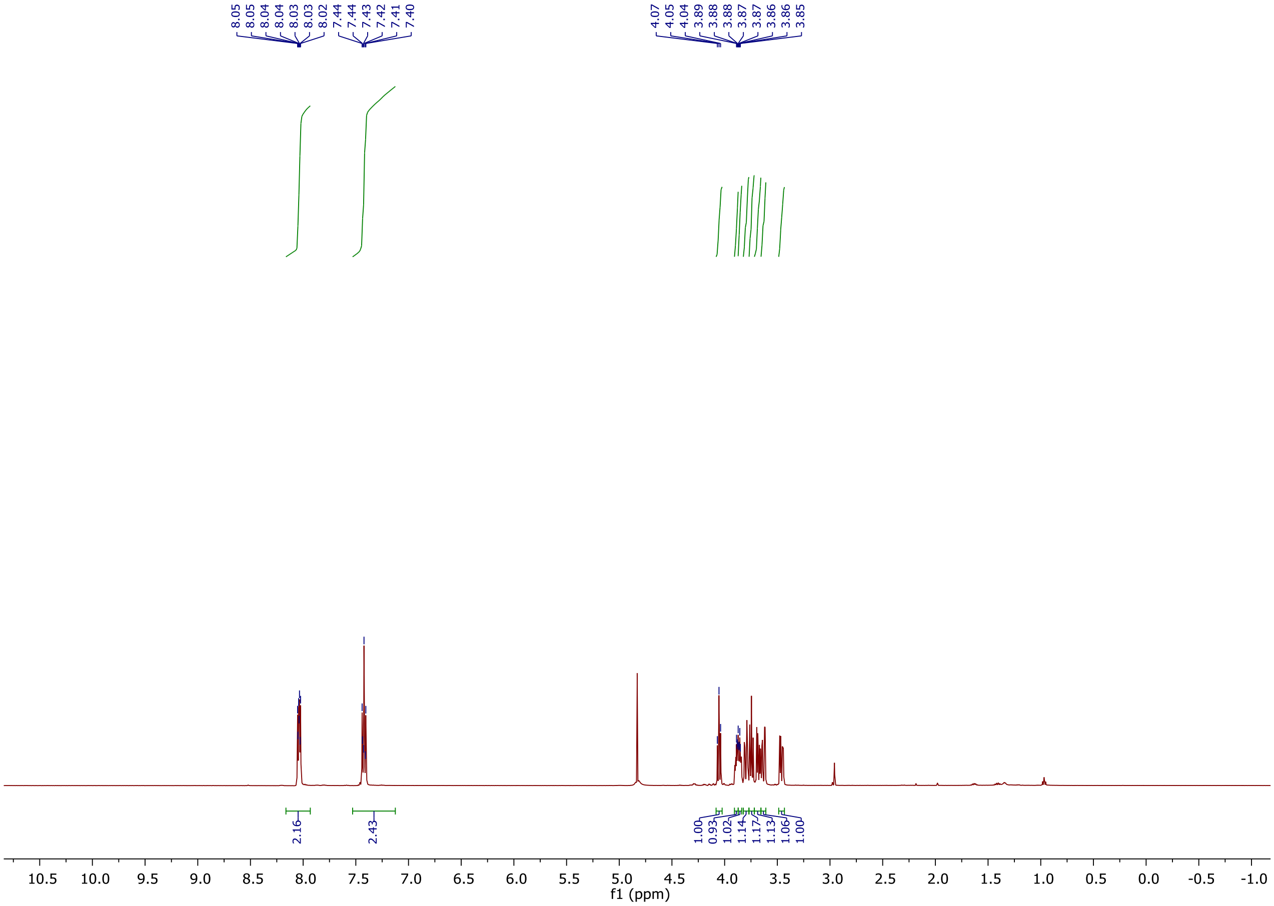
8.05
8.05
8.04
8.04
8.03
8.03
8.02
7.44
7.44
7.43
7.42
7.41
7.40
4.07
4.05
4.04
3.89
3.88
3.88
3.87
3.87
3.86
3.86
3.85
2.16
2.43
1.00
0.93
1.02
1.14
1.17
1.13
1.06
1.00
10.5 10.0 9.5 9.0 8.5 8.0 7.5 7.0 6.5 6.0 5.5 5.0 4.5 4.0 3.5 3.0 2.5 2.0 1.5 1.0 0.5 0.0 -0.5 -1.0
f1 (ppm)

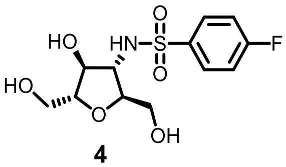

4

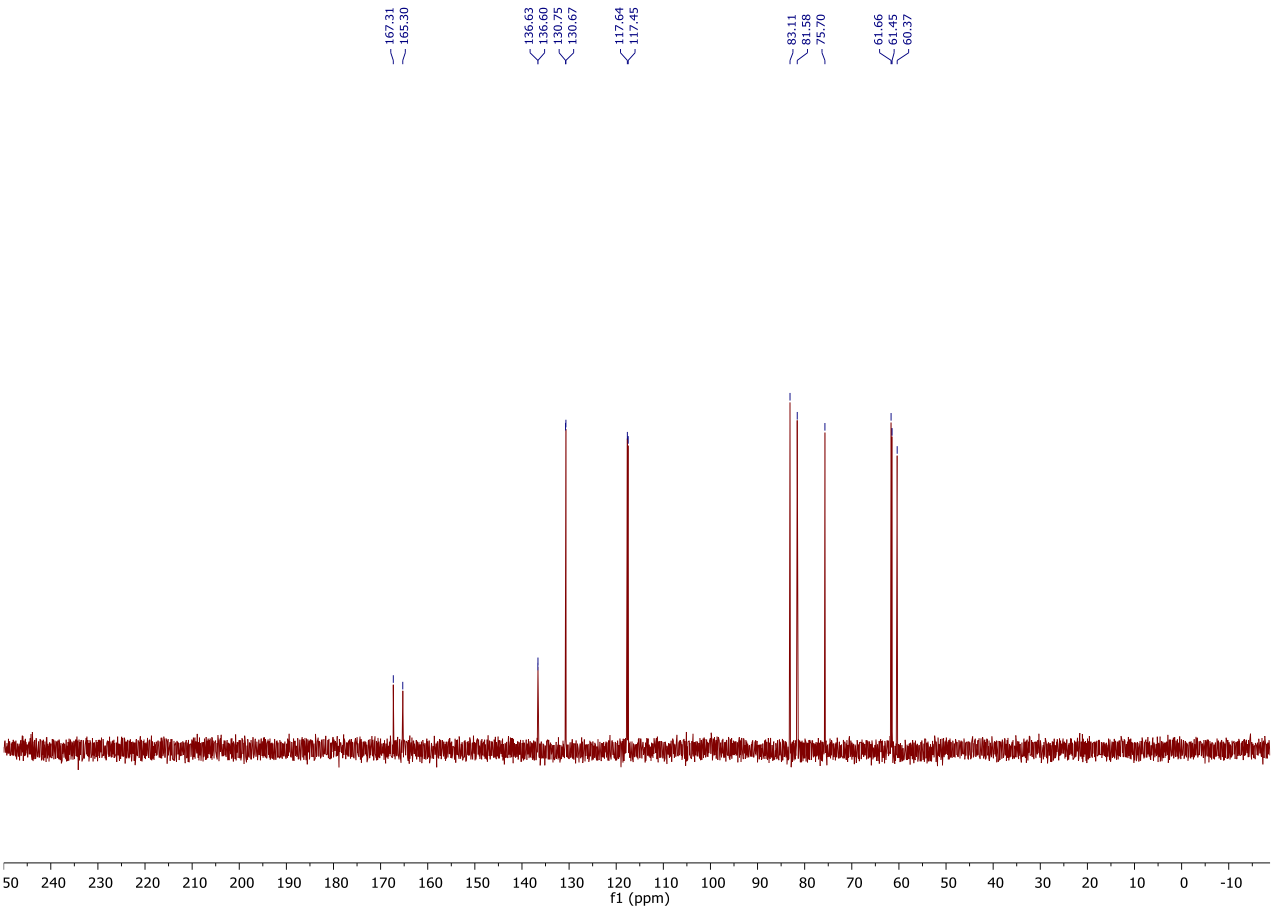
167.31
165.30
136.63
136.60
130.75
130.67
117.64
117.45
83.11
81.58
75.70
61.66
61.45
60.37
50 240 230 220 210 200 190 180 170 160 150 140 130 120 110 100 90 80 70 60 50 40 30 20 10 0 -10
f1 (ppm)

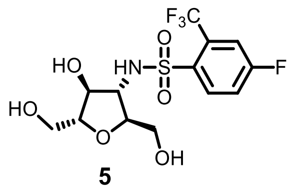

F3C
HO
HN
S
O
O
F
HO
O
OH

5

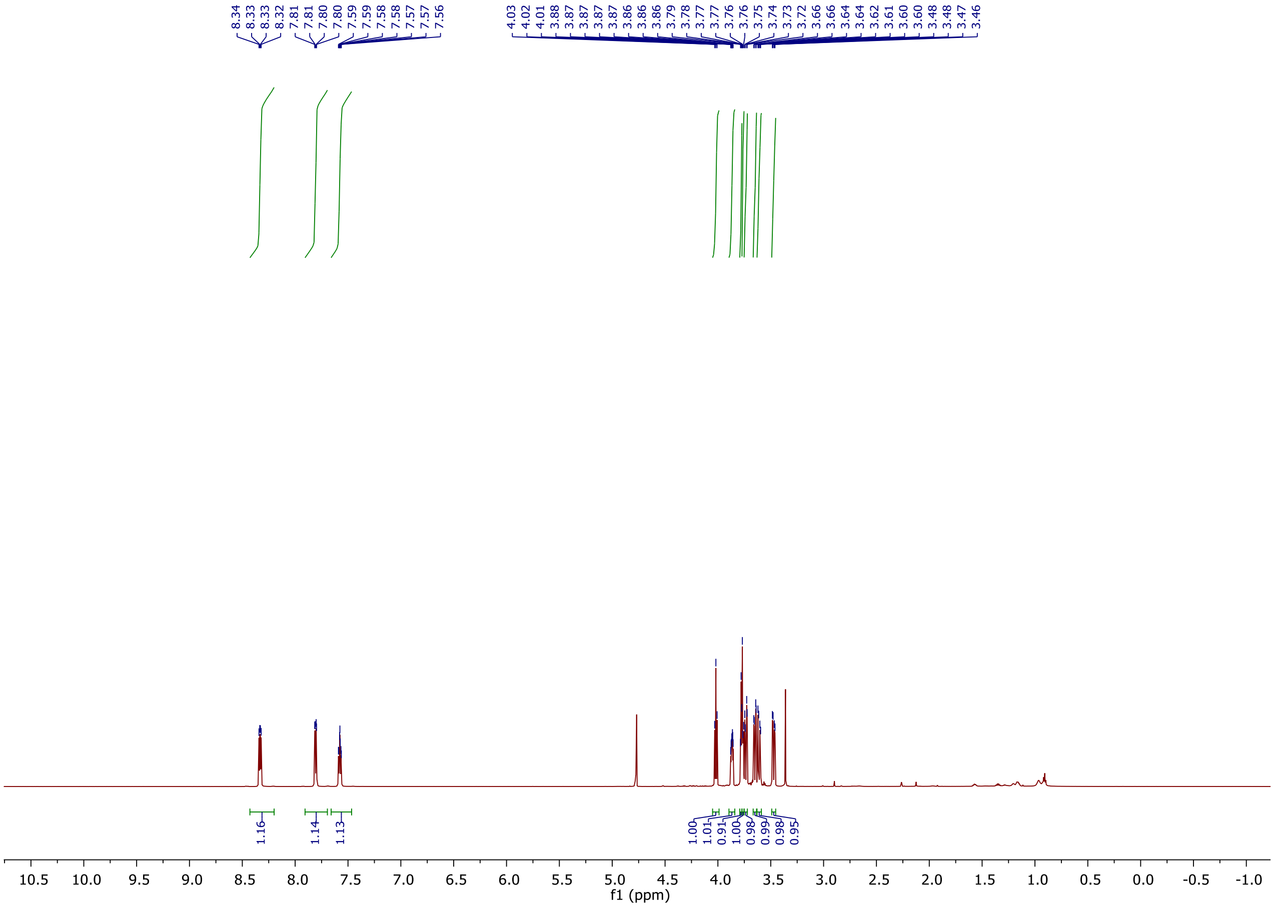

8.34
8.33
8.33
8.32
7.81
7.81
7.80
7.80
7.59
7.59
7.58
7.58
7.57
7.57
7.56
4.03
4.02
4.01
3.88
3.87
3.87
3.87
3.87
3.86
3.86
3.86
3.79
3.78
3.77
3.76
3.76
3.75
3.74
3.73
3.72
3.66
3.66
3.64
3.64
3.62
3.61
3.60
3.48
3.48
3.47
3.46
1.16
1.14
1.13
1.00
1.01
0.91
1.00
0.98
0.99
0.98
0.95
10.5
10.0
9.5
9.0
8.5
8.0
7.5
7.0
6.5
6.0
5.5
5.0
4.5
4.0
3.5
3.0
2.5
2.0
1.5
1.0
0.5
0.0
-0.5
-1.0
f1 (ppm)

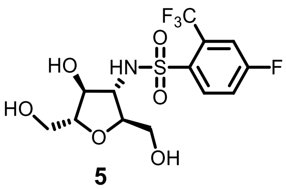
F3C
O
HO
HN
S
F
O
HO
O
OH

5

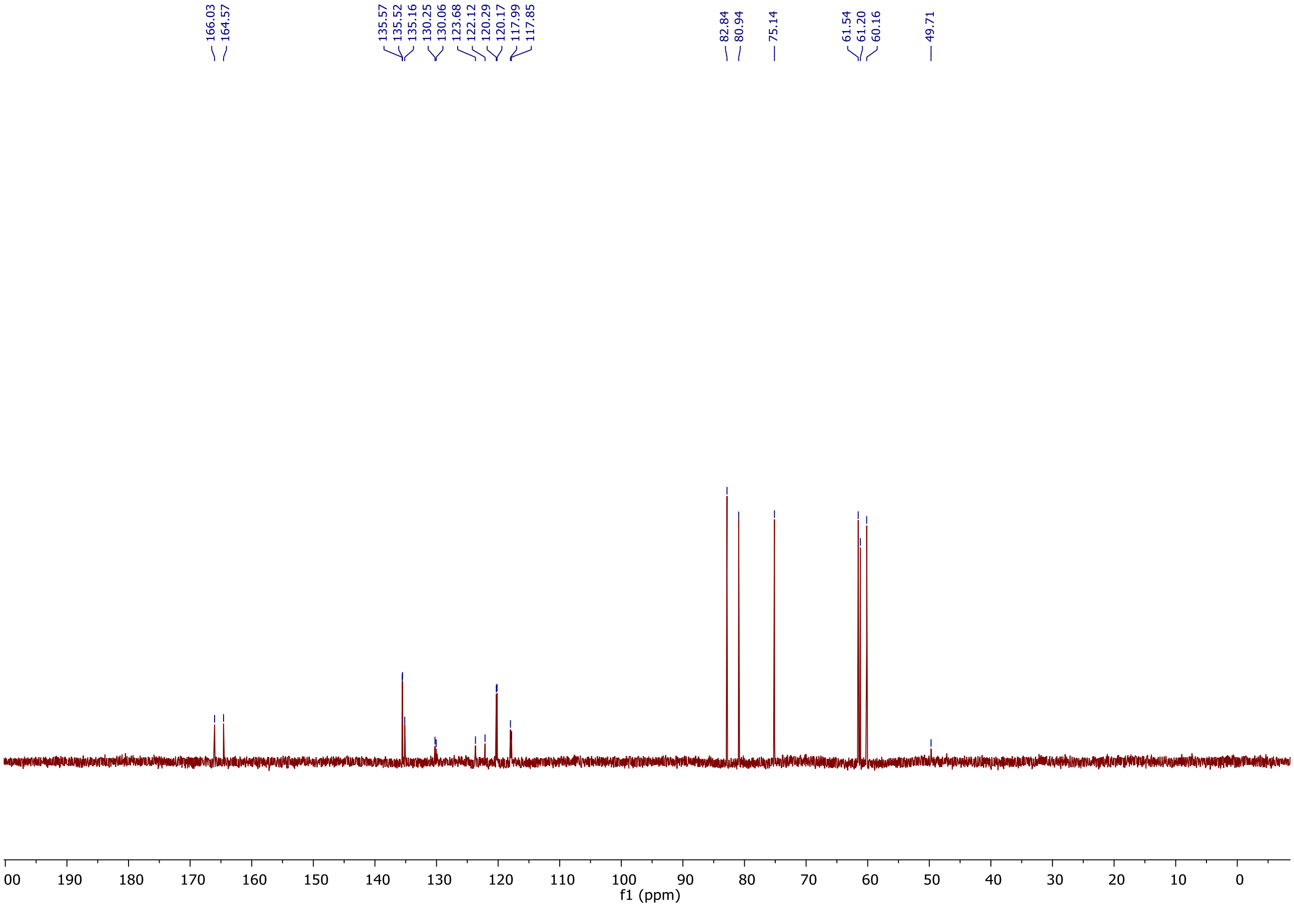
166.03
164.57
135.57
135.52
135.16
130.25
130.06
123.68
122.12
120.29
120.17
117.99
117.85
82.84
80.94
75.14
61.54
61.20
60.16
49.71
00 190 180 170 160 150 140 130 120 110 100 90 80 70 60 50 40 30 20 10 0
f1 (ppm)

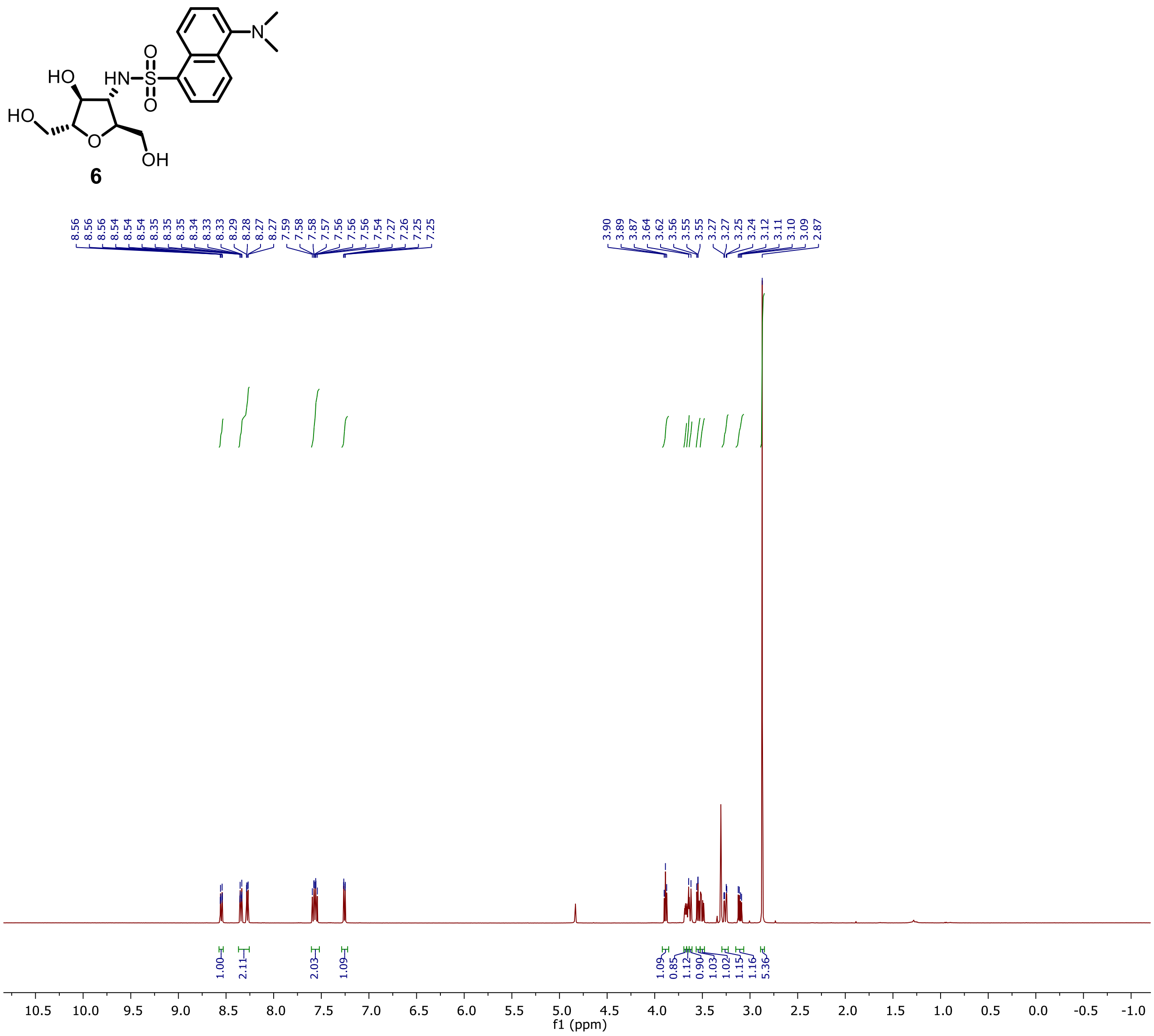

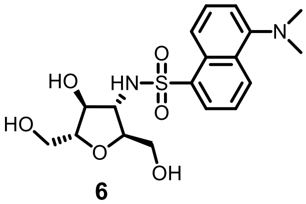

6

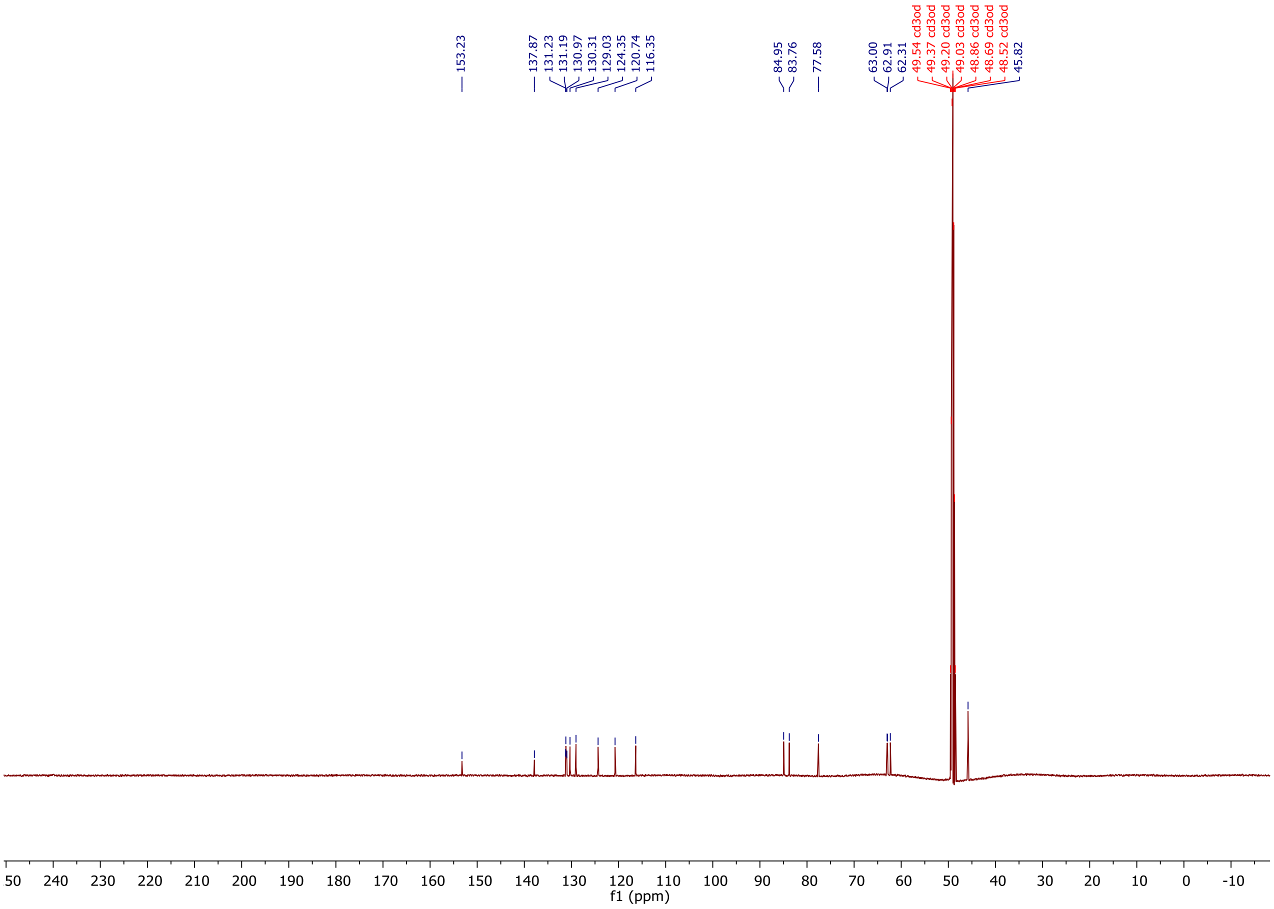
153.23
137.87
131.23
131.19
130.97
130.31
129.03
124.35
120.74
116.35
84.95
83.76
77.58
63.00
62.91
62.31
49.54 cd3od
49.37 cd3od
49.20 cd3od
49.03 cd3od
48.86 cd3od
48.69 cd3od
48.52 cd3od
45.82
50 240 230 220 210 200 190 180 170 160 150 140 130 120 110 100 90 80 70 60 50 40 30 20 10 0 -10
f1 (ppm)

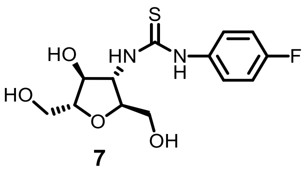

7

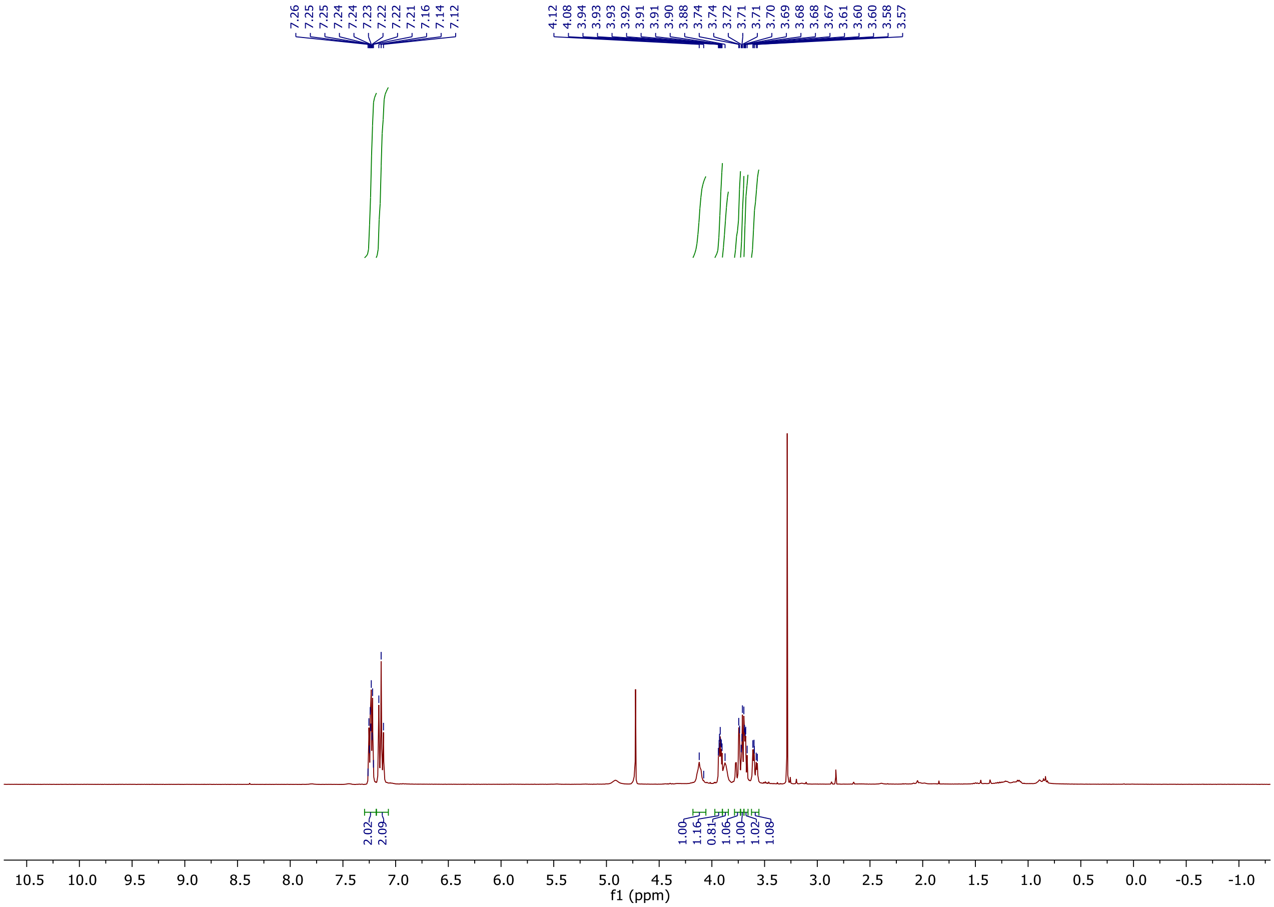
7.26
7.25
7.25
7.24
7.24
7.23
7.22
7.22
7.21
7.16
7.14
7.12
4.12
4.08
3.94
3.93
3.93
3.92
3.91
3.91
3.90
3.88
3.74
3.74
3.72
3.71
3.71
3.70
3.69
3.68
3.68
3.67
3.61
3.60
3.60
3.58
3.57
2.02
2.09
1.00
1.16
0.81
1.06
1.00
1.02
1.08
10.5 10.0 9.5 9.0 8.5 8.0 7.5 7.0 6.5 6.0 5.5 5.0 4.5 4.0 3.5 3.0 2.5 2.0 1.5 1.0 0.5 0.0 -0.5 -1.0
f1 (ppm)

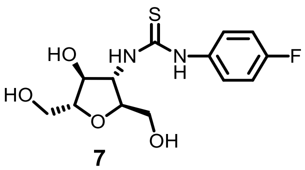
S
HO
HN
N
H
F
HO
O
OH

7

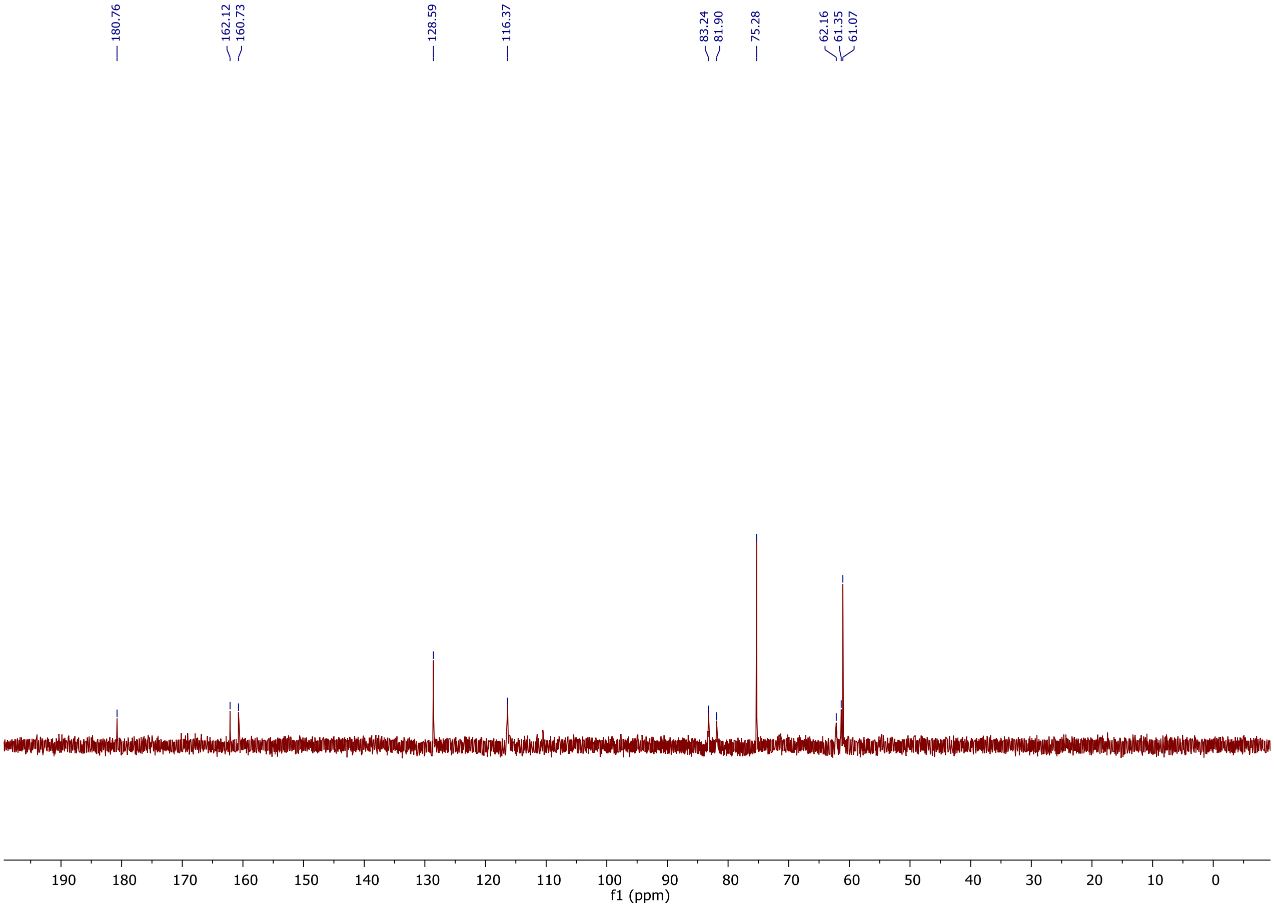
180.76
162.12
160.73
128.59
116.37
83.24
81.90
75.28
62.16
61.35
61.07
190 180 170 160 150 140 130 120 110 100 90 80 70 60 50 40 30 20 10 0
f1 (ppm)

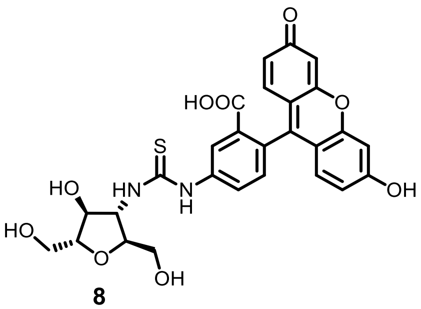
O
HOOC
O
S
HO
HN
N
H
OH
HO
O
OH

8

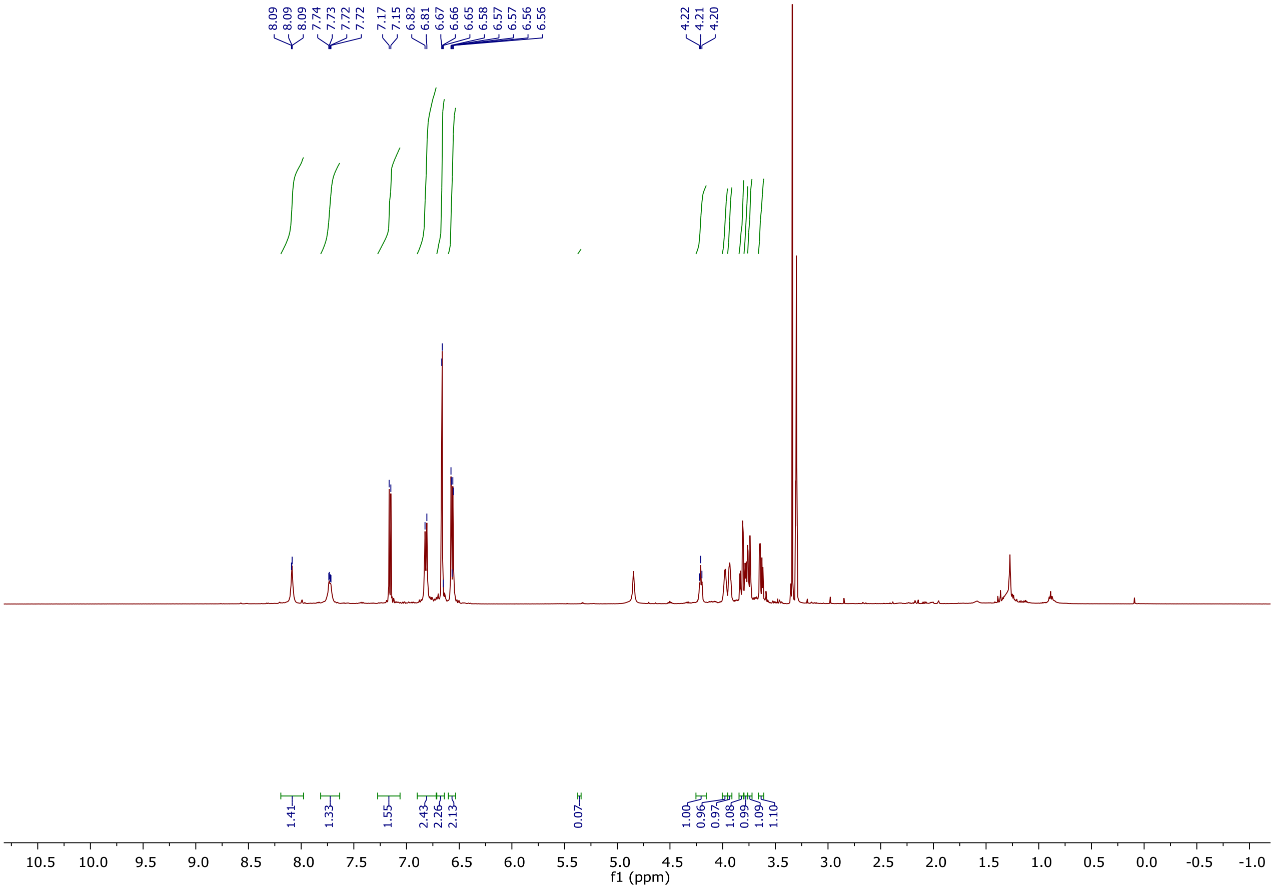
8.09
8.09
8.09
7.74
7.73
7.72
7.72
7.17
7.15
6.82
6.81
6.67
6.66
6.65
6.58
6.57
6.57
6.56
6.56
4.22
4.21
4.20
1.41
1.33
1.55
2.43
2.26
2.13
0.07
1.00
0.96
0.97
1.08
0.99
1.09
1.10
10.5
10.0
9.5
9.0
8.5
8.0
7.5
7.0
6.5
6.0
5.5
5.0
4.5
4.0
3.5
3.0
2.5
2.0
1.5
1.0
0.5
0.0
-0.5
-1.0
f1 (ppm)

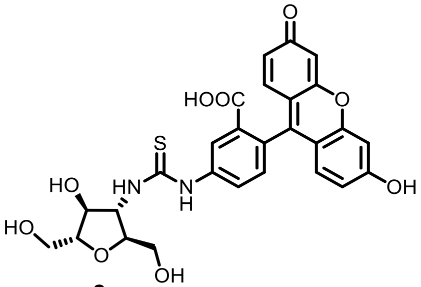
O
HOOC
O
S
HO
HN
N
H
OH
HO
O
OH

8

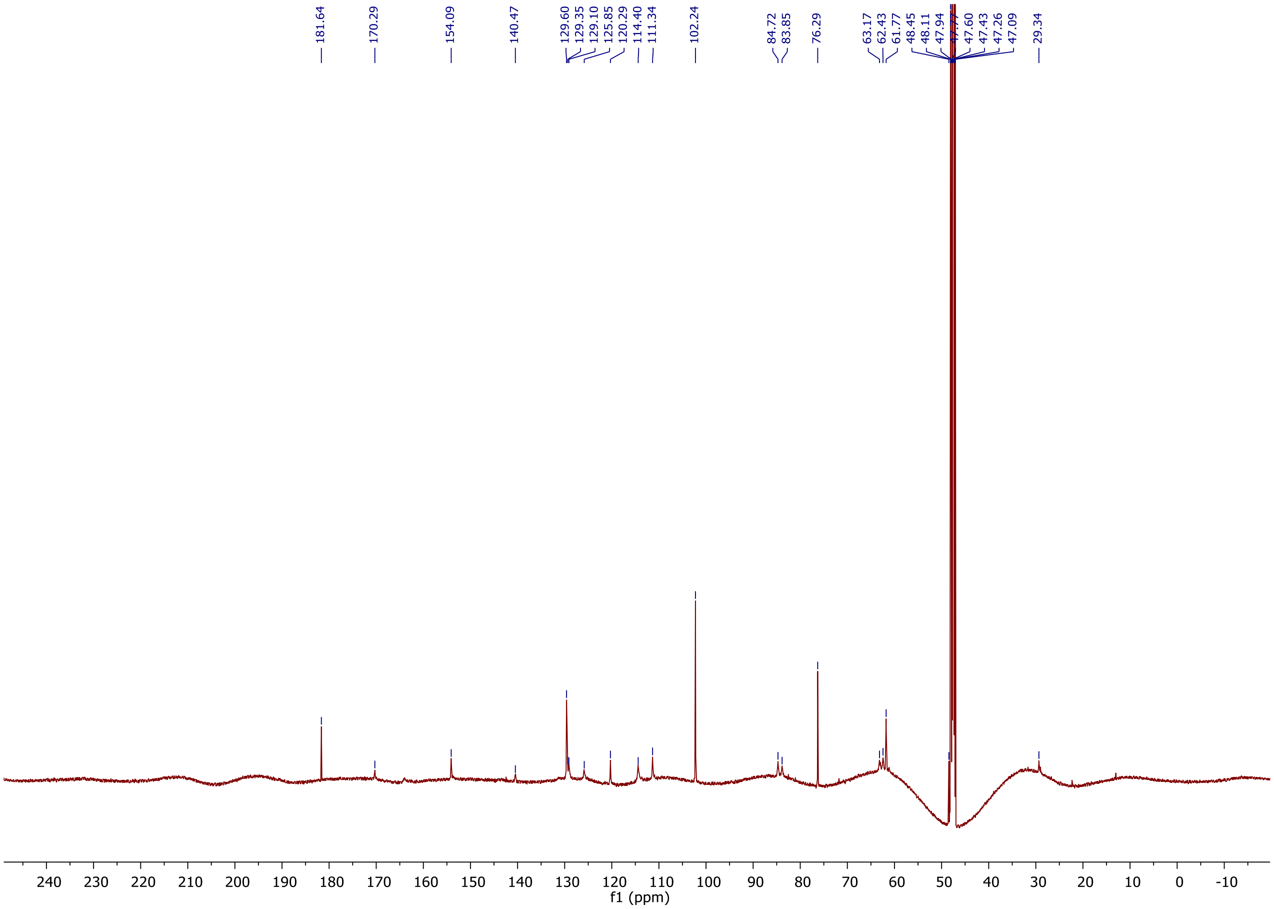
181.64
170.29
154.09
140.47
129.60
129.35
129.10
125.85
120.29
114.40
111.34
102.24
84.72
83.85
76.29
63.17
62.43
61.77
48.45
48.11
47.94
47.77
47.60
47.43
47.26
47.09
29.34
240 230 220 210 200 190 180 170 160 150 140 130 120 110 100 90 80 70 60 50 40 30 20 10 0 -10
f1 (ppm)

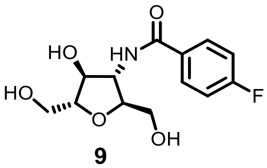

9

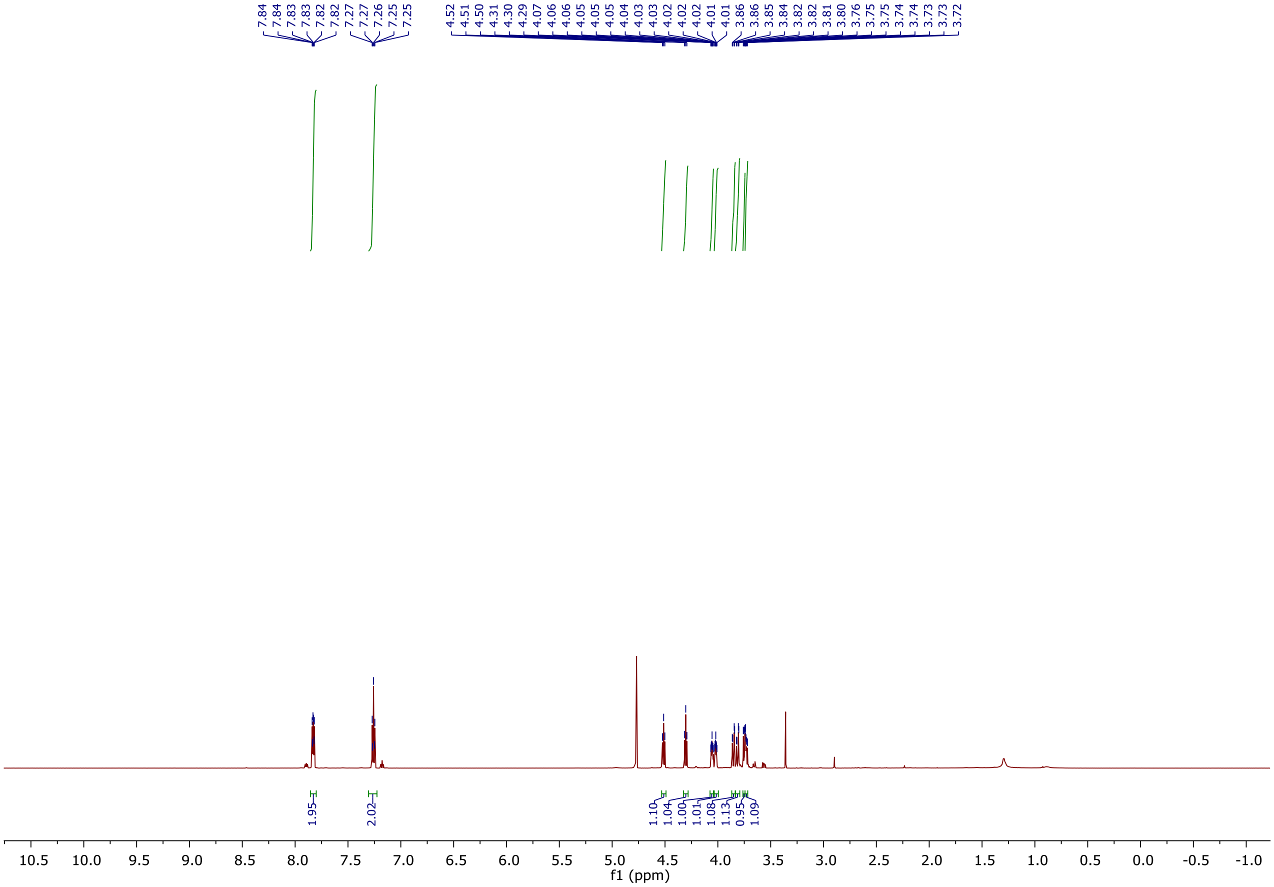

7.84
7.84
7.83
7.82
7.82
7.27
7.27
7.26
7.25
7.25
4.52
4.51
4.50
4.31
4.30
4.29
4.07
4.06
4.05
4.05
4.05
4.04
4.03
4.03
4.02
4.02
4.02
4.01
4.01
3.86
3.86
3.85
3.84
3.82
3.82
3.81
3.80
3.76
3.75
3.75
3.74
3.74
3.73
3.73
3.72
1.95
2.02
1.10
1.04
1.00
1.01
1.08
1.13
0.95
1.09
10.5 10.0 9.5 9.0 8.5 8.0 7.5 7.0 6.5 6.0 5.5 5.0 4.5 4.0 3.5 3.0 2.5 2.0 1.5 1.0 0.5 0.0 -0.5 -1.0
f1 (ppm)

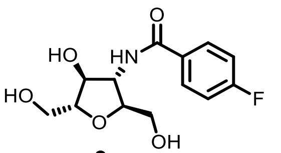
O
HO
HN
HO
F
O
OH

9

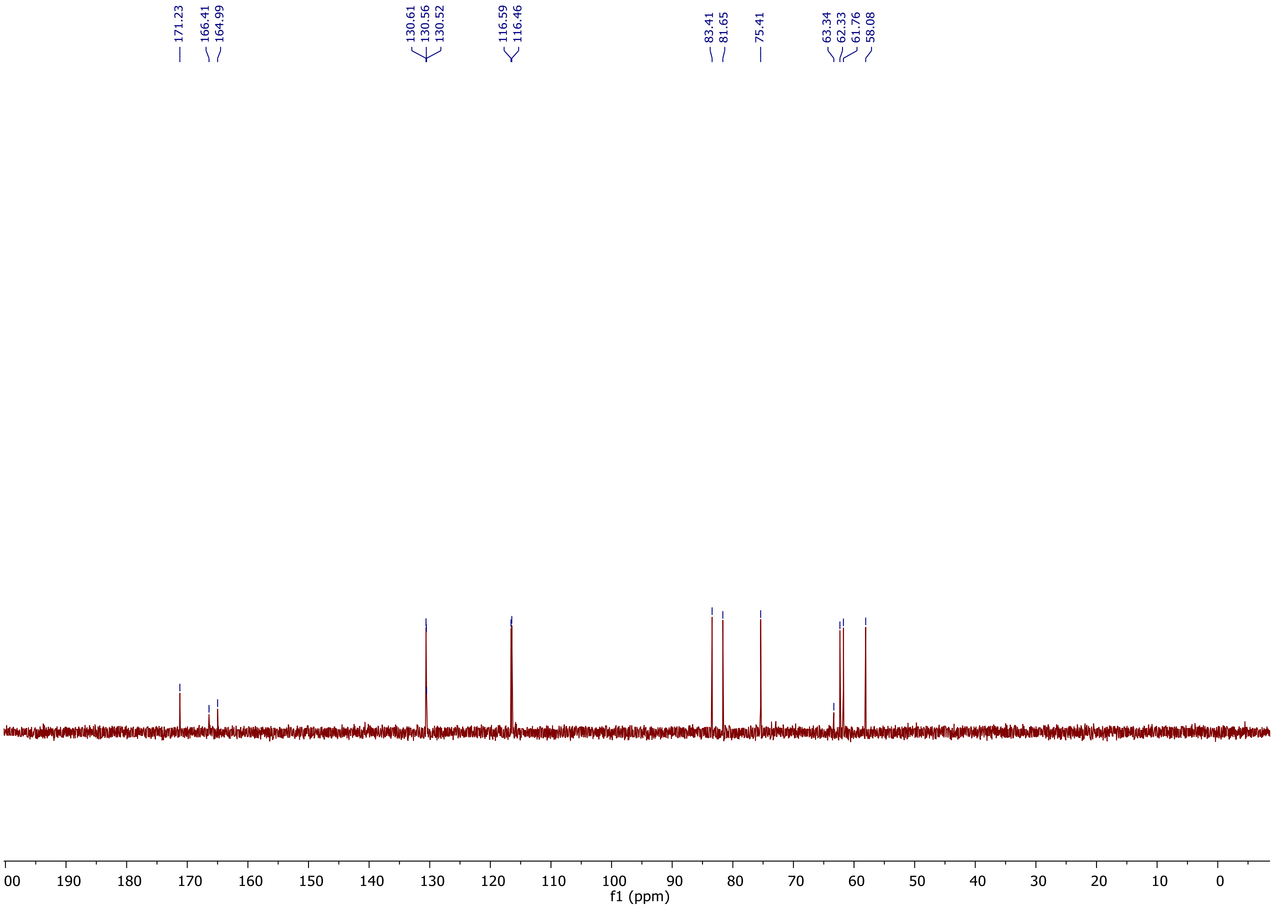
171.23
166.41
164.99
130.61
130.56
130.52
116.59
116.46
83.41
81.65
75.41
63.34
62.33
61.76
58.08
00 190 180 170 160 150 140 130 120 110 100 90 80 70 60 50 40 30 20 10 0
f1 (ppm)

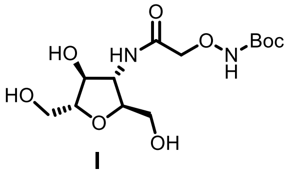
O
HN
O
N
Boc
H
HO
HO
O
OH

I

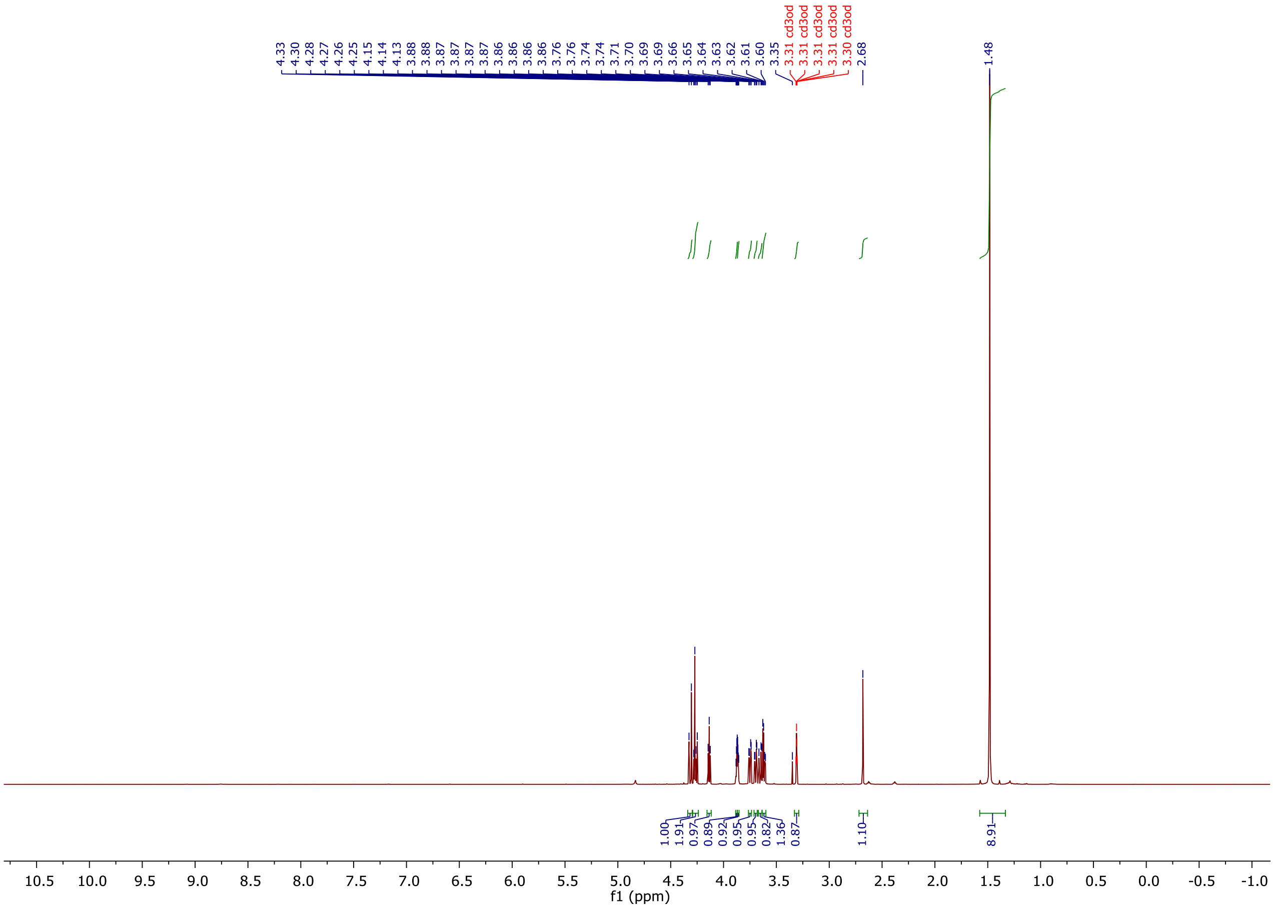
4.33
4.30
4.28
4.27
4.26
4.25
4.15
4.14
4.13
3.88
3.88
3.87
3.87
3.87
3.86
3.86
3.86
3.76
3.76
3.74
3.74
3.71
3.70
3.69
3.69
3.66
3.65
3.64
3.63
3.62
3.61
3.60
3.35
3.31 cd3od
3.31 cd3od
3.31 cd3od
3.31 cd3od
3.30 cd3od
2.68
1.48
1.00
1.91
0.97
0.89
0.92
0.95
0.95
0.82
1.36
0.87
1.10
8.91
10.5
10.0
9.5
9.0
8.5
8.0
7.5
7.0
6.5
6.0
5.5
5.0
4.5
4.0
3.5
3.0
2.5
2.0
1.5
1.0
0.5
0.0
-0.5
-1.0
f1 (ppm)

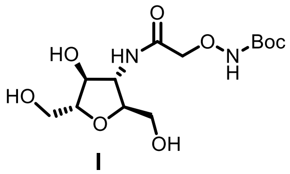

I

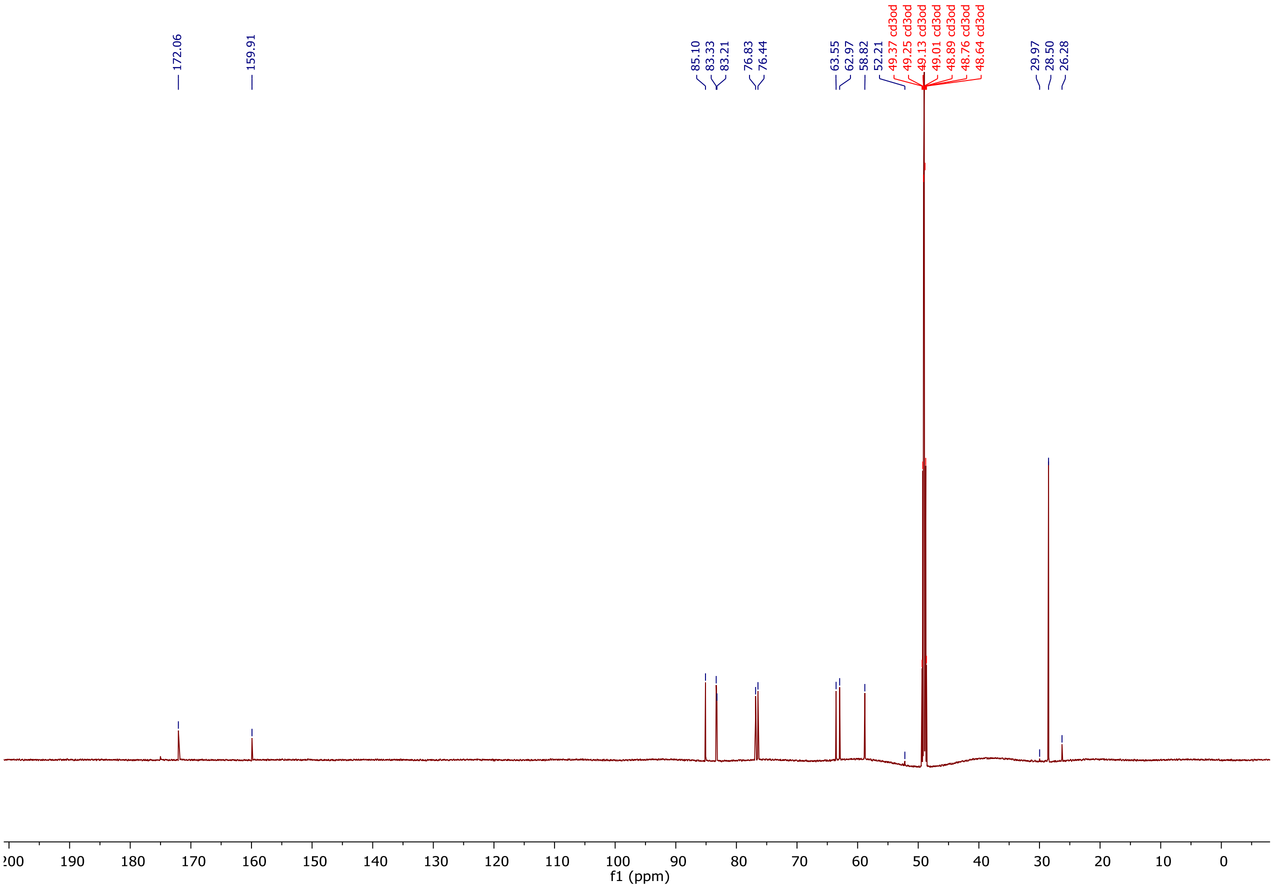
172.06
159.91
85.10
83.33
83.21
76.83
76.44
63.55
62.97
58.82
52.21
49.37 cd3od
49.25 cd3od
49.13 cd3od
49.01 cd3od
48.89 cd3od
48.76 cd3od
48.64 cd3od
29.97
28.50
26.28
200 190 180 170 160 150 140 130 120 110 100 90 80 70 60 50 40 30 20 10 0
f1 (ppm)

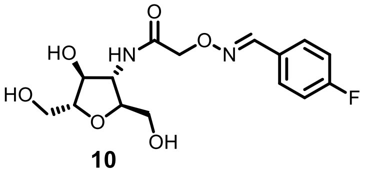

HO
HN
O
O
N
F
HO
O
OH

10

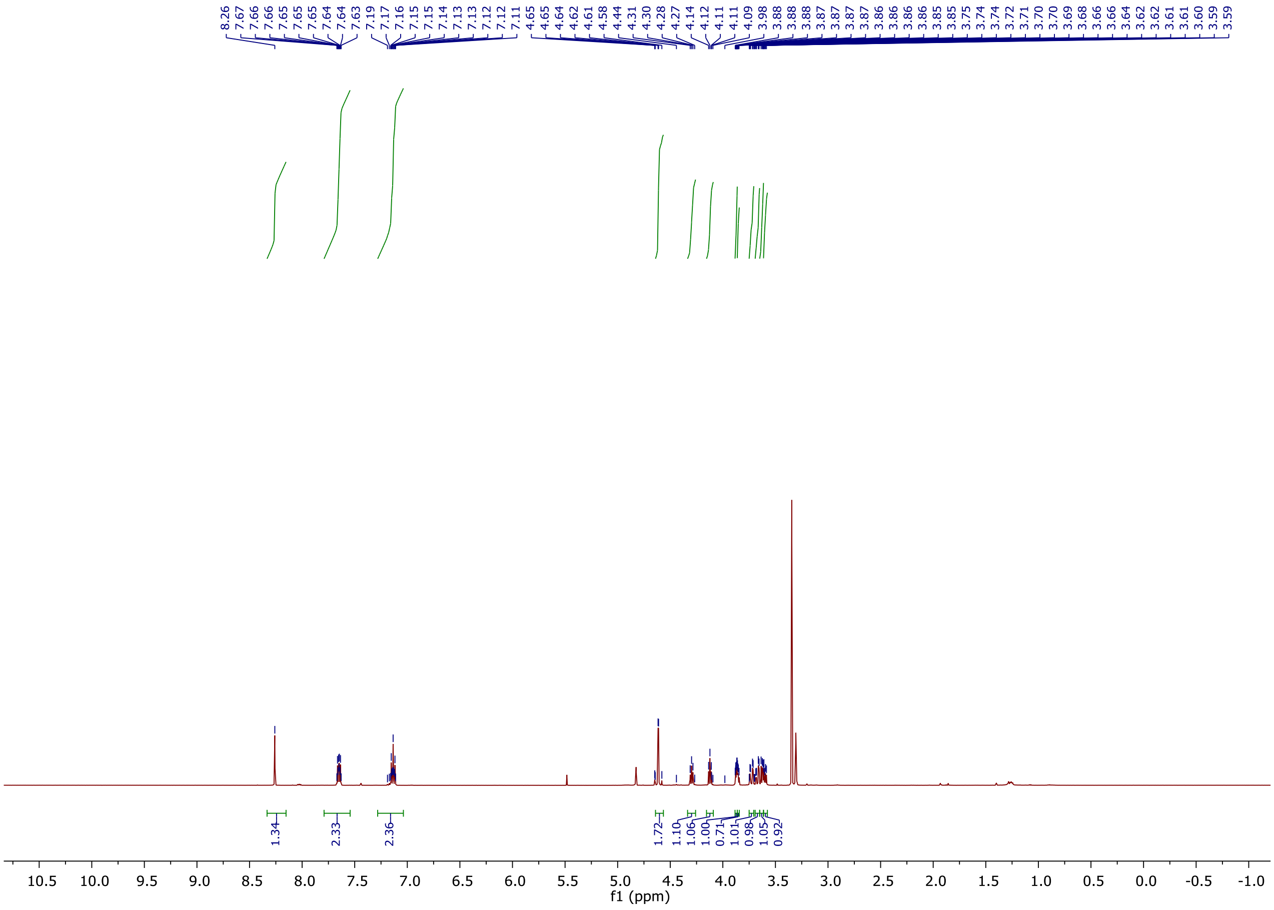

8.26 7.67 7.66 7.65 7.65 7.65 7.64 7.64 7.63 7.19 7.17 7.16 7.15 7.14 7.13 7.13 7.12 7.12 7.11 4.65 4.64 4.62 4.61 4.58 4.44 4.31 4.30 4.28 4.27 4.14 4.12 4.11 4.11 4.09 3.98 3.88 3.88 3.87 3.87 3.87 3.86 3.86 3.86 3.86 3.85 3.85 3.75 3.74 3.74 3.72 3.71 3.70 3.69 3.68 3.66 3.66 3.64 3.62 3.61 3.60 3.59 3.59
1.34 2.33 2.36 1.72 1.10 1.06 1.00 0.71 1.01 0.98 1.05 0.92
10.5 10.0 9.5 9.0 8.5 8.0 7.5 7.0 6.5 6.0 5.5 5.0 4.5 4.0 3.5 3.0 2.5 2.0 1.5 1.0 0.5 0.0 -0.5 -1.0
f1 (ppm)

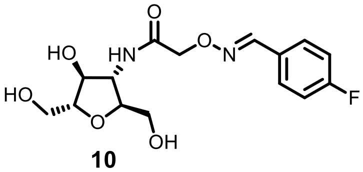

10

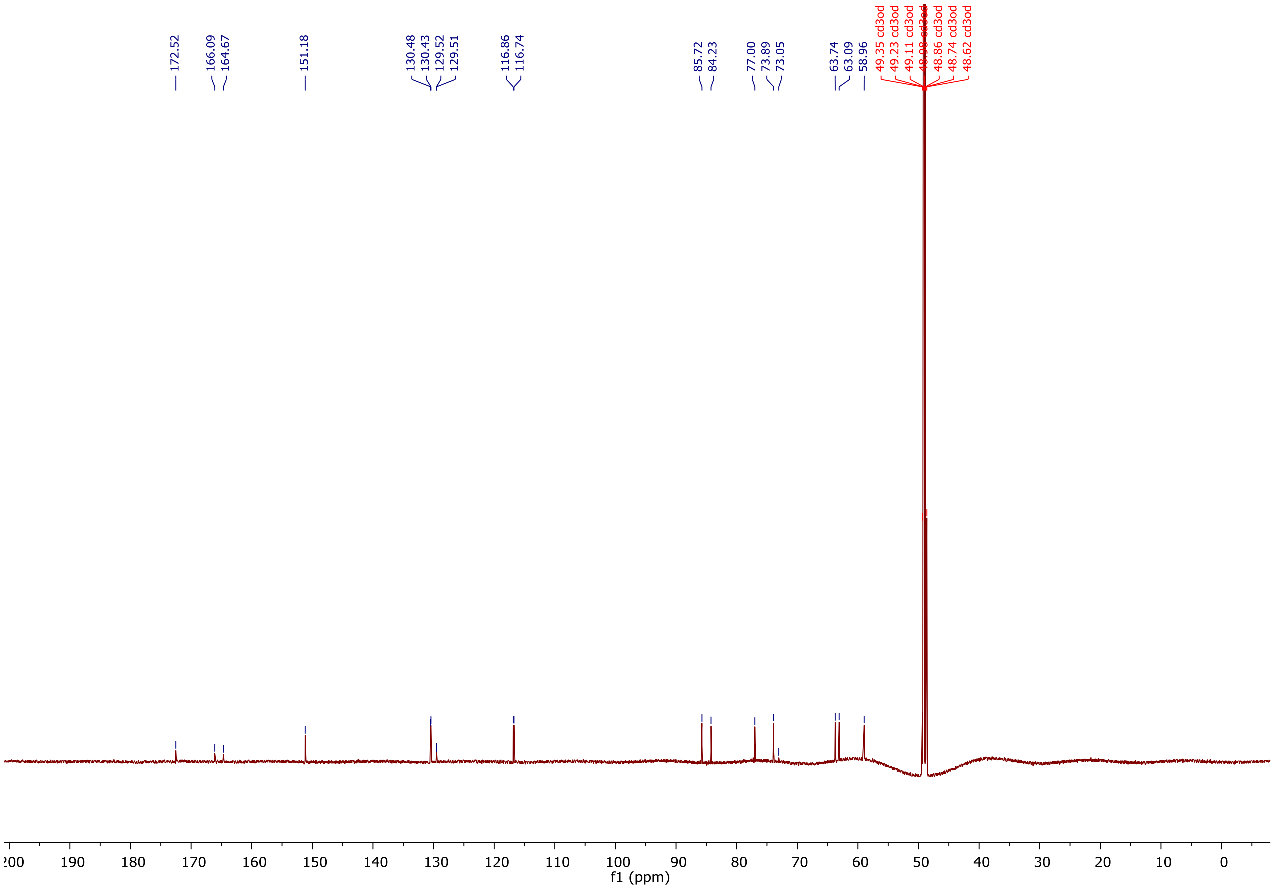
172.52
166.09
164.67
151.18
130.48
130.43
129.52
129.51
116.86
116.74
85.72
84.23
77.00
73.89
73.05
63.74
63.09
58.96
49.35 cd3od
49.23 cd3od
49.11 cd3od
48.86 cd3od
48.74 cd3od
48.62 cd3od
f1 (ppm)

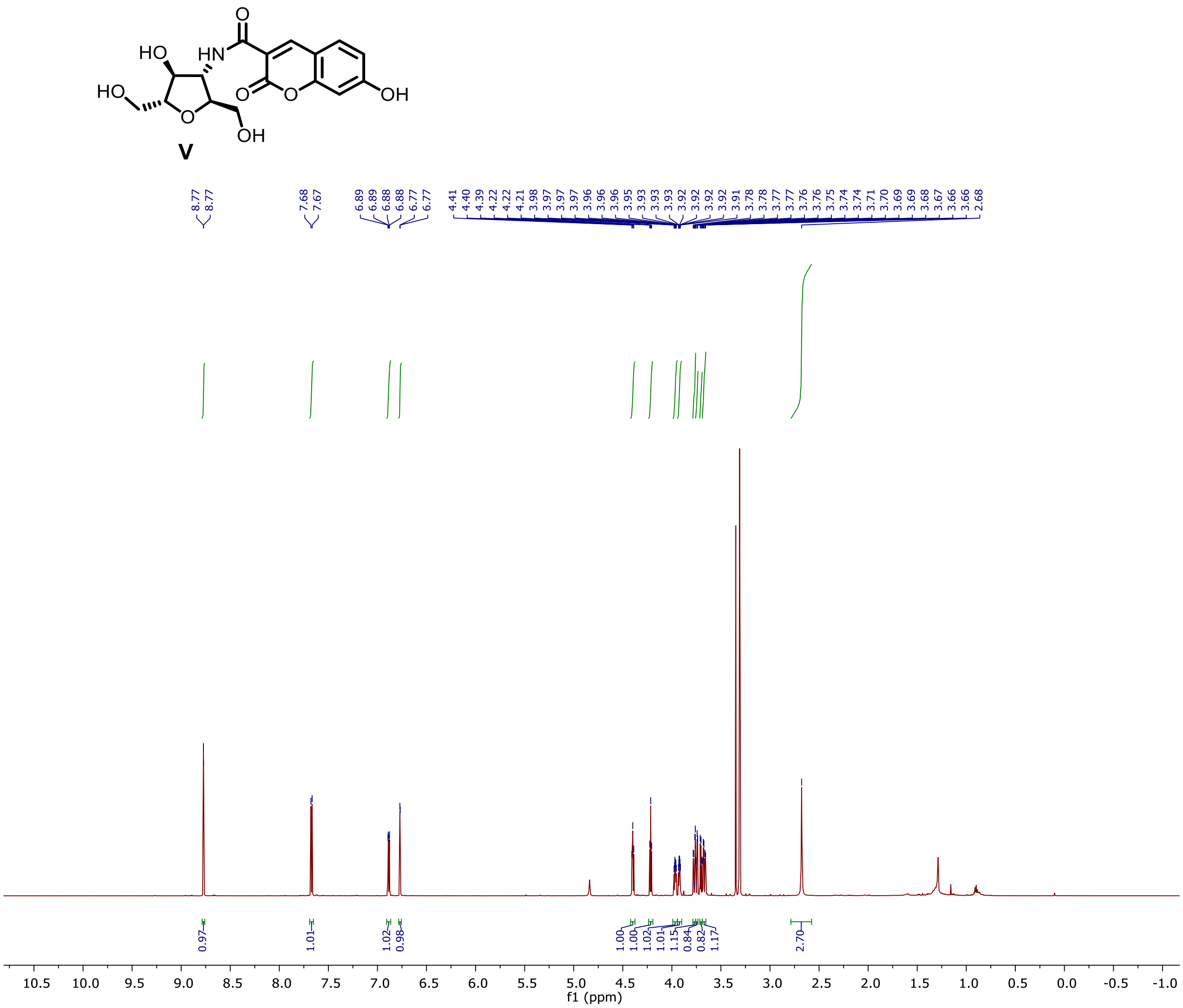
HO
HN
O
HO
O
O
O
OH
OH
V
8.77
8.77
7.68
7.67
6.89
6.89
6.88
6.88
6.77
6.77
4.41
4.40
4.39
4.22
4.22
4.21
3.98
3.97
3.97
3.97
3.96
3.96
3.96
3.95
3.93
3.93
3.93
3.92
3.92
3.92
3.92
3.91
3.78
3.78
3.77
3.77
3.76
3.76
3.75
3.74
3.74
3.71
3.70
3.69
3.69
3.68
3.67
3.66
2.68
0.97
1.01
1.02
0.98
1.00
1.00
1.02
1.01
1.15
0.84
0.82
1.17
2.70
10.5
10.0
9.5
9.0
8.5
8.0
7.5
7.0
6.5
6.0
5.5
5.0
4.5
4.0
3.5
3.0
2.5
2.0
1.5
1.0
0.5
0.0
-0.5
-1.0
f1 (ppm)

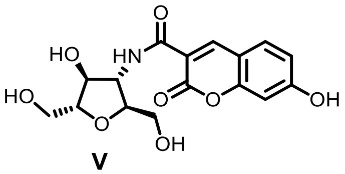

V

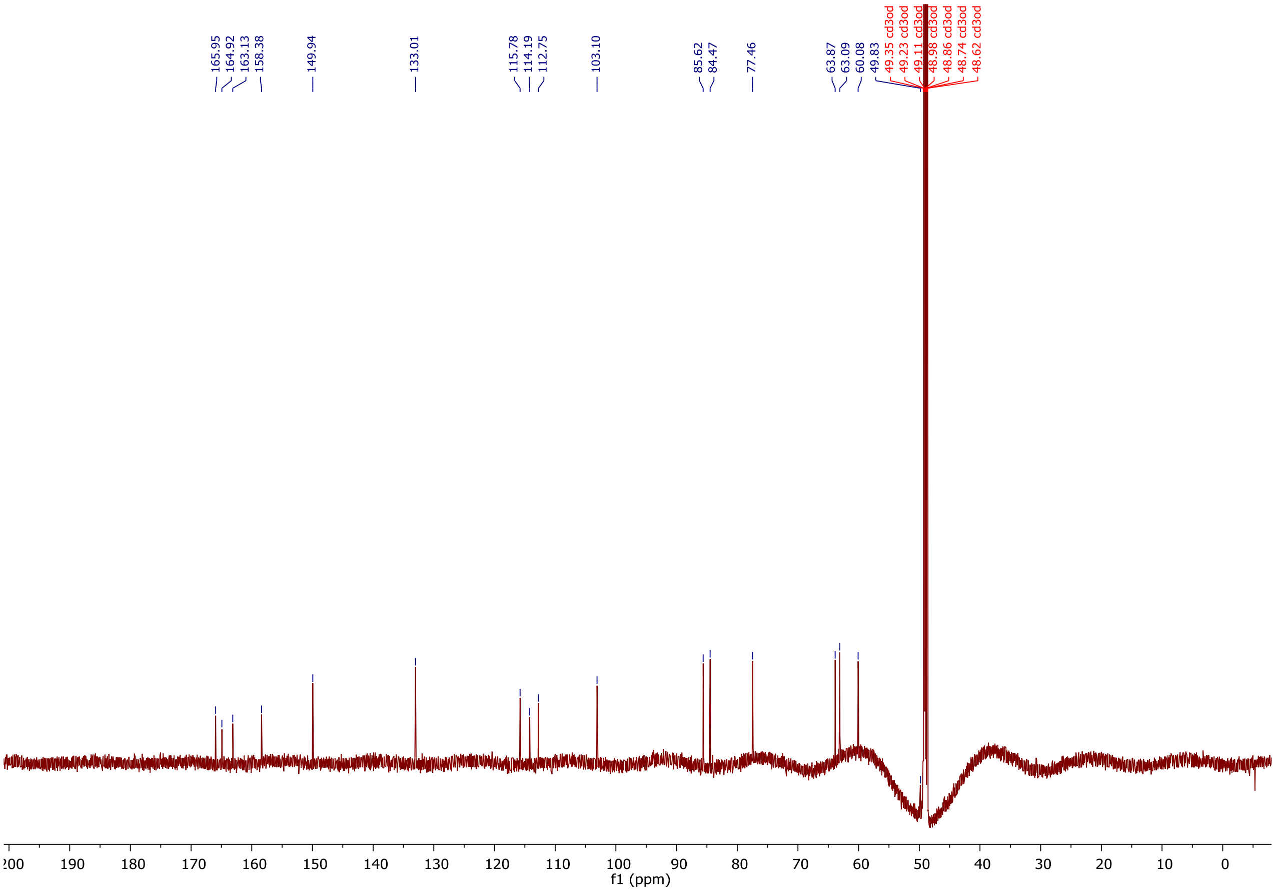
165.95
164.92
163.13
158.38
149.94
133.01
115.78
114.19
112.75
103.10
85.62
84.47
77.46
63.87
63.09
60.08
49.83
49.35 cd3od
49.23 cd3od
49.11 cd3od
48.98 cd3od
48.86 cd3od
48.74 cd3od
48.62 cd3od
200 190 180 170 160 150 140 130 120 110 100 90 80 70 60 50 40 30 20 10 0
f1 (ppm)

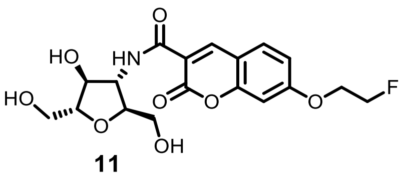

11

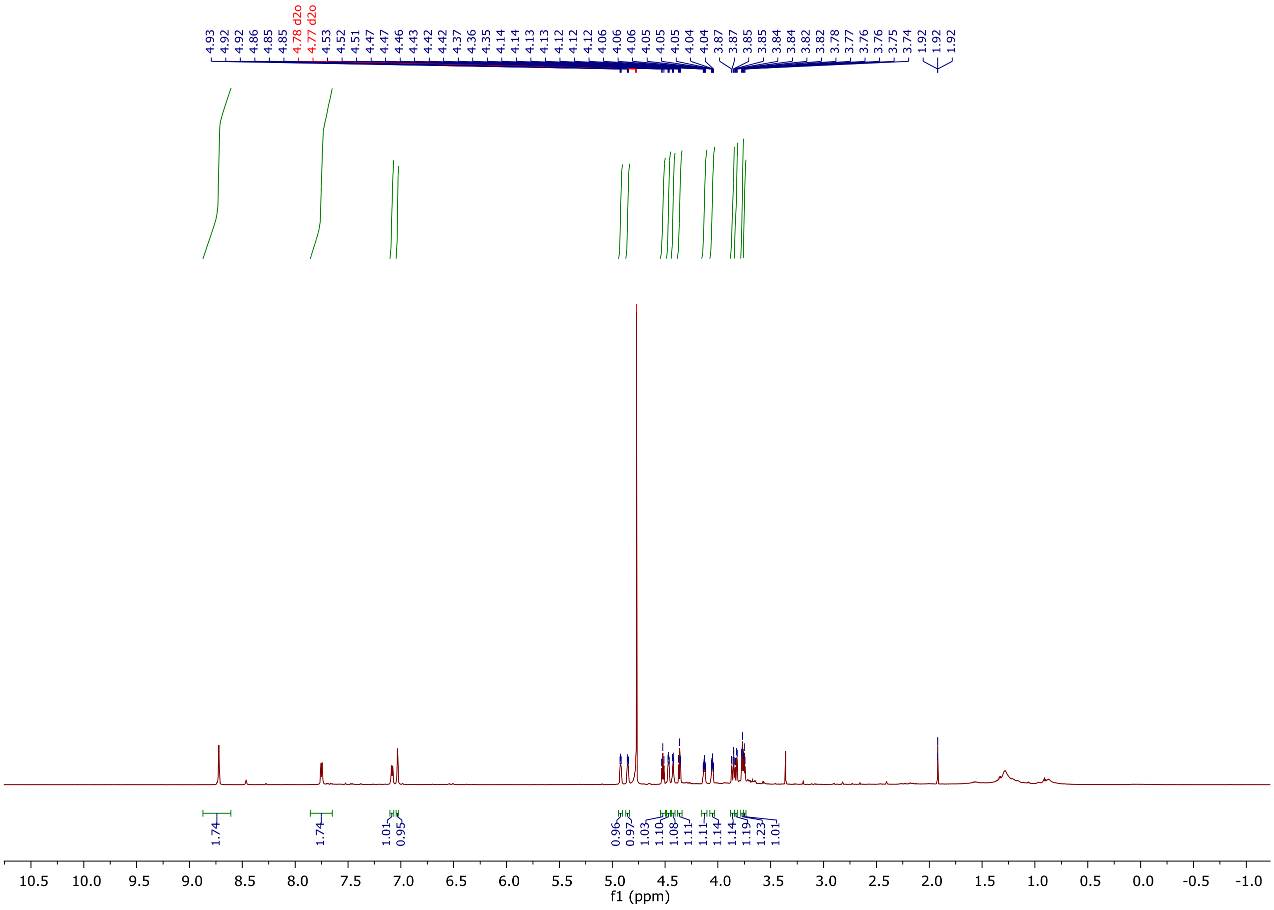

4.93 4.92 4.92 4.86 4.85 4.85 4.78 d2o 4.77 d2o 4.53 4.52 4.51 4.47 4.47 4.46 4.43 4.42 4.42 4.37 4.36 4.35 4.14 4.14 4.13 4.13 4.12 4.12 4.12 4.12 4.06 4.06 4.06 4.05 4.05 4.05 4.04 4.04 3.87 3.87 3.85 3.85 3.84 3.84 3.82 3.82 3.78 3.77 3.76 3.76 3.75 3.74 1.92 1.92 1.92
1.74 1.74 1.01 0.95 0.96 0.97 1.03 1.10 1.08 1.11 1.11 1.14 1.14 1.19 1.23 1.01
10.5 10.0 9.5 9.0 8.5 8.0 7.5 7.0 6.5 6.0 5.5 5.0 4.5 4.0 3.5 3.0 2.5 2.0 1.5 1.0 0.5 0.0 -0.5 -1.0
f1 (ppm)

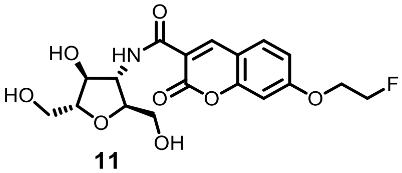

11

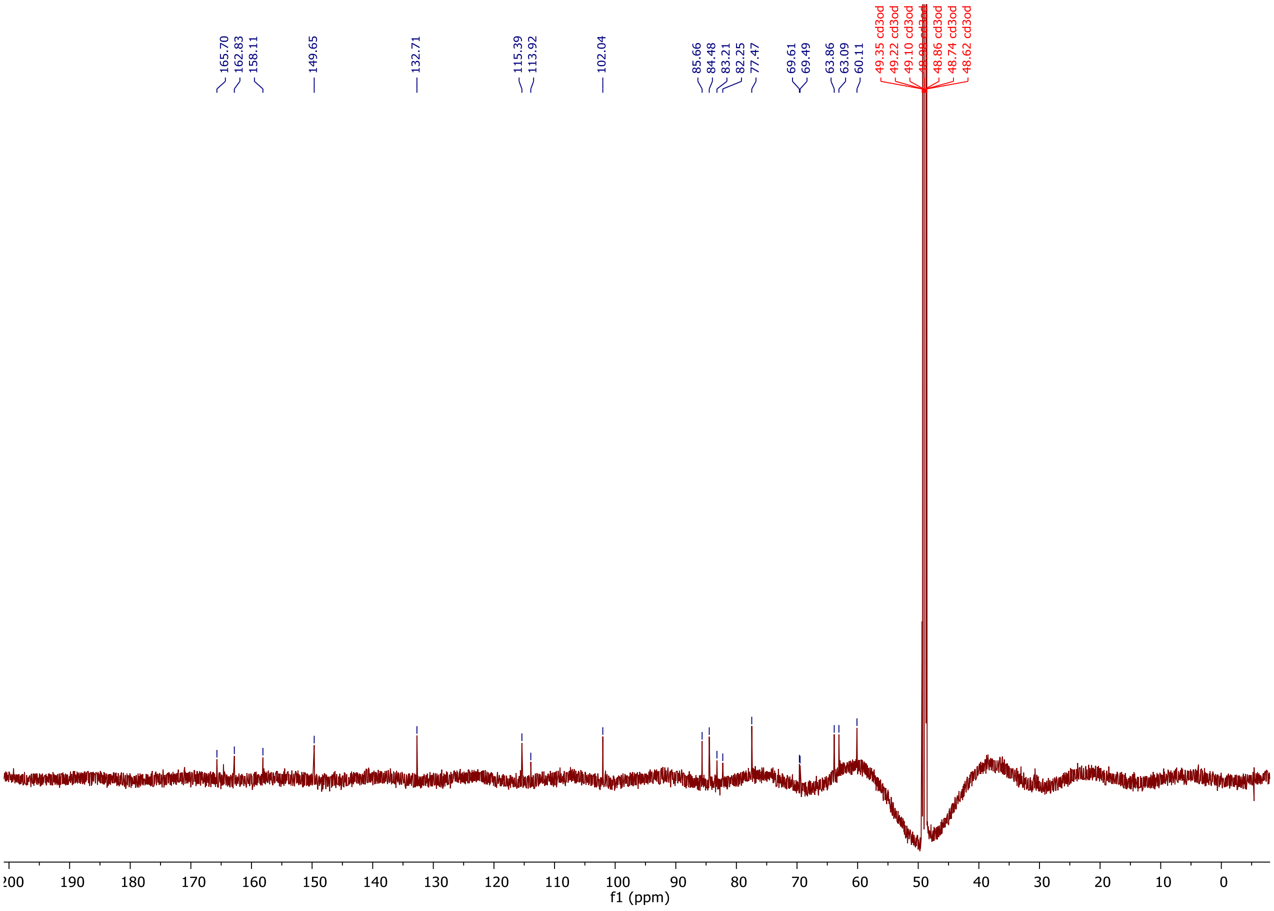

165.70
162.83
158.11
149.65
132.71
115.39
113.92
102.04
85.66
84.48
83.21
82.25
77.47
69.61
69.49
63.86
63.09
60.11
49.35 cd3od
49.22 cd3od
49.10 cd3od
48.86 cd3od
48.74 cd3od
48.62 cd3od
200
190
180
170
160
150
140
130
120
110
100
90
80
70
60
50
40
30
20
10
0
f1 (ppm)

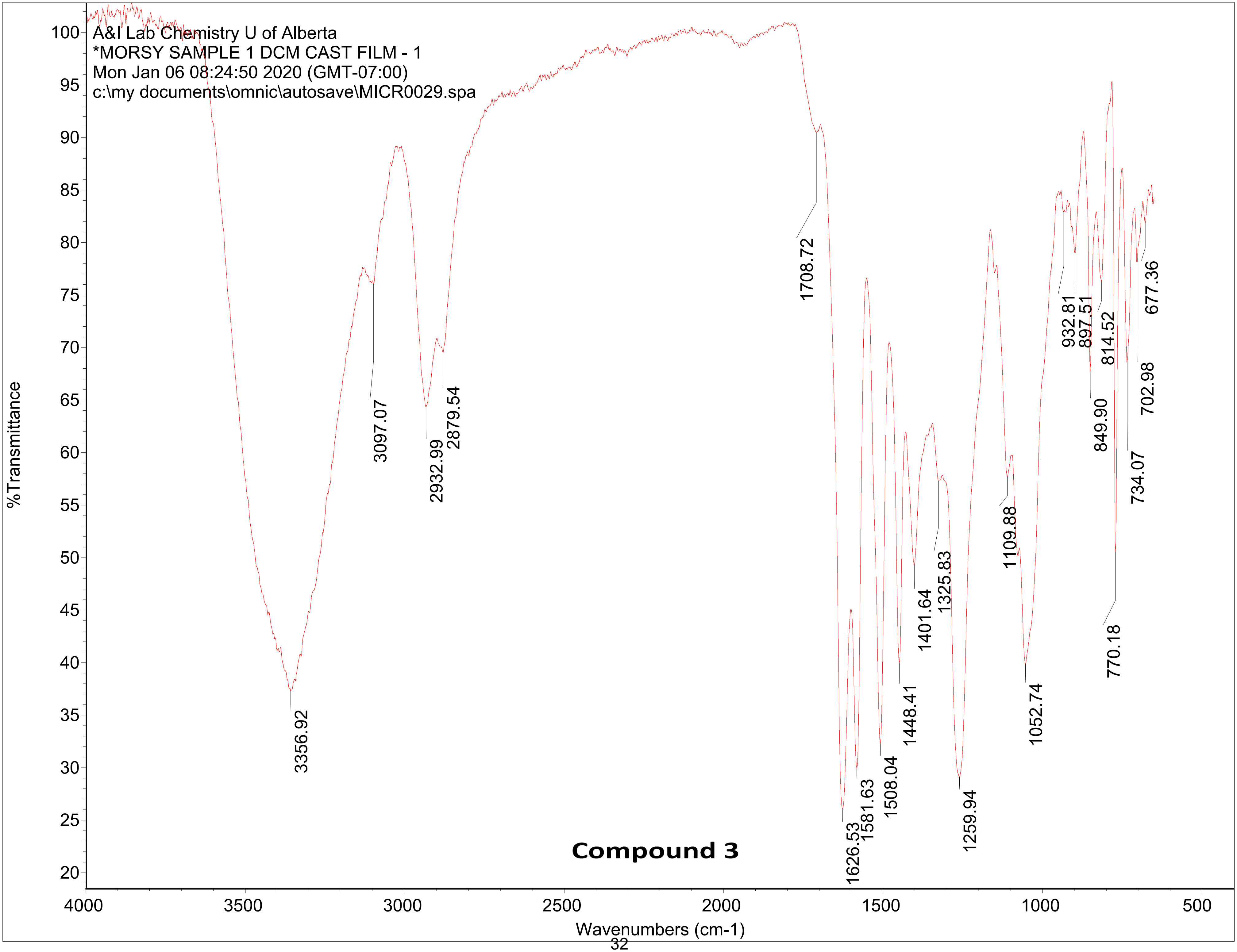
A&I Lab Chemistry U of Alberta
*MORSY SAMPLE 1 DCM CAST FILM - 1
Mon Jan 06 08:24:50 2020 (GMT-07:00)
c:\my documents\omnic\autosave\MICR0029.spa
%Transmittance
Wavenumbers (cm-1)
3356.92
3097.07
2932.99
2879.54
1708.72
1626.53
1581.63
1508.04
1448.41
1401.64
1325.83
1259.94
1109.88
1052.74
932.81
897.51
849.90
814.52
770.18
734.07
702.98
677.36

Compound 3

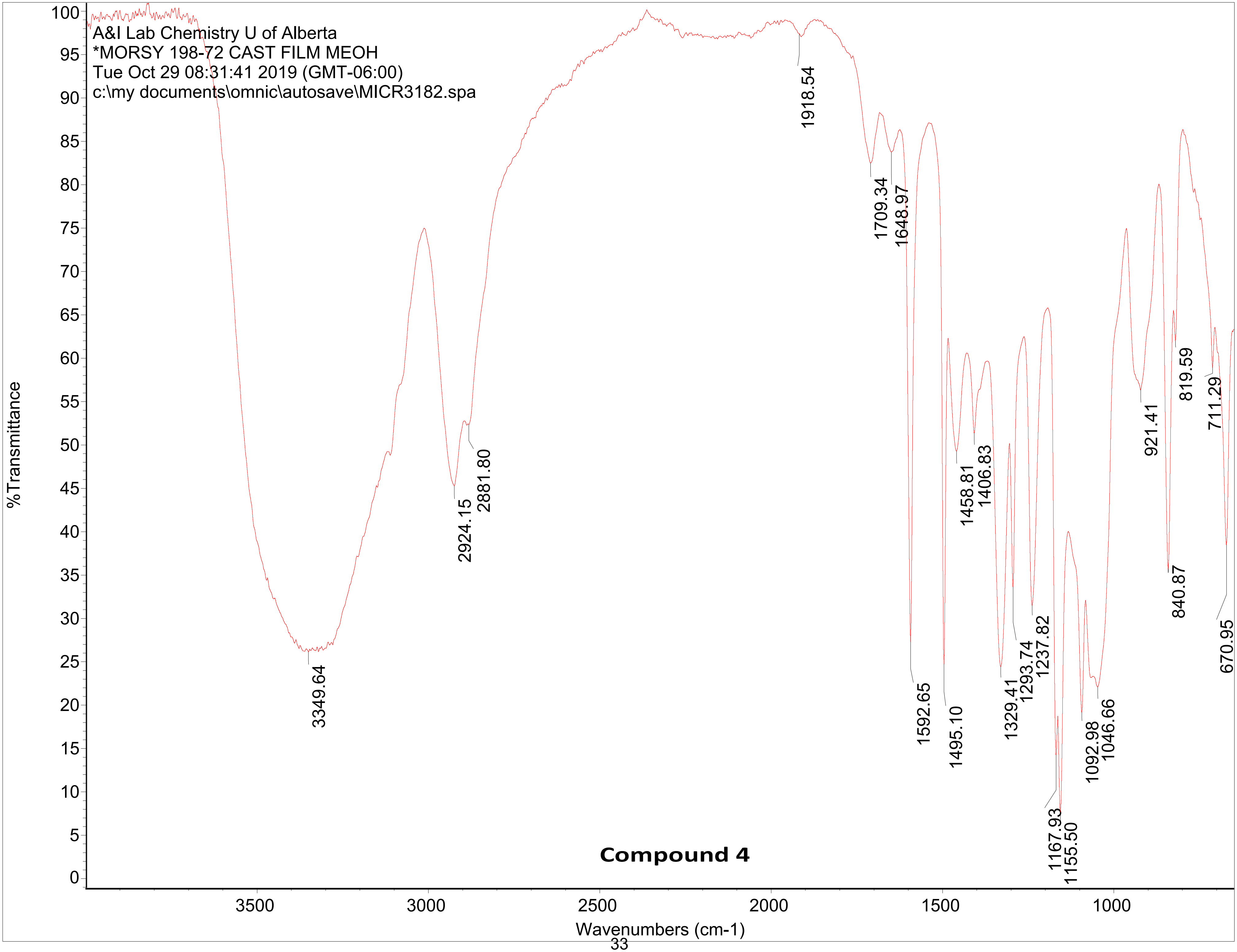
A&I Lab Chemistry U of Alberta
*MORSY 198-72 CAST FILM MEOH
Tue Oct 29 08:31:41 2019 (GMT-06:00)
c:\my documents\omnic\autosave\MICR3182.spa
%Transmittance
Wavenumbers (cm-1)
3349.64
2924.15
2881.80
1918.54
1709.34
1648.97
1592.65
1495.10
1458.81
1406.83
1329.41
1293.74
1237.82
1167.93
1155.50
1092.98
1046.66
921.41
840.87
819.59
711.29
670.95
Compound 4

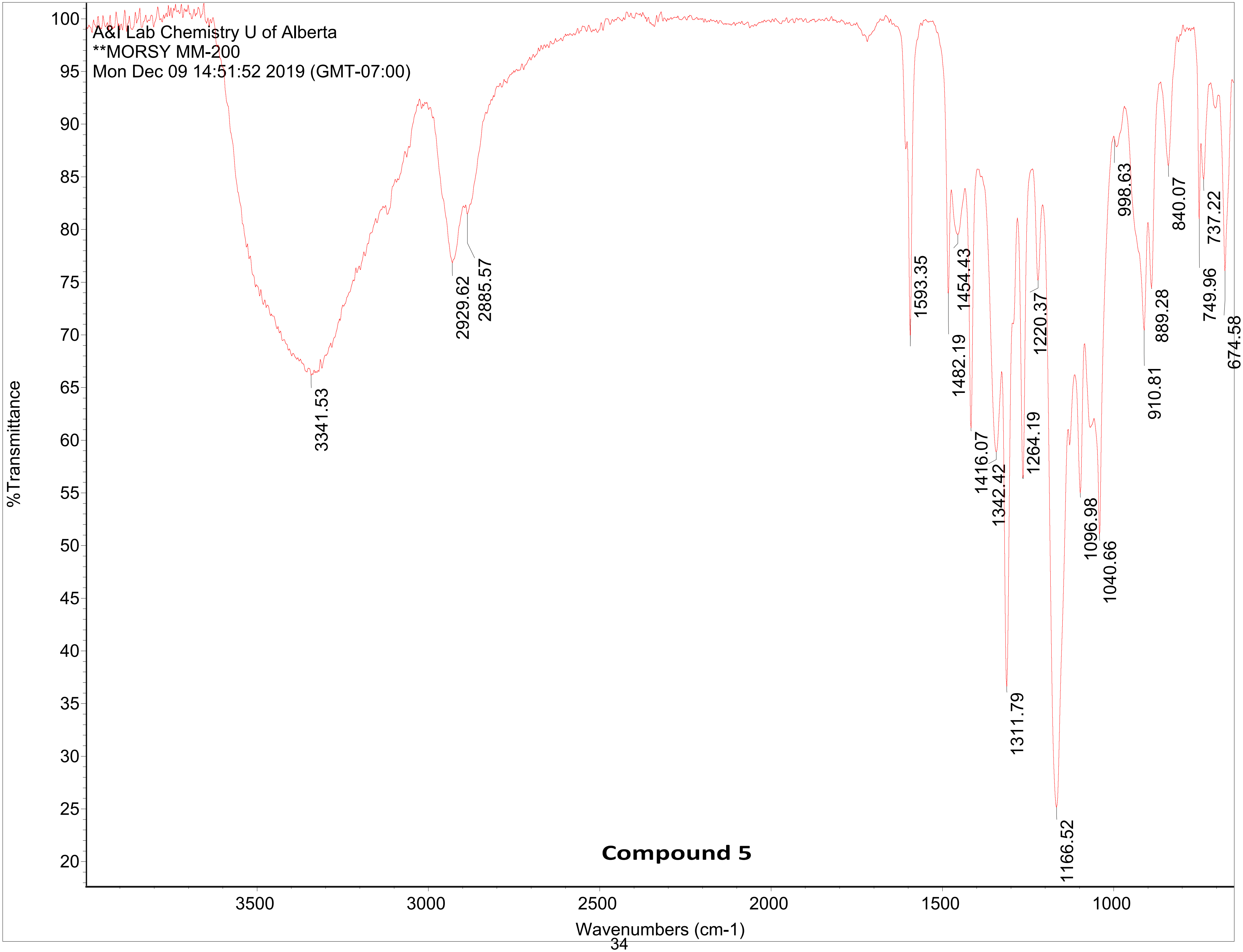
A&I Lab Chemistry U of Alberta
**MORSY MM-200
Mon Dec 09 14:51:52 2019 (GMT-07:00)
%Transmittance
Wavenumbers (cm-1)
3341.53
2929.62
2885.57
1593.35
1482.19
1454.43
1416.07
1342.42
1311.79
1264.19
1220.37
1166.52
1096.98
1040.66
998.63
910.81
889.28
840.07
749.96
737.22
674.58
Compound 5

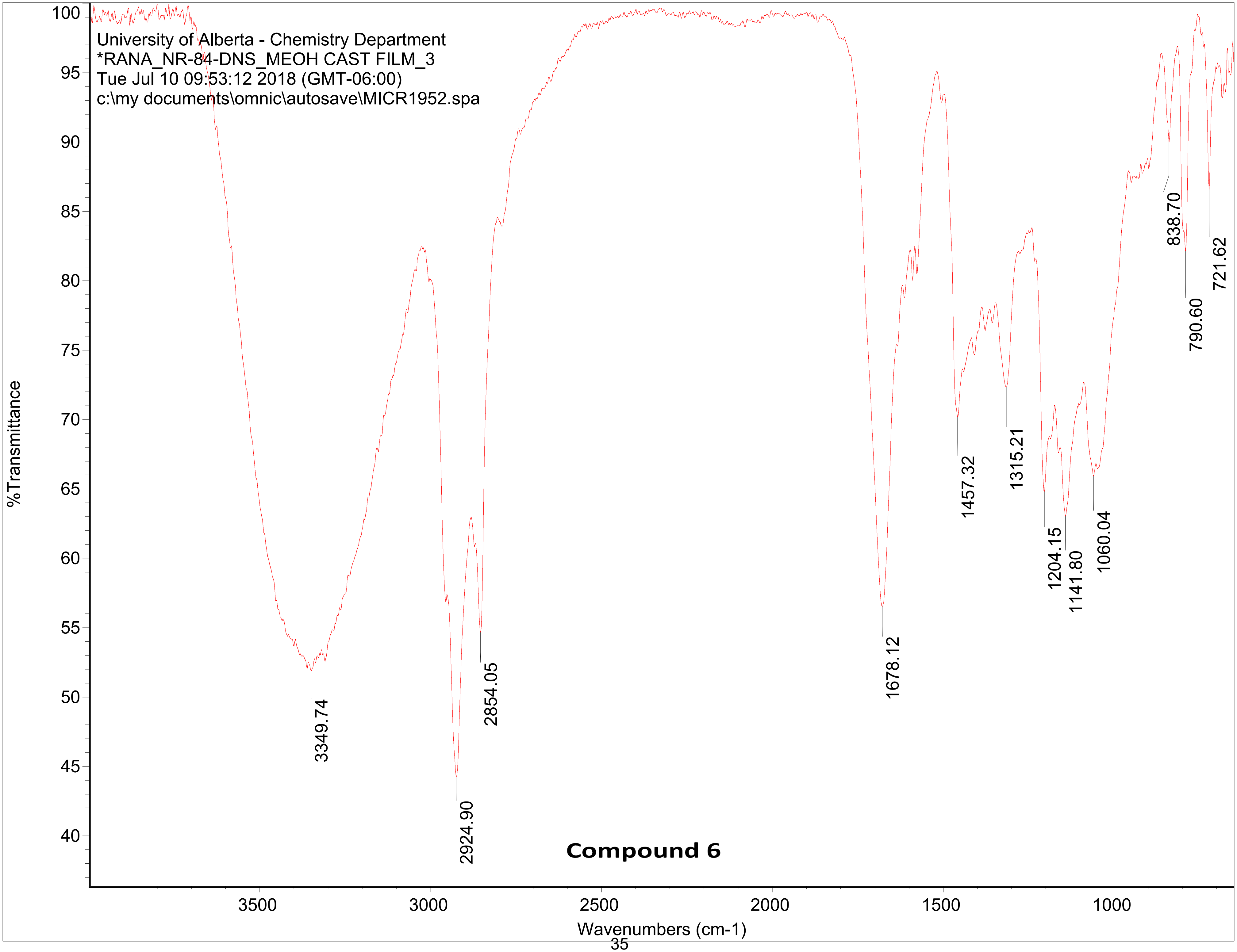
University of Alberta - Chemistry Department
*RANA_NR-84-DNS_MEOH CAST FILM_3
Tue Jul 10 09:53:12 2018 (GMT-06:00)
c:\my documents\omnic\autosave\MICR1952.spa
%Transmittance
Wavenumbers (cm-1)
3349.74
2924.90
2854.05
1678.12
1457.32
1315.21
1204.15
1141.80
1060.04
838.70
790.60
721.62
Compound 6

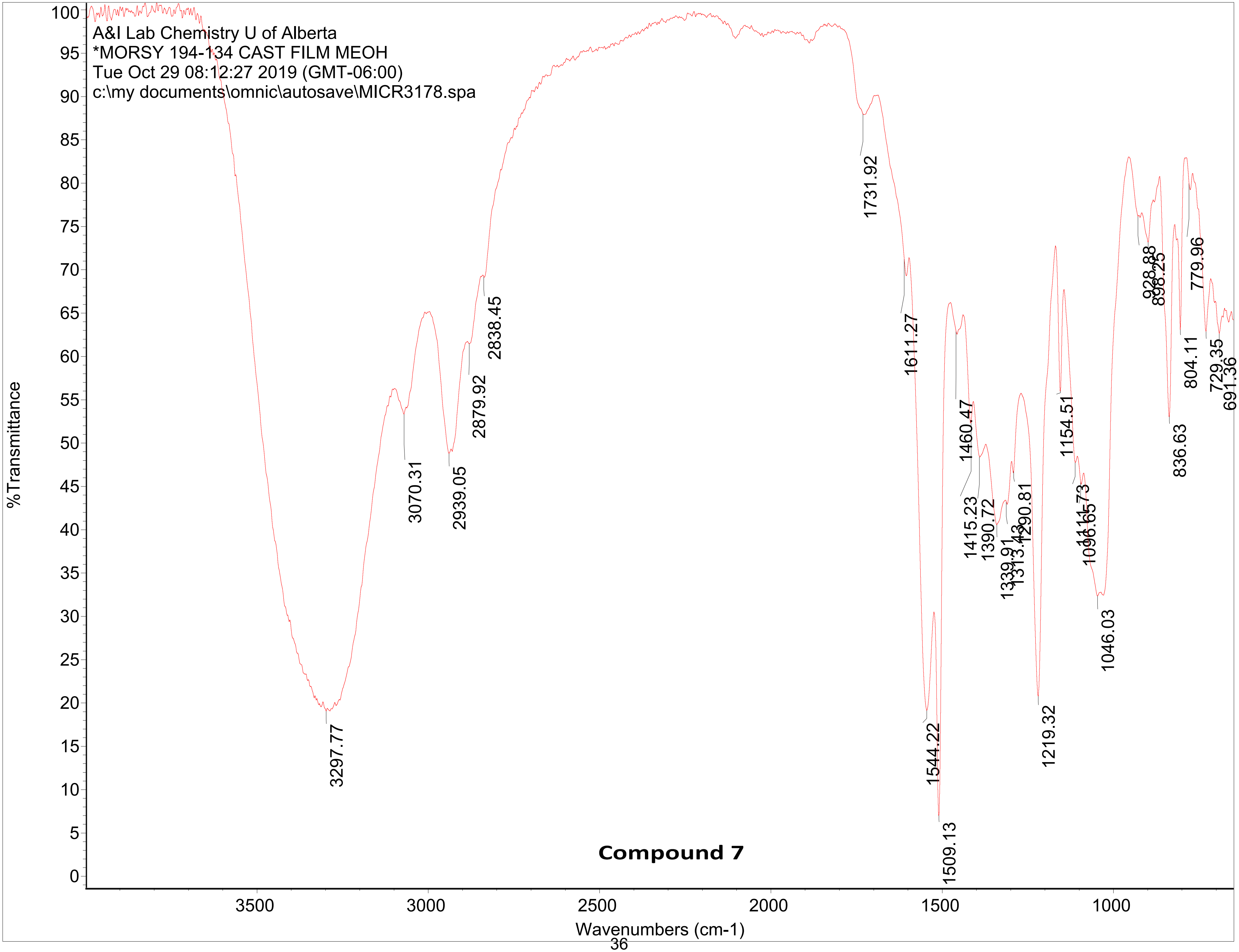
A&I Lab Chemistry U of Alberta
*MORSY 194-134 CAST FILM MEOH
Tue Oct 29 08:12:27 2019 (GMT-06:00)
c:\my documents\omnic\autosave\MICR3178.spa
%Transmittance
Wavenumbers (cm-1)
3297.77
3070.31
2939.05
2879.92
2838.45
1731.92
1611.27
1544.22
1509.13
1460.47
1415.23
1390.72
1339.91
1313.43
1290.81
1219.32
1154.51
1111.73
1096.65
1046.03
928.88
898.25
836.63
804.11
779.96
729.35
691.36

Compound 7

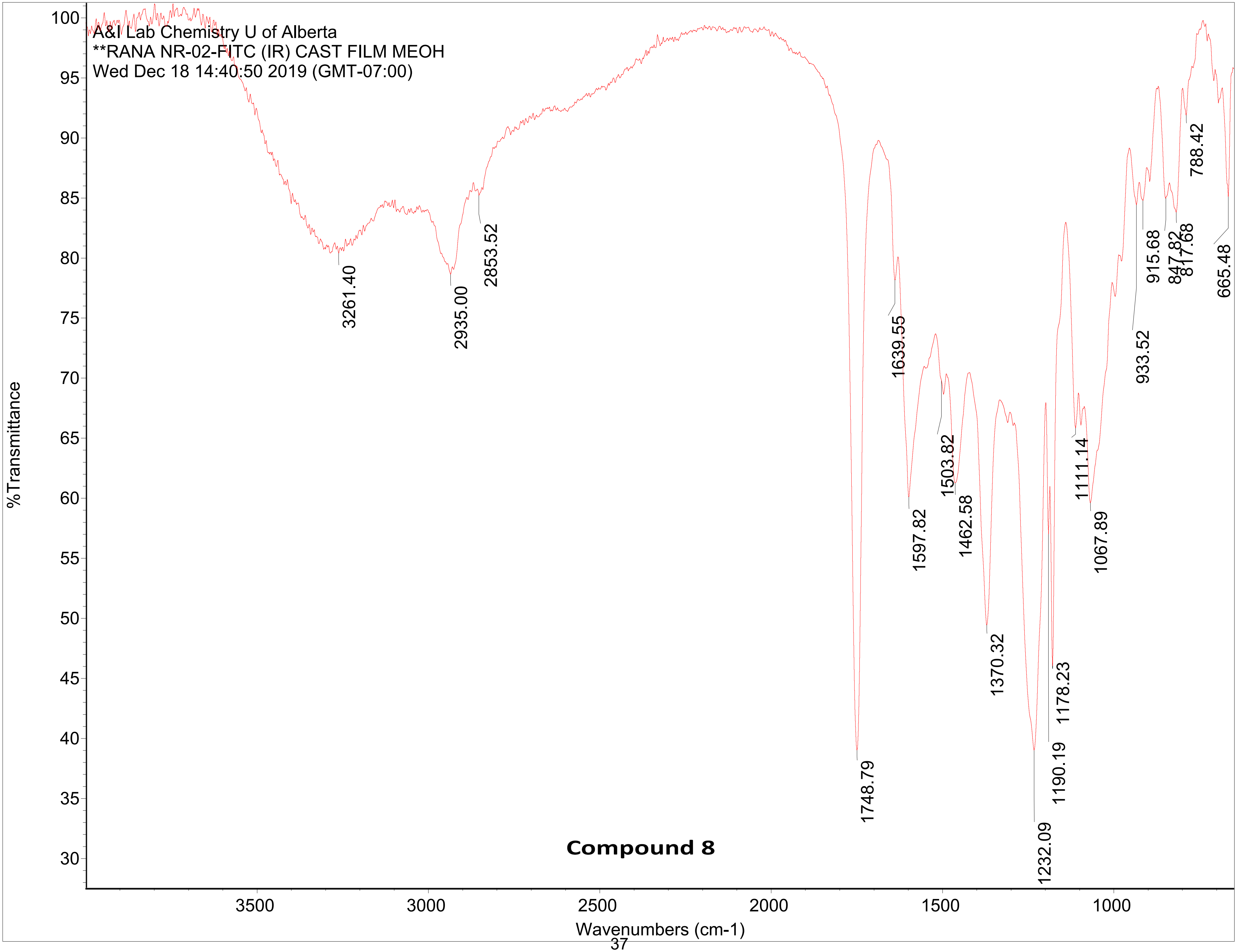
A&I Lab Chemistry U of Alberta
**RANA NR-02-FITC (IR) CAST FILM MEOH
Wed Dec 18 14:40:50 2019 (GMT-07:00)
%Transmittance
Wavenumbers (cm-1)
3261.40
2935.00
2853.52
1748.79
1639.55
1597.82
1503.82
1462.58
1370.32
1232.09
1190.19
1178.23
1111.14
1067.89
933.52
915.68
847.82
817.68
788.42
665.48
Compound 8

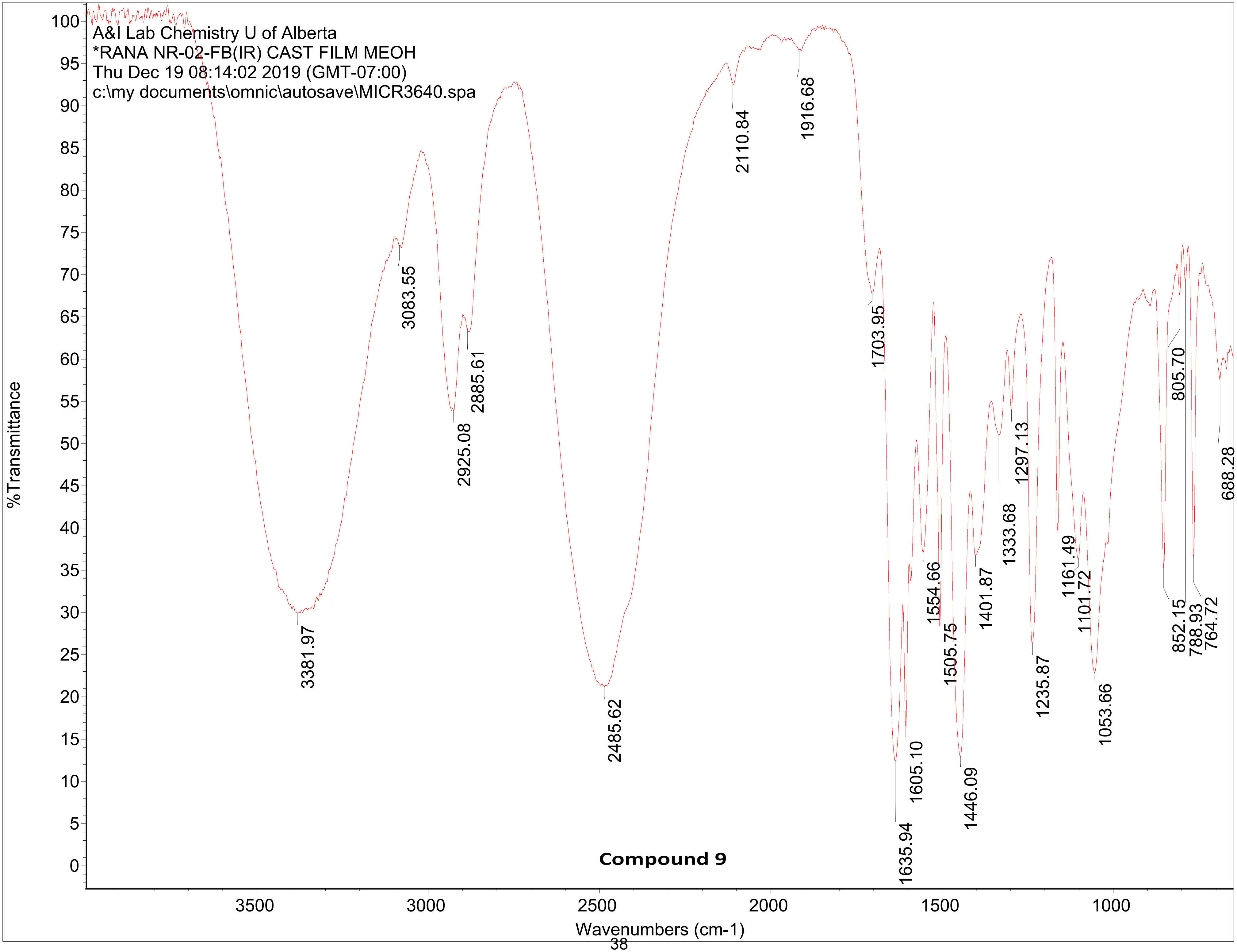
A&I Lab Chemistry U of Alberta
*RANA NR-02-FB(IR) CAST FILM MEOH
Thu Dec 19 08:14:02 2019 (GMT-07:00)
c:\my documents\omnic\autosave\MICR3640.spa
%Transmittance
Wavenumbers (cm-1)
3381.97
3083.55
2925.08
2885.61
2485.62
2110.84
1916.68
1703.95
1635.94
1605.10
1554.66
1505.75
1446.09
1401.87
1333.68
1297.13
1235.87
1161.49
1101.72
1053.66
852.15
805.70
788.93
764.72
688.28

Compound 9

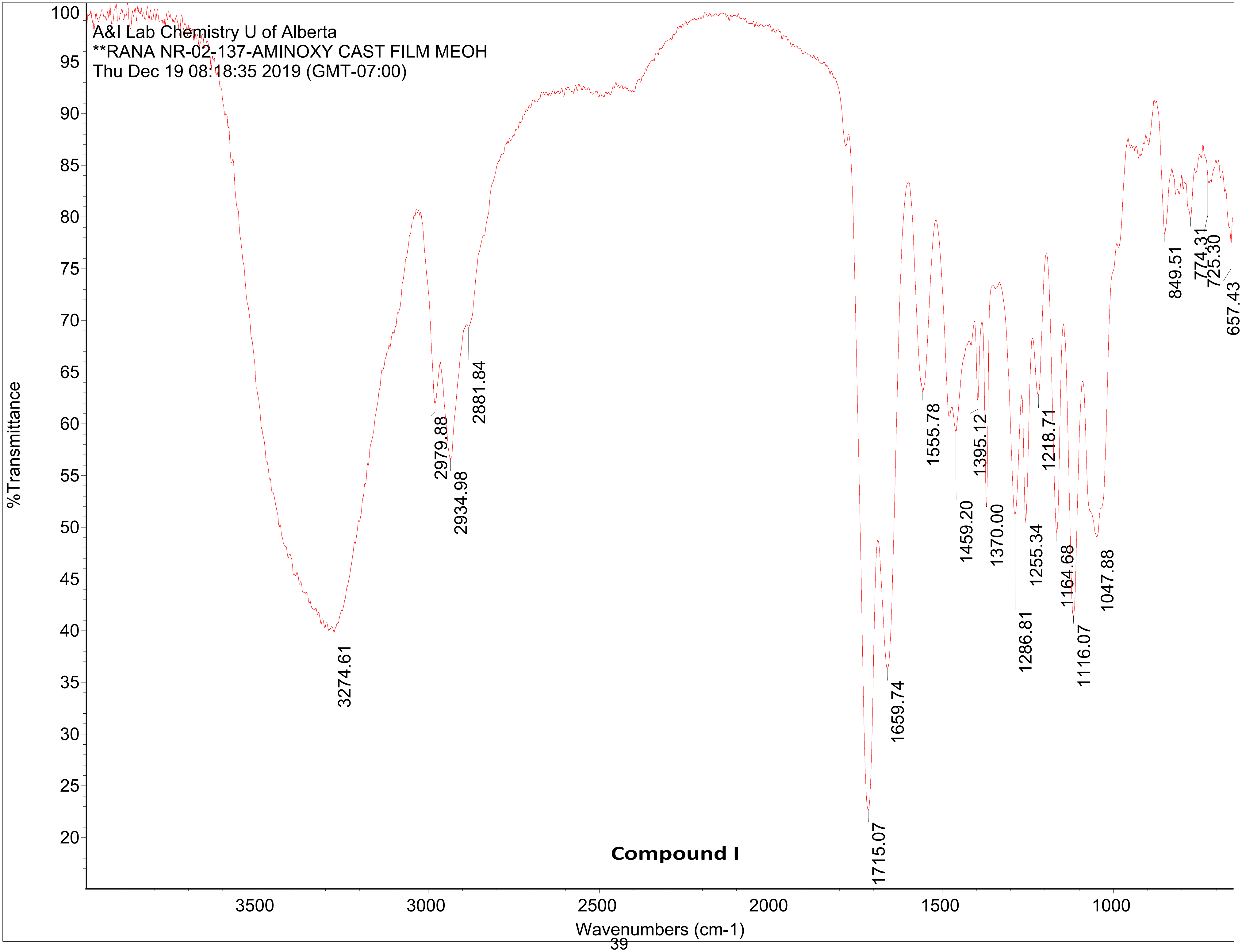
A&I Lab Chemistry U of Alberta
**RANA NR-02-137-AMINOXY CAST FILM MEOH
Thu Dec 19 08:18:35 2019 (GMT-07:00)
%Transmittance
Wavenumbers (cm-1)
3274.61
2979.88
2934.98
2881.84
1715.07
1659.74
1555.78
1459.20
1395.12
1370.00
1286.81
1255.34
1218.71
1164.68
1116.07
1047.88
849.51
774.31
725.30
657.43
Compound I

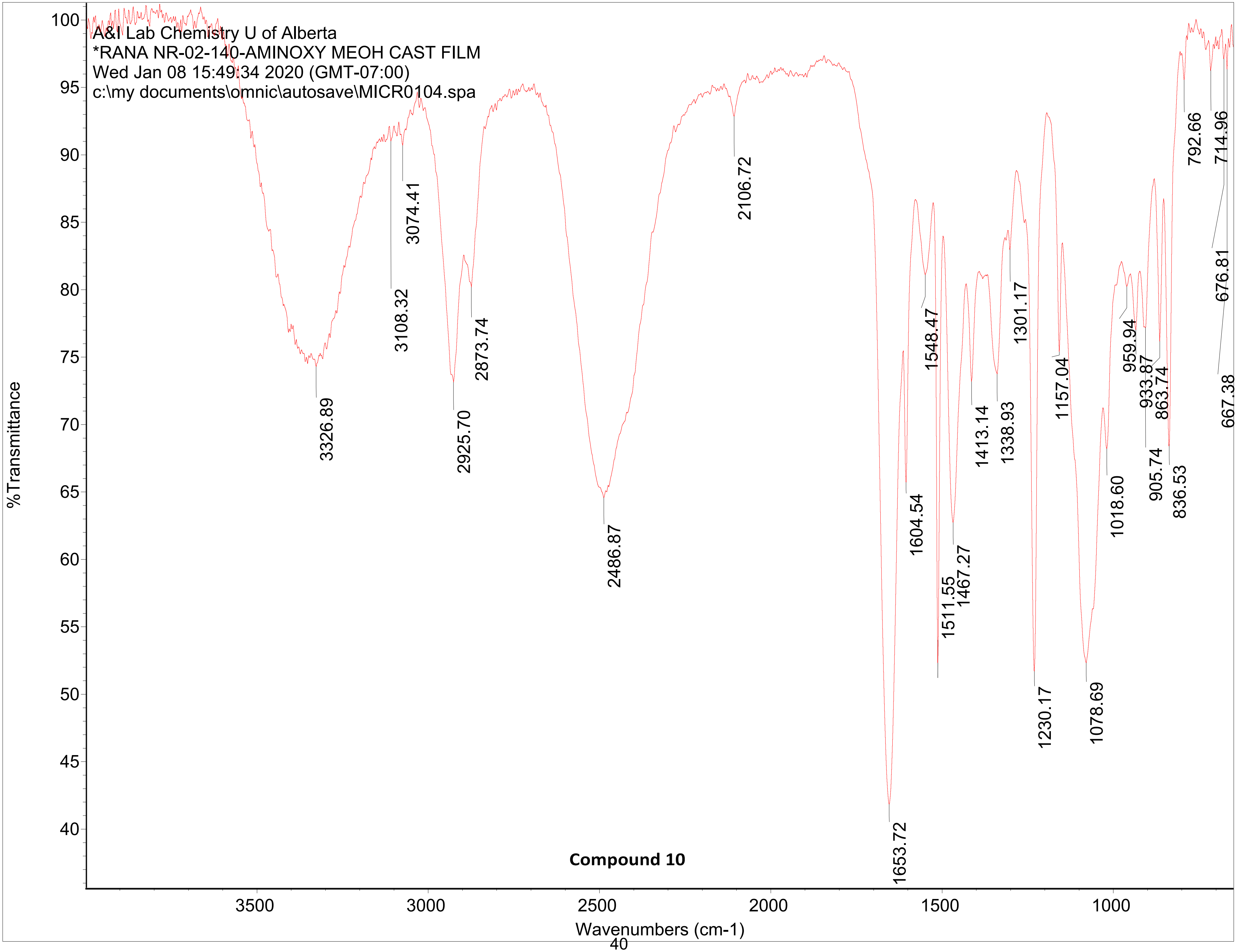
A&I Lab Chemistry U of Alberta
*RANA NR-02-140-AMINOXY MEOH CAST FILM
Wed Jan 08 15:49:34 2020 (GMT-07:00)
c:\my documents\omnic\autosave\MICR0104.spa
%Transmittance
Wavenumbers (cm-1)
3326.89
3108.32
3074.41
2925.70
2873.74
2486.87
2106.72
1653.72
1604.54
1548.47
1511.55
1467.27
1413.14
1338.93
1301.17
1230.17
1157.04
1078.69
1018.60
959.94
933.87
905.74
863.74
836.53
792.66
714.96
676.81
667.38

Compound 10

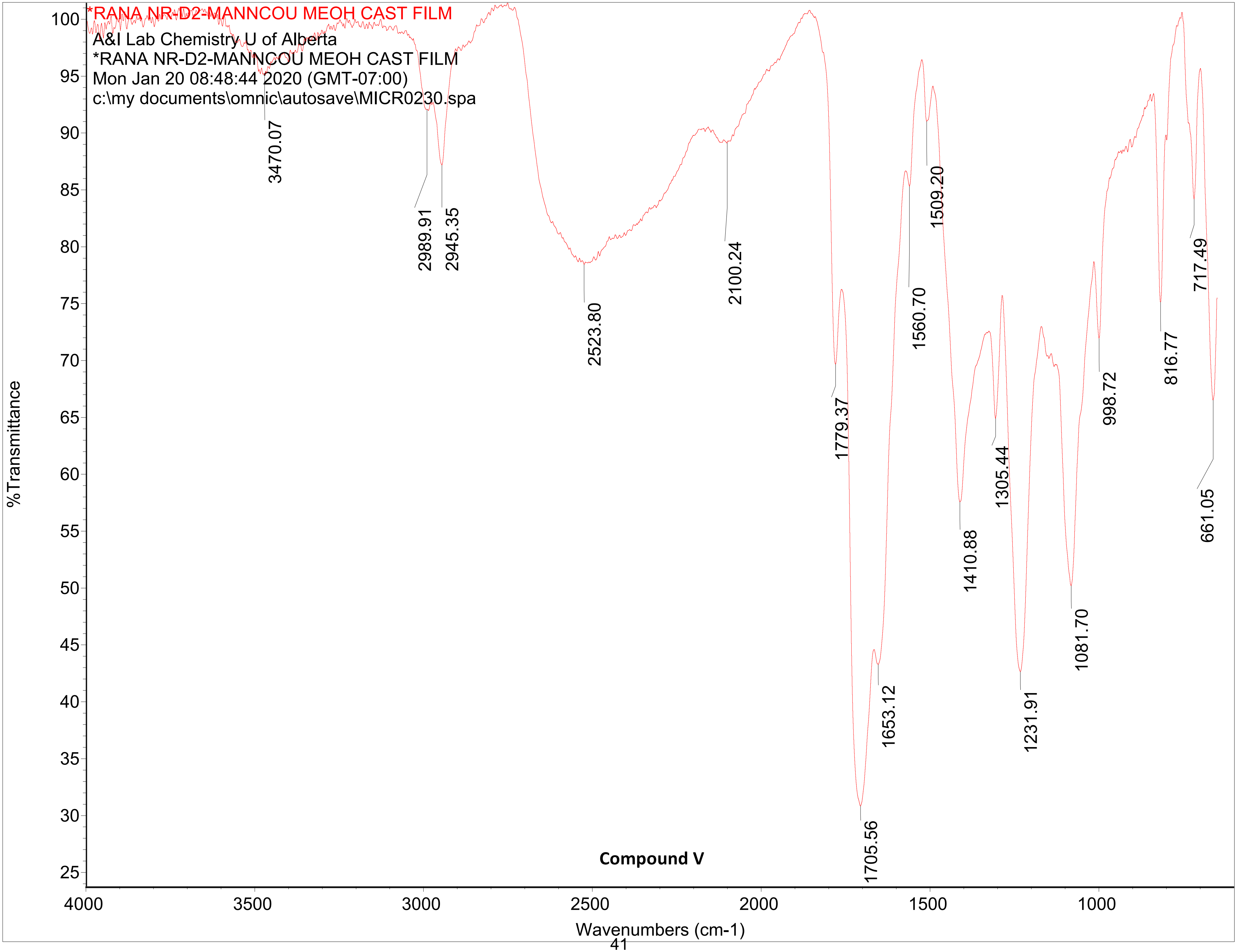
*RANA NR-D2-MANNCOU MEOH CAST FILM
A&I Lab Chemistry U of Alberta
*RANA NR-D2-MANNCOU MEOH CAST FILM
Mon Jan 20 08:48:44 2020 (GMT-07:00)
c:\my documents\omnic\autosave\MICR0230.spa
%Transmittance
Wavenumbers (cm-1)
3470.07
2989.91
2945.35
2523.80
2100.24
1779.37
1705.56
1653.12
1560.70
1509.20
1410.88
1305.44
1231.91
1081.70
998.72
816.77
717.49
661.05
Compound V

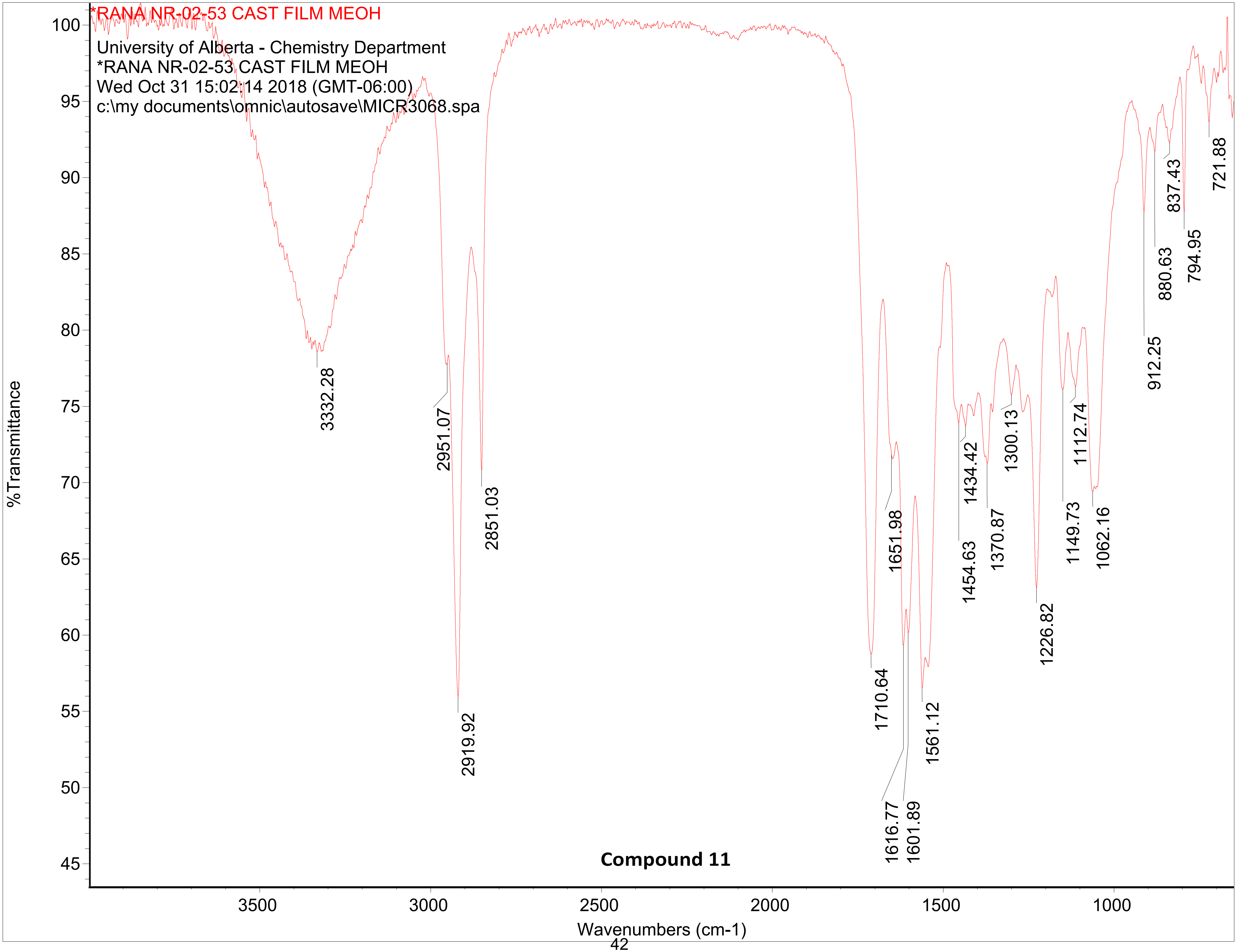
*RANA NR-02-53 CAST FILM MEOH
University of Alberta - Chemistry Department
*RANA NR-02-53 CAST FILM MEOH
Wed Oct 31 15:02:14 2018 (GMT-06:00)
c:\my documents\omnic\autosave\MICR3068.spa
%Transmittance
Wavenumbers (cm-1)
3332.28
2951.07
2919.92
2851.03
1710.64
1651.98
1616.77
1601.89
1561.12
1454.63
1434.42
1370.87
1300.13
1226.82
1149.73
1112.74
1062.16
912.25
880.63
837.43
794.95
721.88

Compound 11

Department of Chemistry Mass Spectrometry Laboratory

**Name**	M. Morsy, West	**Sample Name**	mm-02-34
**Data Filename**	20021012.d	**Instrument Name**	oaTOF6220
**Position**	-1	**Operator**	ami
**Acq Method**		**DA Method**	da ami low mass.m

User Spectra

Fragmentor Voltage	Source Type
100	Esi

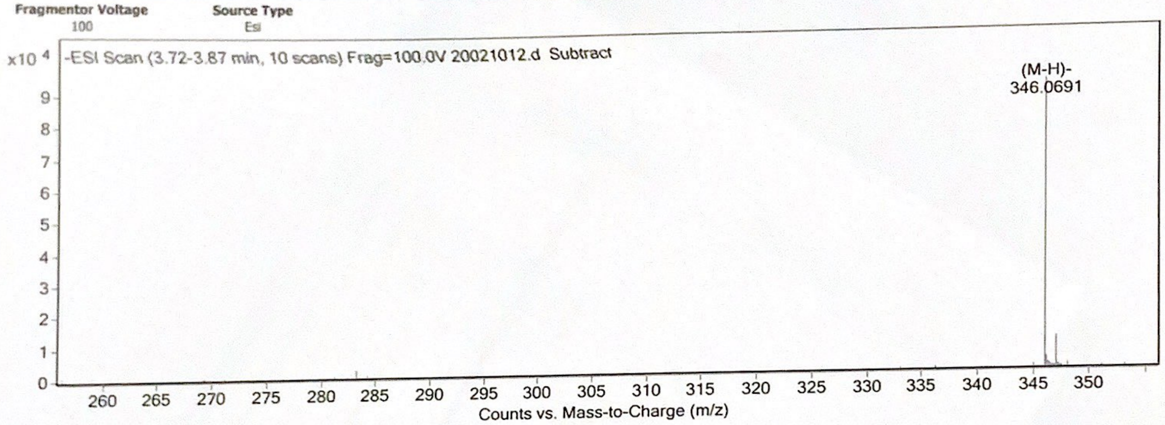

Compound 3

Formula Calculator Results

Formula	Ion Species	Mass	Calc. Mass	m/z	Calc. m/z	Diff (mDa)	Diff (ppm)	DBE	Ion	Score
C12 H14 F N3 O8	C12 H13 F N3 O8	347.0764	347.0765	346.0691	346.0692	0.1	0.28	7	(M-H)-	94.12

--- End Of Report ---

Mass Spectrometry Facility,Dept. of Chemistry
Univ. of Alberta,1-780-492-5577

Page 1 of 1

Printed at: 4:34 PM on: 2/10/2020

Department of Chemistry Mass Spectrometry Laboratory

**Name**	M. Morsy, West	**Sample Name**	mm-198-72
**Data Filename**	19102514.d	**Instrument Name**	oaTOF6220
**Position**	-1	**Operator**	ami
**Acq Method**		**DA Method**	da ami low mass.m

User Spectra

Fragmentor Voltage	Source Type
200	Esi

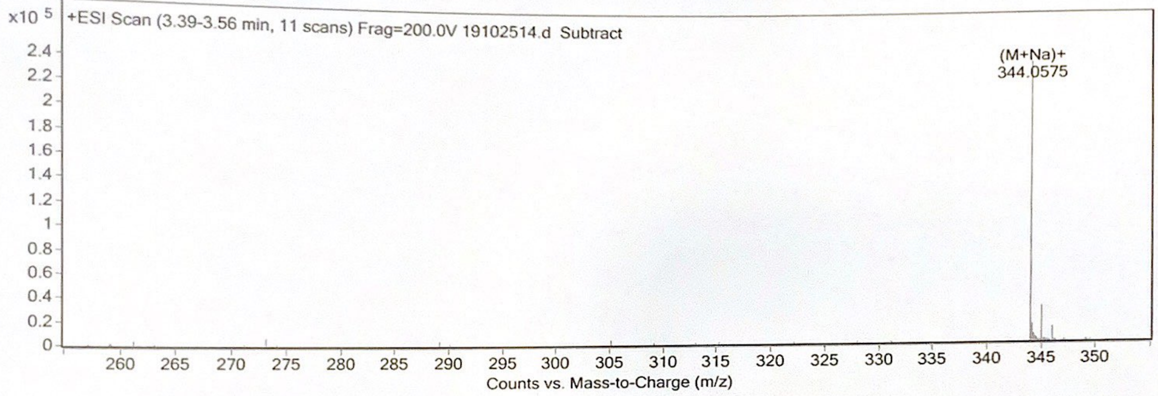

Compound 4

Formula Calculator Results

Formula	Ion Species	Mass	Calc. Mass	m/z	Calc. m/z	Diff (mDa)	Diff (ppm)	DBE	Ion	Score
C12 H16 F N O6 S	C12 H16 F N Na O6 S	321.0683	321.0682	344.0575	344.0575	-0.05	-0.13	5	(M+Na)+	96.51

--- End Of Report ---

Department of Chemistry Mass Spectrometry Laboratory

**Name**	M. Morsy, West	**Sample Name**	mm-200
**Data Filename**	19120905.d	**Instrument Name**	oaTOF6220
**Position**	-1	**Operator**	ami
**Acq Method**		**DA Method**	da ami low mass.m

User Spectra

Fragmentor Voltage	Source Type
100	Esi

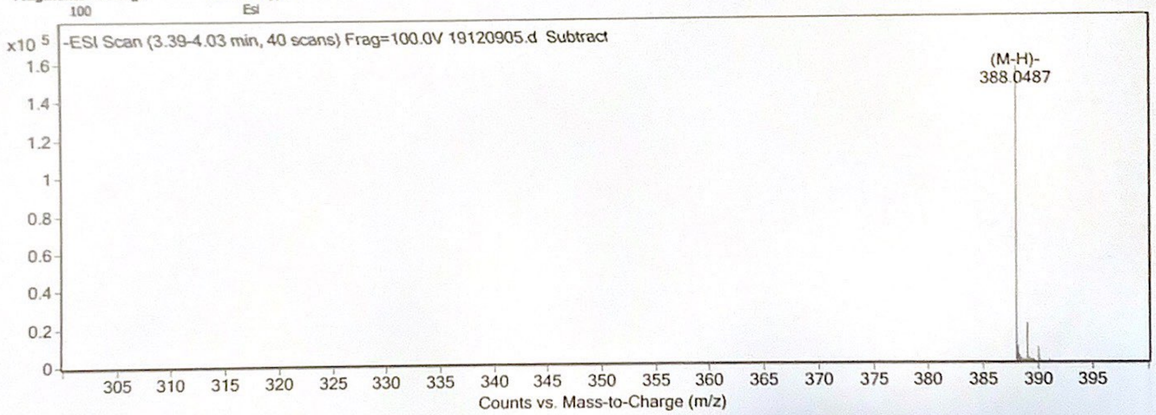

Compound 5

Formula Calculator Results

Formula	Ion Species	Mass	Calc. Mass	m/z	Calc. m/z	Diff (mDa)	Diff (ppm)	DBE	Ion	Score
C13 H15 F4 N O6 S	C13 H14 F4 N O6 S	389.0559	389.0556	388.0487	388.0483	-0.32	-0.82	5	(M-H)-	89.36

--- End Of Report ---

Department of Chemistry Mass Spectròmetry Laboratory

**Name**	N. Rana, West	**Sample Name**	nr-84-dns
**Data Filename**	18013011.d	**Instrument Name**	oaTOF6220
**Position**	-1	**Operator**	ami
**Acq Method**		**DA Method**	da ami low mass.m

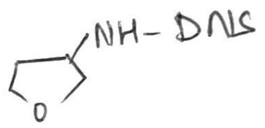

User Spectra

Fragmentor Voltage 200 **Source Type** Esi

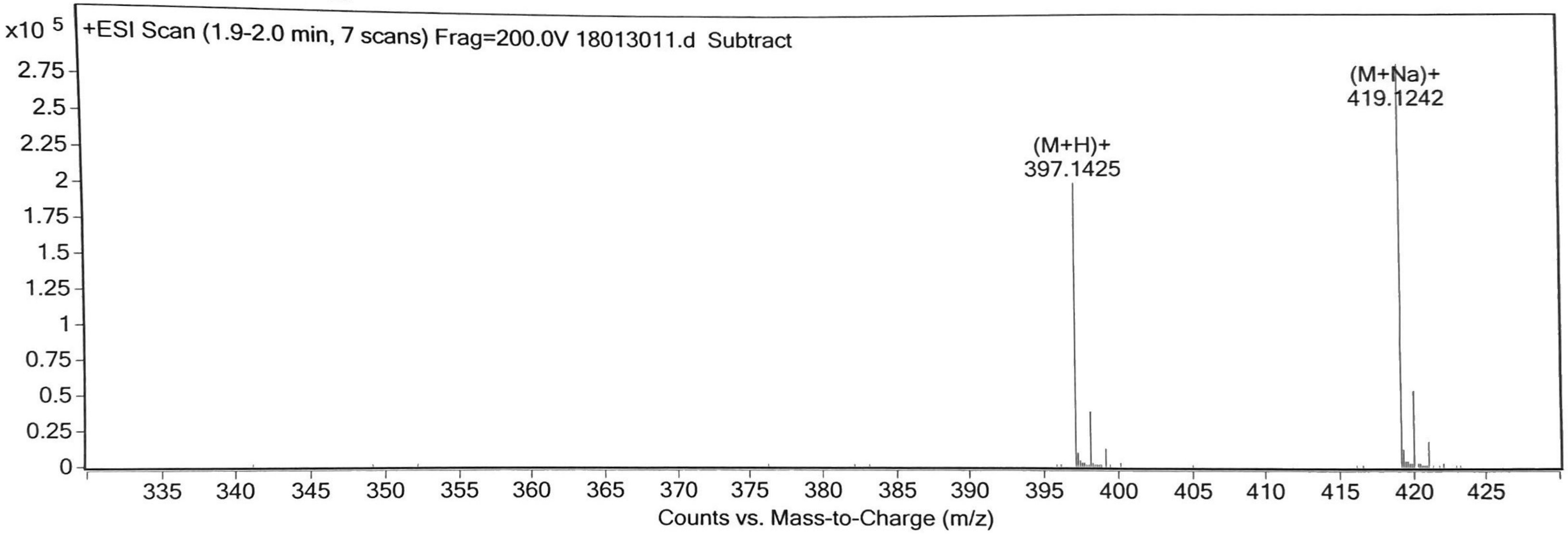

Compound 6

Formula Calculator Results

Formula	Ion Species	Mass	Calc. Mass	m/z	Calc. m/z	Diff (mDa)	Diff (ppm)	DBE	Ion	Score
C18 H24 N2 O6 S	C18 H25 N2 O6 S	396.1353	396.1355	397.1425	397.1428	0.26	0.65	8	(M+H)+	90.92
C18 H24 N2 O6 S	C18 H24 N2 Na O6 S	396.1349	396.1355	419.1242	419.1247	0.57	1.36	8	(M+Na)+	88.71

--- End Of Report ---

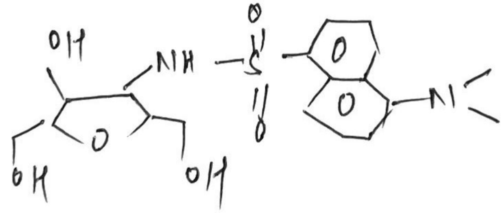

Please bring solvent free sample
thanks

Department of Chemistry Mass Spectrometry Laboratory

**Name**	M. Morsy, West	**Sample Name**	mm-194
**Data Filename**	19101820.d	**Instrument Name**	oaTOF6220
**Position**	-1	**Operator**	ami
**Acq Method**		**DA Method**	da ami low mass.m

User Spectra

**Fragmentor Voltage**	**Source Type**
200	Esi

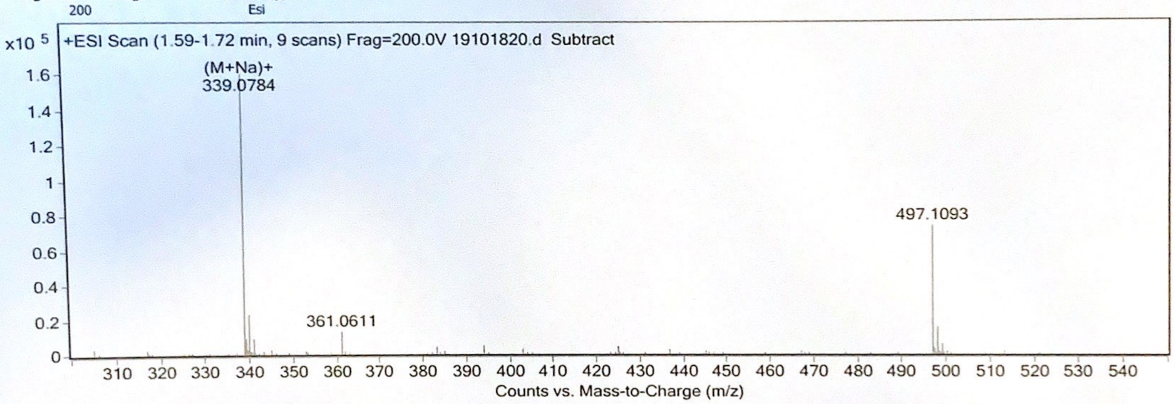

Compound 7

Formula Calculator Results

Formula	Ion Species	Mass	Calc. Mass	m/z	Calc. m/z	Diff (mDa)	Diff (ppm)	DBE	Ion	Score
C13 H17 F N2 O4 S	C13 H17 F N2 Na O4 S	316.0892	316.0893	339.0784	339.0785	0.16	0.46	6	(M+Na)+	90.83

--- End Of Report ---

Department of Chemistry Mass Spectrometry Laboratory

**Name**	N. Rana, West	**Sample Name**	nr-02-fitc [ms]
**Data Filename**	19121907.d	**Instrument Name**	oaTOF6220
**Position**	-1	**Operator**	ami
**Acq Method**		**DA Method**	da ami low mass.m

User Spectra

Fragmentor Voltage 100 **Source Type** Esi

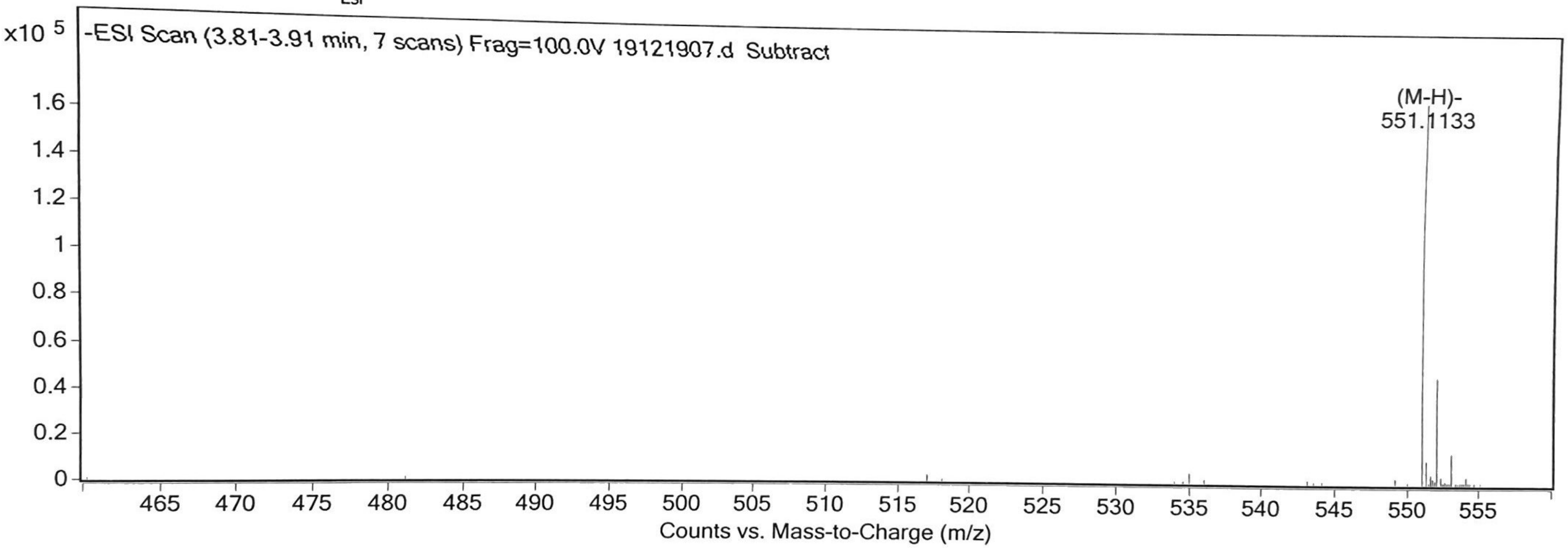

Compound 8

Dec 18

Formula Calculator Results

Formula	Ion Species	Mass	Calc. Mass	m/z	Calc. m/z	Diff (mDa)	Diff (ppm)	DBE	Ion	Score
C27 H24 N2 O9 S	C27 H23 N2 O9 S	552.1206	552.1203	551.1133	551.113	-0.33	-0.6	17	(M-H)-	91.61

--- End Of Report ---

Department of Chemistry Mass Spectrometry Laboratory

Name	N. Rana, West	**Sample Name**	nr-147-fb
Data Filename	18062921.d	**Instrument Name**	oaTOF6220
Position	-1	**Operator**	ami
Acq Method		**DA Method**	da ami low mass.m

nr-147-1

User Spectra

Fragmentor Voltage 200 **Source Type** Esi

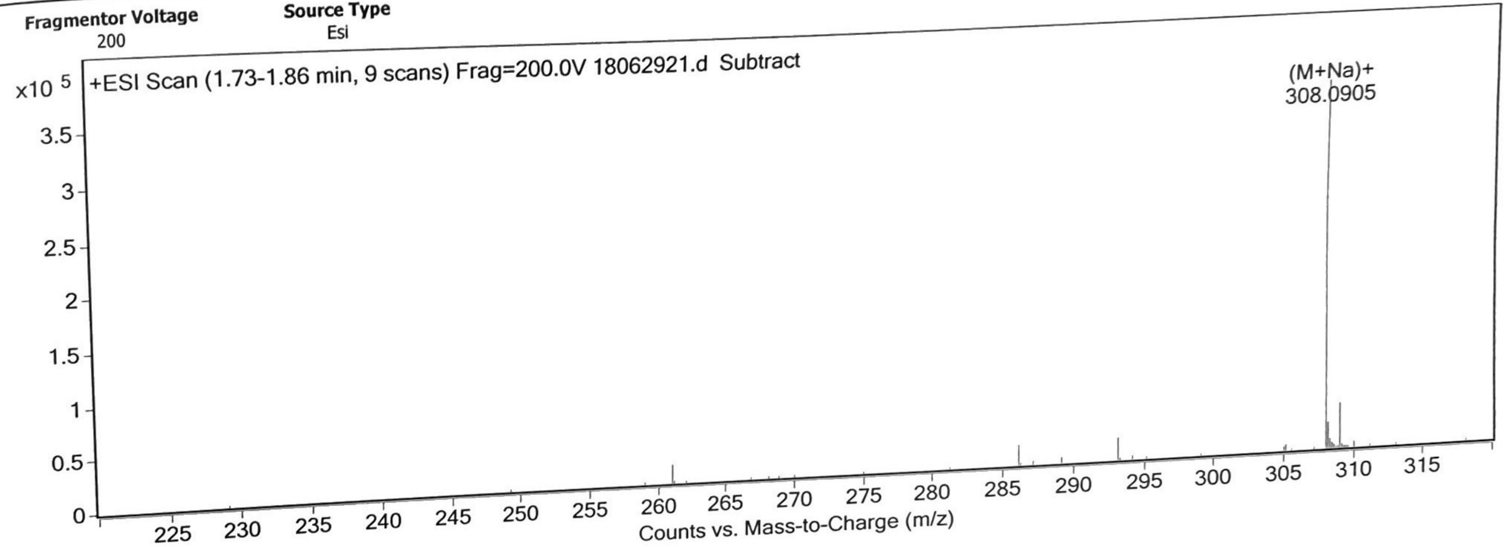

Compound 9

Dec 19

Formula Calculator Results

Formula	Ion Species	Mass	Calc. Mass	m/z	Calc. m/z	Diff (mDa)	Diff (ppm)	DBE	Ion	Score
C13 H16 F N O5	C13 H16 F N Na O5	285.1013	285.1013	308.0905	308.0905	-0.01	-0.03	6	(M+Na)+	91.08

--- End Of Report ---

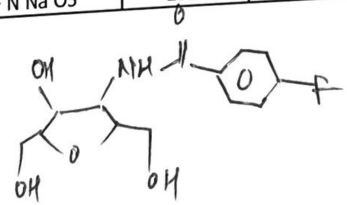

Department of Chemistry Mass Spectrometry Laboratory

**Name**	N. Rana, West	**Sample Name**	nr-02-137-aminoxy
**Data Filename**	19121924.d	**Instrument Name**	oaTOF6220
**Position**	-1	**Operator**	ami
**Acq Method**		**DA Method**	da ami low mass.m

User Spectra

Fragmentor Voltage 100 **Source Type** Esi

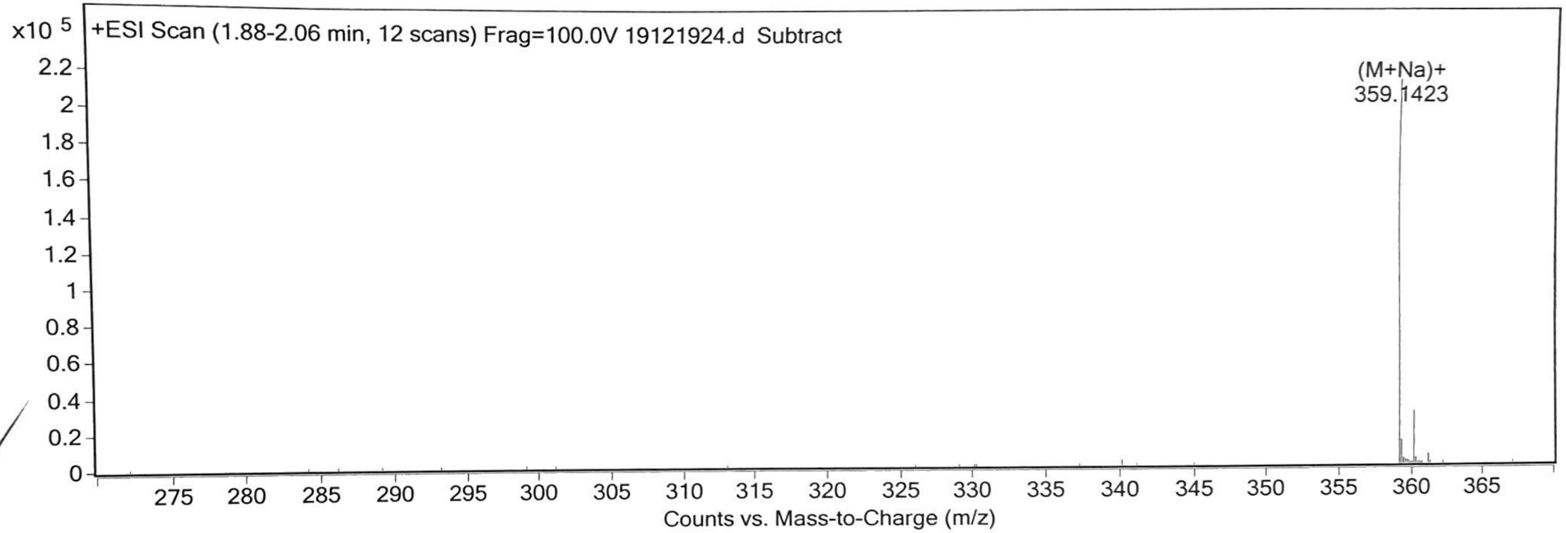

Compound I

Dec 19

Formula Calculator Results

Formula	Ion Species	Mass	Calc. Mass	m/z	Calc. m/z	Diff (mDa)	Diff (ppm)	DBE	Ion	Score
C13 H24 N2 O8	C13 H24 N2 Na O8	336.1531	336.1533	359.1423	359.1425	0.17	0.47	3	(M+Na)+	93.46

--- End Of Report ---

Department of Chemistry Mass Spectrometry Laboratory

**Name**	N. Rana, West	**Sample Name**	nr-02-140-aminoxy
**Data Filename**	20010703.d	**Instrument Name**	oaTOF6220
**Position**	-1	**Operator**	ami
**Acq Method**		**DA Method**	da ami low mass.m

User Spectra

Fragmentor Voltage	Source Type
100	Esi

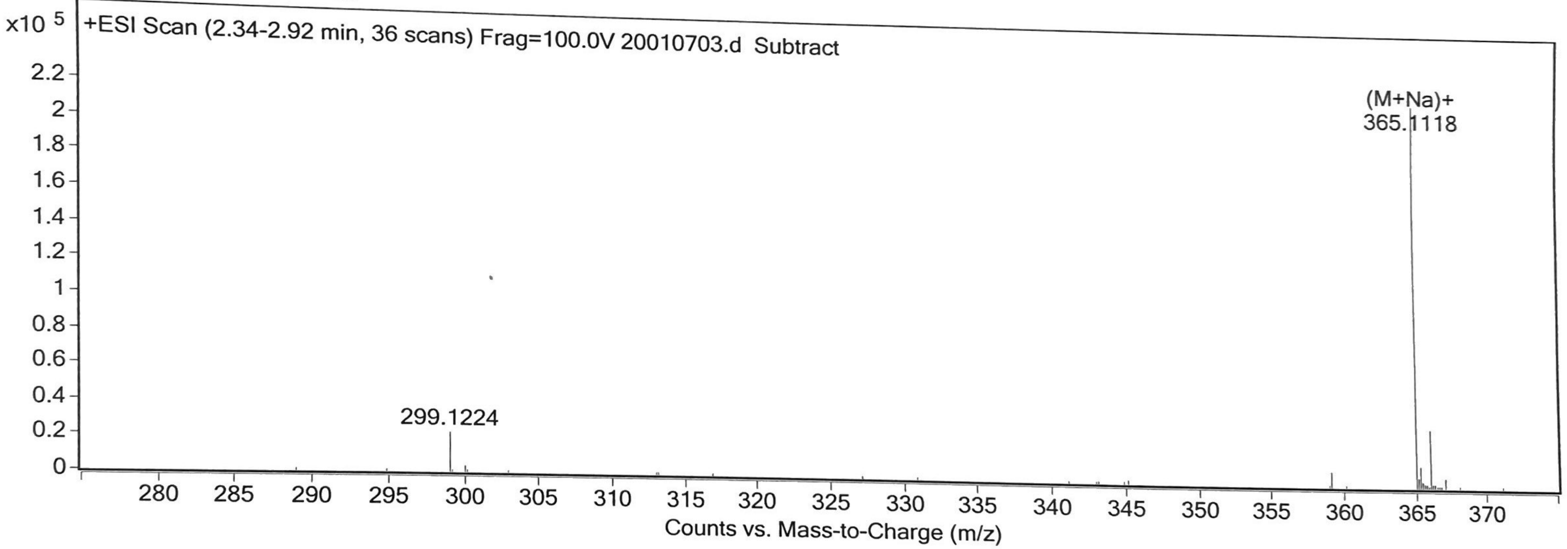

Compound 10

Jan 8

Formula Calculator Results

Formula	Ion Species	Mass	Calc. Mass	m/z	Calc. m/z	Diff (mDa)	Diff (ppm)	DBE	Ion	Score
C15 H19 F N2 O6	C15 H19 F N2 Na O6	342.1226	342.1227	365.1118	365.1119	0.14	0.4	7	(M+Na)+	92.51

--- End Of Report ---

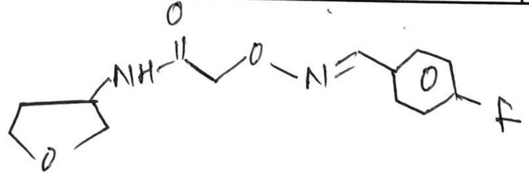

Department of Chemistry Mass Spectrometry Laboratory

**Name**	N. Rana, West	**Sample Name**	nr-02-mancou
**Data Filename**	20011709.d	**Instrument Name**	oaTOF6220
**Position**	-1	**Operator**	ami
**Acq Method**		**DA Method**	da ami low mass.m

User Spectra

Fragmentor Voltage 100 **Source Type** Esi

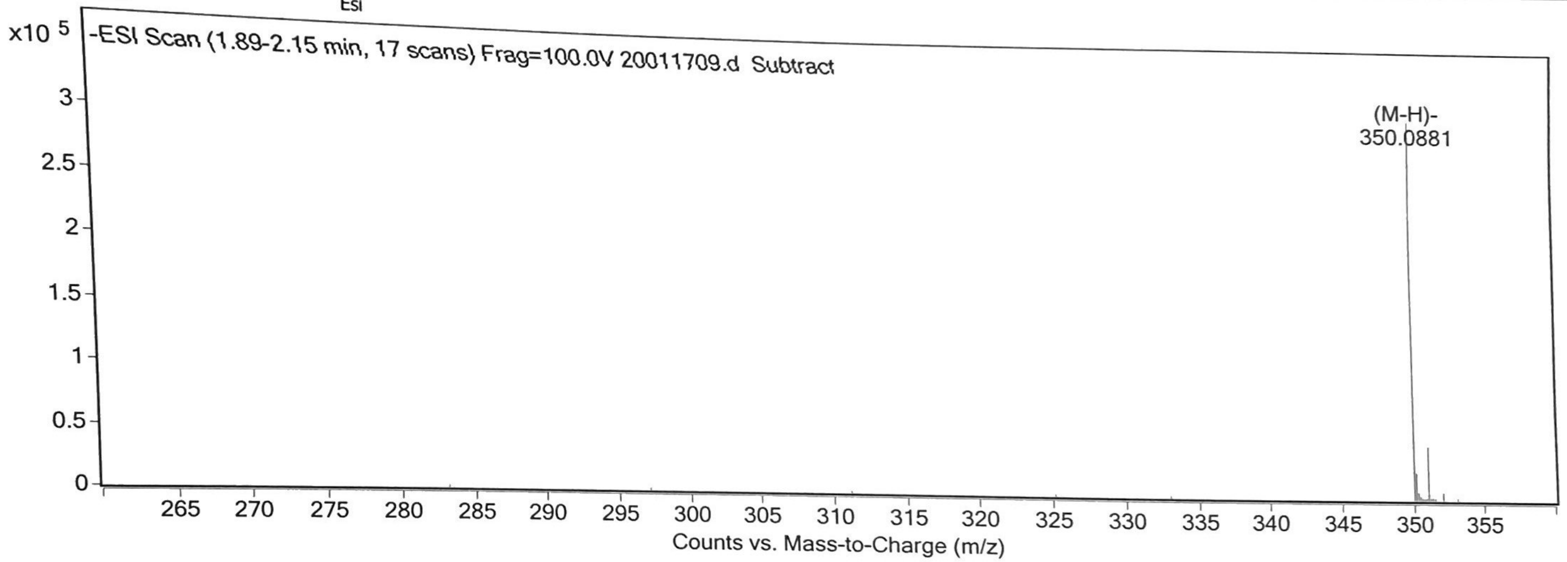

Compound V

Jan 20

Formula Calculator Results

Formula	Ion Species	Mass	Calc. Mass	m/z	Calc. m/z	Diff (mDa)	Diff (ppm)	DBE	Ion	Score
C16 H17 N O8	C16 H16 N O8	351.0954	351.0954	350.0881	350.0881	0.06	0.17	9	(M-H)-	86.33

--- End Of Report ---

Department of Chemistry Mass Spectrometry Laboratory

**Name**	N. Rana, West	**Sample Name**	nr-02-dp
**Data Filename**	19121923.d	**Instrument Name**	oaTOF6220
**Position**	-1	**Operator**	ami
**Acq Method**		**DA Method**	da ami low mass.m

NR-02 553

NR-02 ✓

User Spectra

Fragmentor Voltage	Source Type
200	Esi

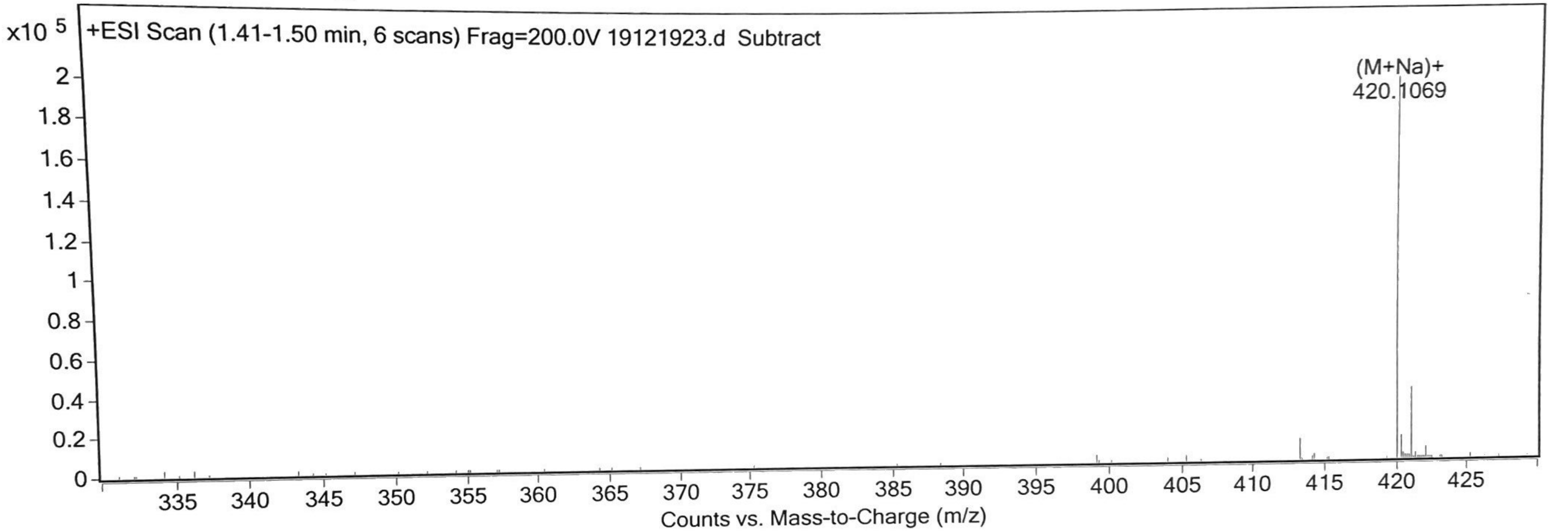

Oct 31

Compound 11

Formula Calculator Results

Formula	Ion Species	Mass	Calc. Mass	m/z	Calc. m/z	Diff (mDa)	Diff (ppm)	DBE	Ion	Score
C18 H20 F N O8	C18 H20 F N Na O8	397.1177	397.1173	420.1069	420.1065	-0.35	-0.83	9	(M+Na)+	93.59

--- End Of Report ---

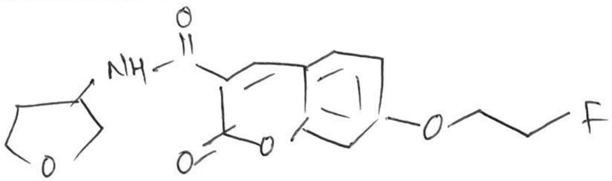

Supplement: Supplementary file 1 [file pharmaceutics-14-00828-s001.zip › pharmaceutics-1616877-supplementary.pdf]
